# Convalescent plasma for hospitalized patients with COVID-19: an open-label, randomized controlled trial

**DOI:** 10.1038/s41591-021-01488-2

**Published:** 2021-09-09

**Authors:** Philippe Bégin, Jeannie Callum, Erin Jamula, Richard Cook, Nancy M. Heddle, Alan Tinmouth, Michelle P. Zeller, Guillaume Beaudoin-Bussières, Luiz Amorim, Renée Bazin, Kent Cadogan Loftsgard, Richard Carl, Michaël Chassé, Melissa M. Cushing, Nick Daneman, Dana V. Devine, Jeannot Dumaresq, Dean A. Fergusson, Caroline Gabe, Marshall J. Glesby, Na Li, Yang Liu, Allison McGeer, Nancy Robitaille, Bruce S. Sachais, Damon C. Scales, Lisa Schwartz, Nadine Shehata, Alexis F. Turgeon, Heidi Wood, Ryan Zarychanski, Andrés Finzi, the CONCOR-1 Study Group, Danièle Marceau, Andy Huang, Holly Carr, Yulia Lin, Rosemarie Lall, Christopher Graham, Christine Arsenault, Valerie Sales, Davinder Sidhu, Makeda Semret, Caroline Hamm, Eneko Arhanchiague, Ziad Solh, Nadim Srour, Karim Soliman, Colin Yee, Vincent Laroche, Susan Nahirniak, Christina Greenaway, Menaka Pai, Andréanne Côté, Jennifer L. Y. Tsang, Christine Cserti-Gazdewich, Danielle Talbot, Sébastien Poulin, Rodrigo Guimaraes, Moira Rushton-Marovac, Alexandra Langlois, Shuoyan Ning, Andrew Shih, Mélissa Boileau, Harjot Singh, Donna Ledingham, Arjuna Ponnampalam, Matthew Yan, Oksana Prokopchuk-Gauk, André Poirier, Gabriel Girouard, Katerina Pavenski, Olivier Drouin, David Harris, Madeleine Durand, Emily Rimmer, Daniel Ovakim, François Ménard, Glenna Cuccarolo, Julie Carruthers, Kayla Lucier, Valérie Arsenault, Marie-Christine Auclair, Meda Avram, Michael Brassard, Sabrina Cerro, Véronica Martinez, Julie Morin, Marie Saint-Jacques, Maxime Veillette, Chantal Armali, Amie Kron, Dimpy Modi, Joanne Duncan, Pauline Justumus, Melanie St John, Geneviève St-Onge, Milena Hadzi-Tosev, Pierre-Marc Dion, Lawrence McGillivary, Andre Valleteau de Moulliac, Sheila A. Nyman, Stephanie Perilli, Paulette Jean Van Vliet, Shannon Lane, Katerina Pavenski, Rebecca Pereira, Emily Sirotich, Julie Abelson, Saara Greene, Aditi Khandelwal, Swarni Thakar, Sarah Longo, Sai Priya Anand, Mehdi Benlarbi, Catherine Bourassa, Marianne Boutin, Jade Descôteaux-Dinelle, Gabrielle Gendron-Lepage, Guillaume Goyette, Annemarie Laumaea, Halima Medjahed, Jérémie Prévost, Jonathan Richard, Daniel Kaufmann, Elsa Brunet-Ratnasingham, Nicolas Chaumont, Michael Drebot, Alyssia Robinson, Emelissa Mendoza, Kristina Dimitrova, Kathy Manguiat, Clark Phillipson, Michael Chan, David Evans, James Lin, Lucie Boyer, Marc Cloutier, Mathieu Drouin, Éric Ducas, Nathalie Dussault, Marie-Josée Fournier, Patricia Landy, Marie-Ève Nolin, Josée Perreault, Tony Tremblay, Ishac Nazy, Feng Xie, David Liu, Michelle Wong, Gus Silverio, Kristin Walkus, Mikaela Barton, Katherine Haveman, Darlene Mueller, Ashley Scott, Matthew Moher, Gordon Wood, Tracey Roarty, Fiona Auld, Gayle Carney, Virginia Thomson, Rodrigo Onell, Keith Walley, Katie Donohoe, Crystal Brunk, Geraldine Hernandez, Tina Jacobucci, Lynda Lazosky, Puneet Mann, Geeta Raval, Ligia Araujo Zampieri, Mypinder Sekhon, Alissa Wright, Nicola James, Gaby Chang, Roy Chen, Kanwal Deol, Jorell Gantioqui, Elyse Larsen, Namita Ramdin, Margaret Roche, Kristin Rosinski, Lawrence Sham, Michelle Storms, Mark Gillrie, Etienne Mahe, Deepa Suryanarayan, Alejandra Ugarte-Torres, Traci Robinson, Mitchell Gibbs, Julia Hewsgirard, Marnie Holmes, Joanna McCarthy, Meagan Ody, Karen Doucette, Wendy Sligl, Ashlesah Sonpar, Kimberley Robertson, Jeffrey Narayan, Leka Ravindran, Breanne Stewart, Lori Zapernick, Stephen Lee, Eric Sy, Alexander Wong, Karolina Gryzb, Sarah Craddock, Dennaye Fuchs, Danielle Myrah, Sana Sunny, Sheila Rutledge Harding, Siddarth Kogilwaimath, Nancy Hodgson, Dawn Johnson, Simona Meier, Kim Thomson, Amila Heendeniya, Brett Houston, Yoav Kenyan, Sylvain Lother, Kendiss Olafson, Barret Rush, Terry Wuerz, Dayna Solvason, Lisa Albensi, Soumya Alias, Nora Choi, Laura Curtis, Maureen Hutmacher, Hessam Kashani, Debra Lane, Nicole Marten, Tracey Pronyk-Ward, Lisa Rigaux, Rhonda Silva, Quinn Tays, Renuka Naidu, Jane Mathews, Margaret Mai, Victoria Miceli, Liz Molson, Gayathri Radhakrishnan, Linda Schaefer, Michel Haddad, Shannon Landry, Robert Chernish, Rebecca Kruisselbrink, Theresa Liu, Jayna Jeromin, Atif Siddiqui, Carla Girolametto, Kristin Krokoszynski, Cheryl Main, Alison Fox-Robichaud, Bram Rochwerg, Erjona Kruja, Dana Ellingham, Disha Sampat, Ngan Tang, Daniela Leto, Meera Karunakaran, Daniel Ricciuto, Kelly Fusco, Taneera Ghate, Holly Robinson, Ian Ball, Sarah Shalhoub, Marat Slessarev, Michael Silverman, Eni Nano, Tracey Bentall, Eileen Campbell, Jeffery Kinney, Seema Parvathy, Evridiki Fera, Anthony La Delfa, Jeya Nadarajah, Henry Solow, Edeliza Mendoza, Katrina Engel, Diana Monaco, Laura Kononow, Sutharsan Suntharalingam, Mike Fralick, Laveena Munshi, Samia Saeed, Omar Hajjaj, Elaine Hsu, Karim Ali, Erick Duan, George Farjou, Lorraine Jenson, Mary Salib, Lisa Patterson, Swati Anant, Josephine Ding, Jane Jomy, Pavani Das, Anna Geagea, Sarah Ingber, Elliot Owen, Alexandra Lostun, Tashea Albano, Antara Chatterjee, Manuel Giraldo, Jennifer Hickey, Ida Lee, Nea Okada, Nicholas Pasquale, Romina Ponzielli, Mary Rahmat, Shelina Sabur, Maria Schlag, Leonita Aguiar, Ashmina Damani, Suhyoung Hong, Mona Kokabi, Carolyn Perkins, Juthaporn Cowan, Tony Giulivi, Derek MacFadden, Joe Cyr, Amanda Pecarskie, Rebecca Porteous, Priscila Ogawa Vedder, Irene Watpool, Phil Berardi, Laith Bustani, Alison Graver, Akshai Iyengar, Magdalena Kisilewicz, Jake Majewski, Misha Marovac, Ruchi Murthy, Karan Sharma, Marina Walcer, Zain Chagla, Jason Cheung, Erick Duan, France Clarke, Karlo Matic, Manuel Giraldo, Jennifer Hickey, Ida Lee, Nea Okada, Nicholas Pasquale, Romina Ponzielli, Mary Rahmat, Shelina Sabur, Maria Schlag, Travis Carpenter, Kevin Schwartz, Paril Suthar, Aziz Jiwajee, Daniel Lindsay, Aftab Malik, Brandon Tse, Larissa Matukas, Joel Ray, Shirley Bell, Elizabeth Krok, Ray Guo, Susan John, Vishal Joshi, Jessica Keen, Chris Lazongas, Jacqueline Ostro, Kevin Shore, Jianmin Wang, Jincheol Choi, Pujitha Nallapati, Tina Irwin, Victor Wang, Petra Sheldrake, Neill Adhikari, Hannah Wunsch, Jacob Bailey, Harley Meirovich, Connie Colavecchia, Eiad Kahwash, Sachin Sud, Martin Romano, Bryan Coburn, Lorenzo Del Sorbo, John Granton, Shahid Husain, Jacob Pendergrast, Abdu Sharkawy, Liz Wilcox, Samia Saeed, Omar Hajjaj, Maria Kulikova, Sophia Massin, Wendy Kennette, Ian Mazzetti, Krista Naccarato, Grace Park, Alex Pennetti, Corrin Primeau, Cathy Vilag, Yves Lapointe, Anne-Sophie Lemay, Emmanuelle Duceppe, Benjamin Rioux-Massé, Cécile Tremblay, Pascale Arlotto, Claudia Bouchard, Stephanie Matte, Marc Messier-Peet, Charles-Langis Francoeur, François Lauzier, Guillaume Leblanc, David Bellemare, Ève Cloutier, Olivier Costerousse, Émilie Couillard Chénard, Rana Daher, Marjorie Daigle, Stéphanie Grenier, Gabrielle Guilbeault, Marie-Pier Rioux, Maude St-Onge, Antoine Tremblay, Brian Beaudoin, Luc Lanthier, Pierre Larrivée, Pierre-Aurèle Morin, Élaine Carbonneau, Robert Lacasse, Julie Autmizguine, Isabelle Boucoiran, Geneviève Du Pont-Thibodeau, Annie La Haye, Vincent Lague, Karine Léveillé, Caroline Quach-Thanh, Guillaume Émériaud, Philippe Jouvet, Élie Haddad, Camille Turgeon-Provost, Susan Fox, Diaraye Baldé, Lorraine Ménard, Suzanne Morissette, Miriam Schnorr-Meloche, Andrée-Anne Turcotte, Caroline Vallée, Stéphanie Castonguay, Tuyen Nguyen, Natalie Rivest, Marios Roussos, Esther Simoneau, Andreea Belecciu, Marie-Hélène Bouchard, Eric Daviau, Cynthia Martin, Nicole Sabourin, Solange Tremblay, Émilie Gagné, Nancy-Lisa Gagné, Julie Larouche, Vanessa Larouche, Véronick Tremblay, Vicky Tremblay, Pierre Blanchette, David Claveau, Marianne Lamarre, Danielle Tapps, Martin Albert, Anatolie Duca, Jean-Michel Leduc, Jean-Samuel Boudreault-Pedneault, Annie Barsalou, Suzanne Deschênes-Dion, Stéphanie Ibrahim, Stéphanie Ridyard, Julie Rousseau, Stéphane Ahern, Marie-Pier Arsenault, Simon-Frédéric Dufresne, Luigina Mollica, Hang Ting Wang, Soizic Beau, Dominique Beaupré, Marjolaine Dégarie, Iris Delorme, Melissa Farkas, Michel-Olivier Gratton, Arnaud Guertin, Guylaine Jalbert, Mélanie Meilleur, Charles Ratté Labrecque, Élaine Santos, Julie Trinh Lu, Julien Auger, Marie-Claude Lessard, Louay Mardini, Yves Pesant, Laurie Delves, Lisa Delves, Sophie Denault, Sofia Grigorova, Michelle Lambert, Nathalie Langille, Corinne Langlois, Caroline Rock, Yannick Sardin-Laframboise, Patrick Archambault, Joannie Bélanger-Pelletier, Estel Duquet-Deblois, Vanessa Dupuis-Picard, Yannick Hamelin, Samuel Leduc, Mélanie Richard, Marc Fortin, Philippe Gervais, Marie-Ève Boulay, Claudine Ferland, Jakie Guertin, Johane Lepage, Annie Roy, Sarit Assouline, Stephen Caplan, Ling Kong, Christina Canticas, Carley Mayhew, Johanne Ouedraogo, Tévy-Suzy Tep, Gerald Batist, Matthew Cheng, Marina Klein, Nadine Kronfli, Patricia Pelletier, Salman Qureshi, Donald Vinh, Robert Dziarmaga, Hansi Peiris, Karène Proulx-Boucher, Jonathan Roger, Molly-Ann Rothschild, Chung-Yan Yuen, Sapha Barkati, Jean-Pierre Routy, Sondra Sinanan-Pelletier, Rémi LeBlanc, Eve St-Hilaire, Patrick Thibeault, Karine Morin, Gilberte Caissie, Jackie Caissie Collette, Line Daigle, Mélissa Daigle, Bianca Gendron, Nathalie Godin, Angela Lapointe, Gabrielle Moreau, Lola Ouellette-Bernier, Joanne Rockburn, Brigitte Sonier-Ferguson, Christine Wilson, Robert DeSimone, Grant Ellsworth, Rebecca Fry, Noah Goss, Roy Gulick, Carlos Vaamonde, Timothy Wilkin, Celine Arar, Jonathan Berardi, Dennis Chen, Cristina Garcia-Miller, Arthur Goldbach, Lauren Gripp, Danielle Hayden, Kathleen Kane, Jiamin Li, Kinge-Ann Marcelin, Christina Megill, Meredith Nelson, Ailema Paguntalan, Gabriel Raab, Gianna Resso, Roxanne Rosario, Noah Rossen, Shoran Tamura, Ethan Zhao, Cheryl Goss, Young Kim, Eshan Patel, Sonal Paul, Tiffany Romero, Naima ElBadri, Lina Flores, Tricia Sandoval, Shashi Kapadia, Ljiljana Vasovic, Shanna-Kay Griffiths, Daniel Alvarado, Fiona Goudy, Melissa Lewis, Marina Loizou, Rita Louie, Chantale Pambrun, Sylvia Torrance, Steven Drews, Janet McManus, Oriela Cuevas, Wanda Lafresne, Patrizia Ruoso, Christine Shin, Tony Steed, Rachel Ward, Isabelle Allard, Marc Germain, Sébastien Girard, Éric Parent, Claudia-Mireille Pigeon, Maria Esther Lopes, Margarida Pêcego, Natalia Rosario, Carlos Alexandre da Costa Silva, Thais Oliveira, Maria Cristina Lopes, Sheila Mateos, Lucette Hall, Sarai Paradiso, Donna Strauss, Donald M. Arnold

**Affiliations:** 1 Department of Pediatrics, CHU Sainte-Justine, Montreal, Quebec Canada; 2 Department of Medicine, Centre Hospitalier de l’Université de Montréal, Montreal, Quebec Canada; 3 Department of Pathology and Molecular Medicine, Kingston Health Sciences Centre and Queen’s University, Kingston, Ontario Canada; 4 Department of Laboratory Medicine and Molecular Diagnostics, Sunnybrook Health Sciences Centre, Toronto, Ontario Canada; 5 Department of Laboratory Medicine and Pathobiology, University of Toronto, Toronto, Ontario Canada; 6 Canadian Blood Services, Ottawa, Ontario Canada; 7 McMaster Centre for Transfusion Research, McMaster University, Hamilton, Ontario Canada; 8 Department of Statistics and Actuarial Science, University of Waterloo, Waterloo, Ontario Canada; 9 Department of Medicine, McMaster University, Hamilton, Ontario Canada; 10 Department of Medicine, University of Ottawa, Ottawa, Ontario Canada; 11 Ottawa Hospital Centre for Transfusion Research, Ottawa Hospital Research Institute, Ottawa, Ontario Canada; 12 Département de Microbiologie, Infectiologie et Immunologie, Université de Montréal, Montreal, Quebec Canada; 13 CHUM Research Center, Montreal, Quebec Canada; 14 Hemorio, Hospital and Regional Blood Center, Rio de Janeiro, Brazil; 15 Héma-Québec, Medical Affairs and Innovation, Quebec City, Quebec Canada; 16 CONCOR-1 Community Advisory Committee representative, Montreal, Quebec Canada; 17 Patient representative, Montreal, Quebec Canada; 18 Innovation Hub, Centre de Recherche du Centre Hospitalier de l’Université de Montréal, Montreal, Quebec Canada; 19 Transfusion Medicine and Cellular Therapy, New York-Presbyterian, New York, NY USA; 20 Department of Pathology and Laboratory Medicine, Weill Cornell Medicine, New York, NY USA; 21 Department of Medicine, Division of Infectious Diseases, Sunnybrook Health Sciences Centre, University of Toronto, Toronto, Ontario Canada; 22 Canadian Blood Services, Vancouver, British Columbia Canada; 23 Department of Pathology and Laboratory Medicine, University of British Columbia, Vancouver, British Columbia Canada; 24 Département de médecine, CISSS de Chaudière-Appalaches, Lévis, Quebec Canada; 25 Département de microbiologie-infectiologie et d’immunologie, Faculté de Médecine, Université Laval, Quebec City, Quebec Canada; 26 Clinical Epidemiology Program, Ottawa Hospital Research Institute, Ottawa, Ontario Canada; 27 Division of Infectious Diseases, Weill Cornell Medical College, New York, NY USA; 28 Department of Community Health Sciences, University of Calgary, Calgary, Alberta Canada; 29 Department of Computing and Software, McMaster University, Hamilton, Ontario Canada; 30 Department of Microbiology, Sinai Health System, Toronto, Ontario Canada; 31 Department of Laboratory Medicine and Pathobiology and Dalla Lana School of Public Health, University of Toronto, Toronto, Ontario Canada; 32 Héma-Québec, Montreal, Quebec Canada; 33 Division of Hematology and Oncology, Department of Pediatrics, CHU Sainte-Justine, Montreal, Quebec Canada; 34 Department of Pediatrics, Université de Montréal, Montreal, Quebec Canada; 35 New York Blood Center Enterprises, New York, NY USA; 36 Department of Critical Care Medicine, Sunnybrook Health Sciences Centre, Toronto, Ontario Canada; 37 Department of Medicine, Interdepartmental Division of Critical Care Medicine, University of Toronto, Toronto, Ontario Canada; 38 Department of Health Research Methods, Evidence & Impact, Faculty of Health Sciences, McMaster University, Hamilton, Ontario Canada; 39 Departments of Medicine, Laboratory Medicine and Pathobiology, Institute of Health Policy Management and Evaluation, University of Toronto, Toronto, Ontario Canada; 40 Division of Hematology, Mount Sinai Hospital, Toronto, Ontario Canada; 41 Department of Anesthesiology and Critical Care Medicine, Division of Critical Care Medicine, Faculty of Medicine, Université Laval, Quebec City, Quebec Canada; 42 CHU de Québec—Université Laval Research Centre, Population Health and Optimal Health Practices Research Unit, Trauma–Emergency–Critical Care Medicine, Université Laval, Quebec City, Quebec Canada; 43 Zoonotic Diseases and Special Pathogens, National Microbiology Laboratory, Public Health Agency of Canada, Winnipeg, Manitoba Canada; 44 Department of Internal Medicine, Sections of Hematology/Medical Oncology and Critical Care, University of Manitoba, Winnipeg, Manitoba Canada; 45 New York-Presbyterian Brooklyn Methodist Hospital, New York, NY USA; 46 Sunnybrook Health Sciences Centre, Toronto, Ontario Canada; 47 Scarborough Health Network, Scarborough, Ontario Canada; 48 Trillium Health Partners, Mississauga, Ontario Canada; 49 Hôpital du Sacré-Cœur-de-Montréal, Montreal, Quebec Canada; 50 Markham Stouffville Hospital, Markham, Ontario Canada; 51 Foothills Hospital, Peter Lougheed Centre, Rockyview General Hospital, Calgary, Alberta Canada; 52 McGill University Hospital Center, Montreal, Quebec Canada; 53 Windsor Regional Hospital, Windsor, Ontario Canada; 54 North York General Hospital, North York, Ontario Canada; 55 London Health Sciences Centre, London, Ontario Canada; 56 CISSS Montérégie-Centre, Hôpital Charles-Lemoyne, Greenfield Park, Quebec Canada; 57 Lakeridge Health, Oshawa and Ajax, Ontario Canada; 58 Grand River Hospital and St. Mary’s General Hospital, Kitchener, Ontario Canada; 59 University of Alberta, Royal Alexandra Hospital, Edmonton, Alberta Canada; 60 Jewish General Hospital, Montreal, Quebec Canada; 61 Hamilton General Hospital, Hamilton, Ontario Canada; 62 Institut de Cardiologie et Pneumologie de Québec, Université Laval, Quebec City, Quebec Canada; 63 Niagara Health System, St. Catharines Site, St. Catharines, Ontario Canada; 64 University Health Network, Toronto, Ontario Canada; 65 Hôpital Cité-de-la-Santé de Laval, Laval, Quebec Canada; 66 Hôpital régional de St-Jérôme, St-Jérôme, Quebec Canada; 67 Queensway Carleton Hospital, Ottawa, Ontario Canada; 68 CHU de Sherbrooke, Sherbrooke, Quebec Canada; 69 St. Joseph’s Healthcare, Hamilton, Ontario Canada; 70 Vancouver General Hospital, Vancouver, British Columbia Canada; 71 Hôpital Maisonneuve-Rosemont, Montreal, Quebec Canada; 72 New York-Presbyterian Lower Manhattan Hospital, New York, NY USA; 73 Pasqua Hospital, Regina General Hospital, Regina, Saskatchewan Canada; 74 Grace General Hospital, Health Sciences Centre, St. Boniface General Hospital, Winnipeg, Manitoba Canada; 75 Abbotsford Regional Hospital, Abbotsford, British Columbia Canada; 76 Royal University Hospital, St. Paul’s Hospital, Saskatoon, Saskatchewan Canada; 77 Hôpital de Trois-Rivières, Trois-Rivières, Quebec Canada; 78 Vitalité Health Network, Moncton, New Brunswick Canada; 79 St. Joseph’s Health Centre, Toronto, Ontario Canada; 80 St. Paul’s Hospital, Vancouver, British Columbia Canada; 81 Royal Jubilee Hospital & Victoria General Hospital, Victoria, British Columbia Canada; 82 Hôpital de Chicoutimi, Chicoutimi, Quebec Canada; 83 Bluewater Health, Sarnia, Ontario Canada; 84 University of Toronto Quality in Utilization, Education and Safety in Transfusion (QUEST) Research Program, Sunnybrook Health Sciences Centre, Toronto, Ontario Canada; 85 Medical Microbiology & Immunology, University of Alberta, Edmonton, Alberta Canada; 86 Juravinski Hospital, Hamilton, Ontario Canada

**Keywords:** Randomized controlled trials, Antibodies, Viral infection, Antibody therapy

## Abstract

The efficacy of convalescent plasma for coronavirus disease 2019 (COVID-19) is unclear. Although most randomized controlled trials have shown negative results, uncontrolled studies have suggested that the antibody content could influence patient outcomes. We conducted an open-label, randomized controlled trial of convalescent plasma for adults with COVID-19 receiving oxygen within 12 d of respiratory symptom onset (NCT04348656). Patients were allocated 2:1 to 500 ml of convalescent plasma or standard of care. The composite primary outcome was intubation or death by 30 d. Exploratory analyses of the effect of convalescent plasma antibodies on the primary outcome was assessed by logistic regression. The trial was terminated at 78% of planned enrollment after meeting stopping criteria for futility. In total, 940 patients were randomized, and 921 patients were included in the intention-to-treat analysis. Intubation or death occurred in 199/614 (32.4%) patients in the convalescent plasma arm and 86/307 (28.0%) patients in the standard of care arm—relative risk (RR) = 1.16 (95% confidence interval (CI) 0.94–1.43, *P* = 0.18). Patients in the convalescent plasma arm had more serious adverse events (33.4% versus 26.4%; RR = 1.27, 95% CI 1.02–1.57, *P* = 0.034). The antibody content significantly modulated the therapeutic effect of convalescent plasma. In multivariate analysis, each standardized log increase in neutralization or antibody-dependent cellular cytotoxicity independently reduced the potential harmful effect of plasma (odds ratio (OR) = 0.74, 95% CI 0.57–0.95 and OR = 0.66, 95% CI 0.50–0.87, respectively), whereas IgG against the full transmembrane spike protein increased it (OR = 1.53, 95% CI 1.14–2.05). Convalescent plasma did not reduce the risk of intubation or death at 30 d in hospitalized patients with COVID-19. Transfusion of convalescent plasma with unfavorable antibody profiles could be associated with worse clinical outcomes compared to standard care.

## Abstract (web summary)

A randomized trial in patients hospitalized with COVID-19 showed no benefit and potentially increased harm associated with the use of convalescent plasma, with subgroup analyses suggesting that the antibody profile in donor plasma is critical in determining clinical outcomes.

## Main

The immune response after severe acute respiratory syndrome coronavirus 2 (SARS-CoV-2) infection results in the formation of antibodies that can interfere with viral replication and infection of host cells in over 95% of patients^1^. Based on previous experience in other viral infections^2^, the use of convalescent plasma has been proposed as a therapeutic form of passive immunization for patients with acute COVID-19 (refs. ^3,4^). Early in the pandemic, several small randomized trials found no difference in clinical outcomes^5–8^. In the United States, an Extended Access Program outside of a controlled trial led to the use of convalescent plasma in over half a million patients. Data from these patients showed that the transfusion of plasma with high anti-SARS-CoV-2 antibody levels was associated with a lower risk of death in non-intubated patients compared to lower antibody levels; however, this study lacked a control group^9^. The RECOVERY trial was a large randomized trial in 11,558 hospitalized patients that found that the risk of death after the administration of high-titer plasma was not different from standard of care^10^.

The Convalescent Plasma for COVID-19 Respiratory Illness (CONCOR-1) trial was a multi-center, international, open-label, randomized controlled trial designed to assess the effectiveness and safety of COVID-19 convalescent plasma in hospitalized patients. The trial used plasma collected from four blood suppliers with a range of anti-SARS-CoV-2 antibody levels. The variability in antibody titers allowed for a characterization of the effect-modifying role of functional and quantitative antibodies on the primary outcome (intubation or death at 30 d).

## Results

### Patients

**Fig. 1:**
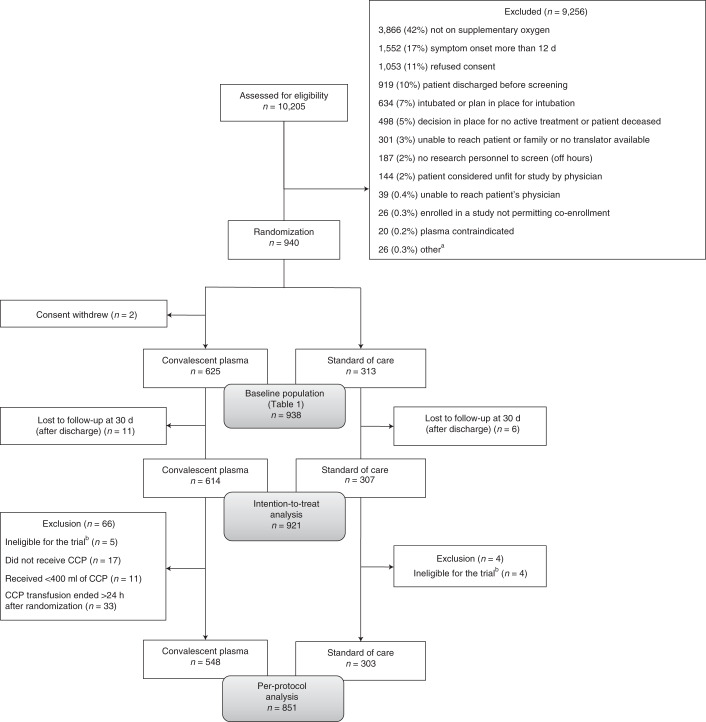
Enrollment, randomization and follow-up. Patient flow in the CONCOR-1 study detailing the intention-to-treat population, per-protocol analysis population and excluded patients. Other^a^, *n* = 26: <16 years of age (*n* = 13), <18 years of age (*n* = 5), ABO-compatible plasma unavailable (*n* = 5) and other (*n* = 3). ^b^Includes not receiving supplemental oxygen at the time of randomization (but on oxygen at screening) and any symptom onset >12 d before randomization for protocol version 5.0 or earlier.

This trial was stopped at the planned interim analysis because the conditional power estimate was 1.6% (below the stopping criterion of 20%). Between 14 May 2020 and 29 January 2021, 940 patients were randomized (2:1) to convalescent plasma or standard of care in 72 hospital sites in Canada, the United States and Brazil (Fig. 1 and Supplementary Table 1). Two patients randomized to plasma withdrew consent before treatment. Demographics of the baseline study population (*n* = 938) were balanced between groups for all study populations (Table 1 and Supplementary Tables 2 and 3). The median age was 69 years, with 59% male and 41% female, and the median time from the onset of any COVID-19 symptom was 8 d (interquartile range (IQR), 5–10 d). Most patients (*n* = 766, 81.7%) were receiving systemic corticosteroids at the time of enrolment. Seventeen patients were lost to follow-up between discharge and day 30, precluding assessment of the primary outcome.

**Table 1:** Characteristics of the baseline study population (excluding two patients who withdrew consent). Categorical data are presented as number (percentage) and continuous variables as mean ± standard deviation and median (IQR)

Characteristic	Convalescent plasma, *n* = 625	Standard of care, *n* = 313	Overall, *n* = 938
Age, years	67.7 ± 16.0; 69 (58, 80)	67.1 ± 14.8; 68 (58, 78)	67.5 ± 15.6; 69 (58, 79)
≥60 years	438 (70.1)	218 (69.6)	656 (69.9)
Sex			
Male	369 (59.0)	185 (59.1)	554 (59.1)
Female	256 (41.0)	128 (40.9)	384 (40.9)
Pregnant at randomization	4 (0.6)	1 (0.3)	5 (0.5)
Ethnicity			
White	305 (48.8)	153 (48.9)	458 (48.8)
Asian	104 (16.6)	46 (14.7)	150 (16.0)
Hispanic or Latino	34 (5.4)	9 (2.9)	43 (4.6)
Black	25 (4.0)	11 (3.5)	36 (3.8)
Other	38 (6.1)	28 (8.9)	66 (7.0)
Unknown	119 (19.0)	66 (21.1)	185 (19.7)
ABO blood group			
O	270 (43.2)	113 (36.1)	383 (40.8)
A	235 (37.6)	121 (38.7)	356 (38.0)
B	89 (14.2)	57 (18.2)	146 (15.6)
AB	31 (5.0)	22 (7.0)	53 (5.7)
BMI (kg m^−^^2^)	30.0 ± 7.5; 29 (25, 33)	30.0 ± 7.4; 29 (25, 33)	30.0 ± 7.4; 29 (25, 33)
BMI < 30	256 (41.0)	123 (39.3)	379 (40.4)
BMI ≥ 30	198 (31.7)	102 (32.6)	300 (32.0)
Unknown	171 (27.4)	88 (28.1)	259 (27.6)
Presence of comorbidity			
Diabetes	220 (35.2)	108 (34.5)	328 (35.0)
Cardiac disease	385 (61.6)	197 (62.9)	582 (62.0)
Baseline respiratory diseases	147 (23.5)	79 (25.2)	226 (24.1)
Abnormal CT chest or chest X-ray result before randomization	563 (90.1)	266 (85.0)	829 (88.4)
Medication for other research study at baseline	53 (8.5)	41 (13.1)	94 (10.0)
Medication for COVID-19 at baseline			
Azithromycin	279 (44.6)	137 (43.8)	416 (44.3)
Other antibiotics	405 (64.8)	186 (59.4)	591 (63.0)
Systemic corticosteroids	496 (79.4)	258 (82.4)	754 (80.4)
Antiviral medications	165 (26.4)	80 (25.6)	245 (26.1)
Anticoagulants	355 (56.8)	180 (57.5)	535 (57.0)
Other COVID-19 medications	79 (12.6)	3 9(12.5)	118 (12.6)
Medication not for COVID-19 at baseline			
ACE inhibitor	85 (13.6)	63 (20.1)	148 (15.8)
ACE receptor blocker	77 (12.3)	47 (15.0)	124 (13.2)
Non-steroidal anti-inflammatory drugs	77 (12.3)	52 (16.6)	129 (13.8)
Colchicine	5 (0.8)	2 (0.6)	7 (0.7)
Systemic corticosteroids	61 (9.8)	35 (11.2)	96 (10.2)
Inhaled corticosteroids	84 (13.4)	42 (13.4)	126 (13.4)
Immunomodulatory agents	22 (3.5)	18 (5.8)	40 (4.3)
Anticoagulants	135 (21.6)	64 (20.4)	199 (21.2)
Systemic corticosteroids at baseline	504 (80.6)	262 (83.7)	766 (81.7)
Oxygen status at baseline (FiO_2_)	49.5 ± 25.2; 40 (30, 65)	48.8 ± 25.1; 40 (30, 60)	49.3 ± 25.2; 40 (30, 60)
Time from any symptom onset to randomization (d)	8.0 ± 3.8; 8 (5, 10)	7.8 ± 3.4; 8 (5, 10)	7.9 ± 3.7; 8 (5, 10)
Time from COVID-19 diagnosis^a^ to randomization (d)	4.9 ± 3.6; 4 (2, 7)	5.1 ± 4.4; 4 (2, 7)	5.0 ± 3.9; 4 (2, 7)
Location at randomization			
Ward	505 (80.8)	260 (83.1)	765 (81.6)
ICU	120 (19.2)	53 (16.9)	173 (18.4)
Enrolled in other clinical trials	168 (26.9)	98 (31.3)	266 (28.4)

^a^Day of positive COVID-19 test

ACE, angiotensin-converting enzyme; BMI, body mass index; CT, computed tomography; FiO_2_, fraction of inhaled oxygen; ICU, intensive care unit.

### Primary outcome

**Fig. 2:**
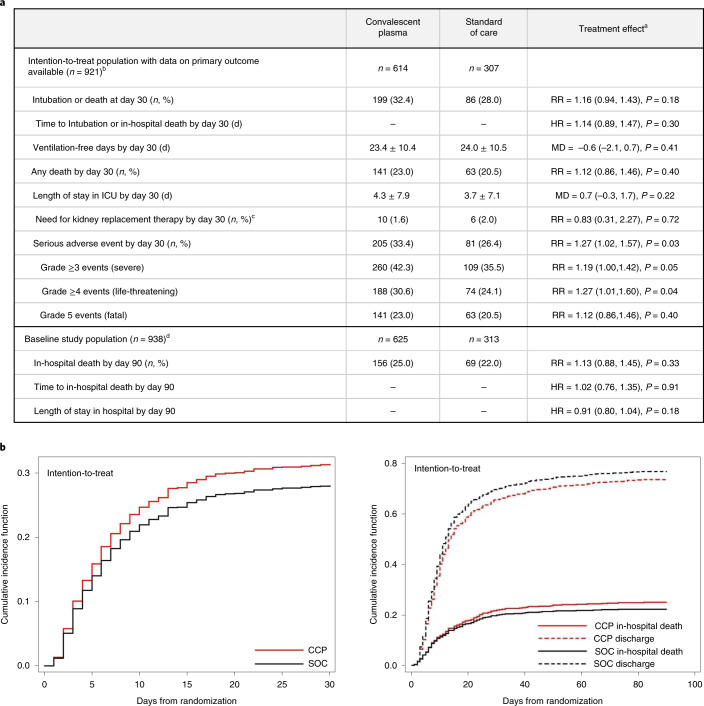
Study outcomes. **a**, Patient outcomes for the primary and secondary endpoints. **b**, Cumulative incidence functions of the primary outcome (intubation or death) by day 30 and of in-hospital death by day 90. ^a^RR and 95% CI; hazard ratio ((HR), 95% CI); and mean difference ((MD), with 95% CI based on robust bootstrap standard errors). ^b^Seventeen patients were discharged before day 30 and were lost to follow-up at 30 d, and two withdrew consent before day 30; thus, outcomes collected at day 30 (primary outcome and some other secondary outcomes for day 30) were missing. ^c^Excluding 11 patients on chronic kidney replacement therapy at baseline. ^d^Intention-to-treat survival analyses were based on the complete baseline population (940 randomized patients minus two patients who withdrew consent).

In the intention-to-treat population (*n* = 921), intubation or death occurred in 199 (32.4%) of 614 patients in the convalescent plasma group and 86 (28.0%) of 307 patients in the standard of care group (RR = 1.16, 95% CI 0.94–1.43, *P* = 0.18) (Fig. 2a). The time to intubation or death was not significantly different between groups (Fig. 2b). In the per-protocol analysis (*n* = 851), intubation or death occurred in 167 (30.5%) of 548 patients in the convalescent plasma group and 85 (28.1%) of 303 patients in the standard of care group (RR = 1.09, 95% CI 0.87–1.35, *P* = 0.46) (Supplementary Table 4).

### Secondary efficacy outcomes and subgroup analyses

**Fig. 3:**
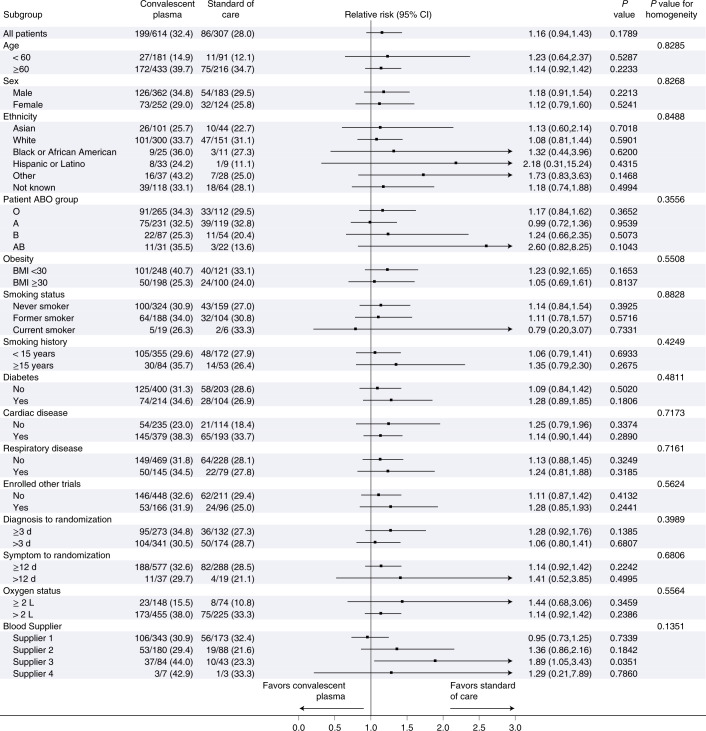
Subgroup analyses. Forest plots are presented for the subgroup analyses for the intention-to-treat population. *P* values for RR and homogeneity are two sided without adjustment for multiple comparisons. BMI, body mass index.

**Extended Data Fig. 1:**
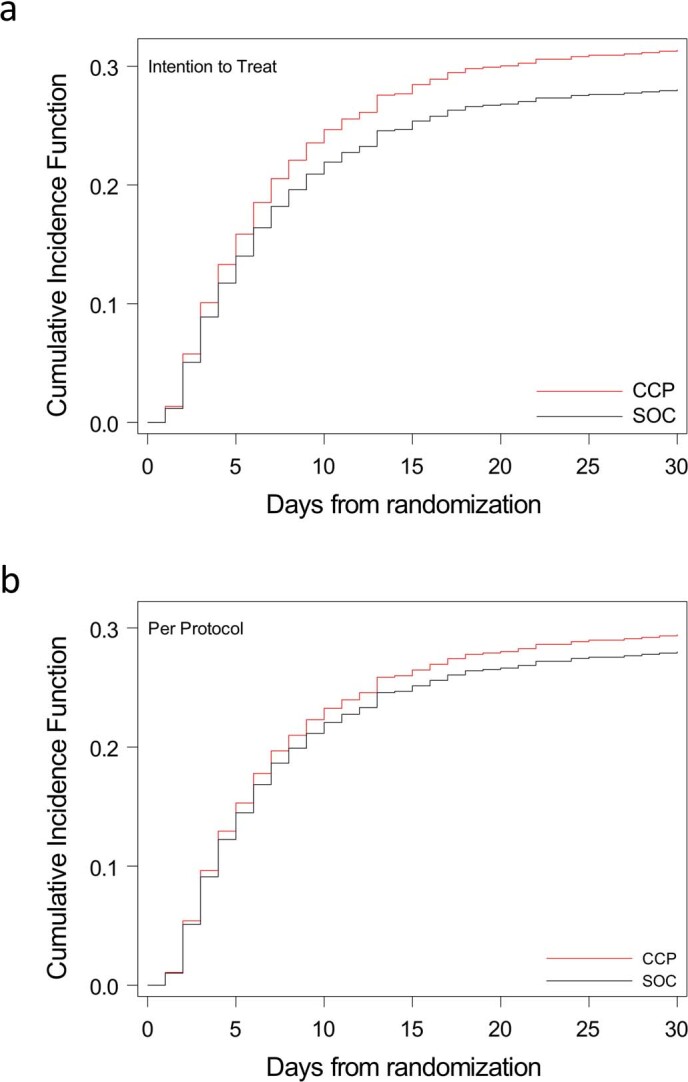
Cumulative incidence functions of intubation or in-hospital death by day 30. Panel A presents the intention-to-treat population and panel B presents the per protocol population.

**Extended Data Fig. 2:**
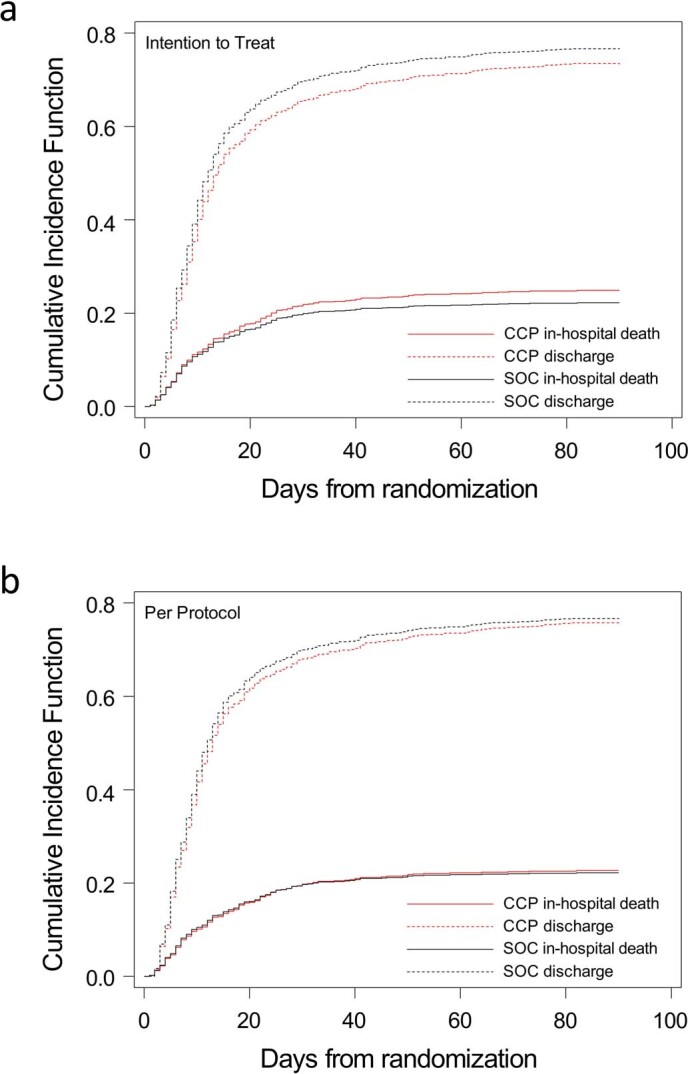
Cumulative incidence functions of in-hospital death by day 90. Panel A presents the intention-to-treat population and panel B presents the per protocol population.

**Extended Data Fig. 3:**
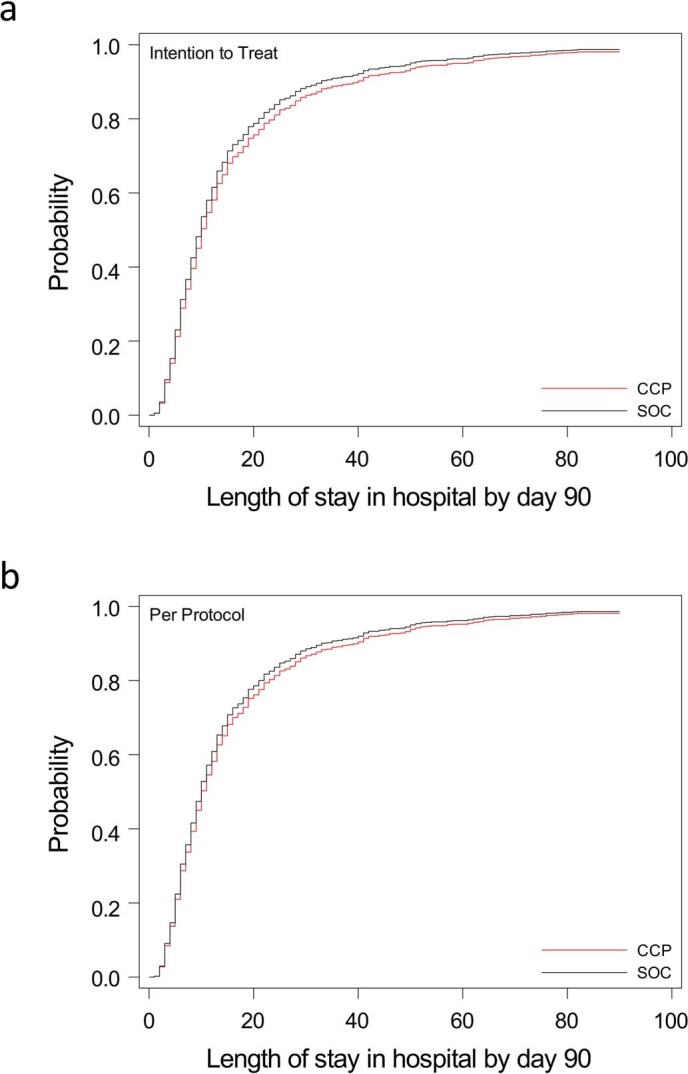
Kaplan-Meier estimate of distribution of length of stay in hospital by day 90. Panel A presents the intention-to-treat population and panel B presents the per protocol population.

**Extended Data Fig. 4:**
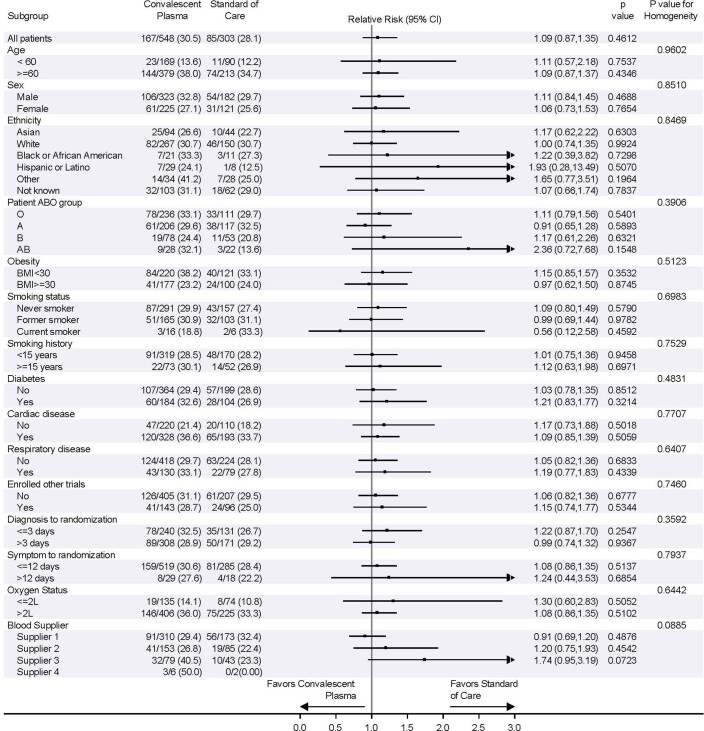
Subgroup analysis for the per-protocol population. P-values for relative risk and homogeneity are two-sided without adjustment for multiple comparisons. BMI: Body mass index.

**Extended Data Fig. 5:**
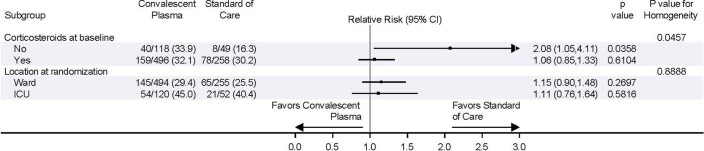
Post-hoc subgroup analyses for the intention-to-treat population. Subgroups based on corticosteroid use and location at time of randomizations were added post-hoc at time of review. P-values for relative risk and homogeneity are two-sided without adjustment for multiple comparisons.

**Extended Data Fig. 6:**
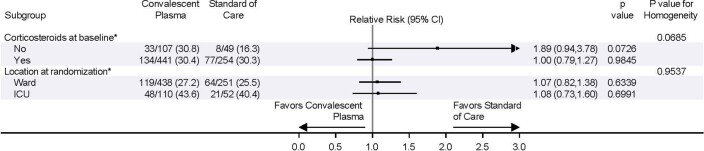
Post-hoc subgroup analyses for the per-protocol population. Subgroups based on corticosteroid use and location at time of randomizations were added post-hoc at time of review. P-values for relative risk and homogeneity are two-sided without adjustment for multiple comparisons.

Secondary outcomes for the intention-to-treat population are shown in Fig. 2a. There were no differences in mortality or intubation or other secondary efficacy outcomes. Similarly, in the per-protocol analysis, there were no differences in the secondary efficacy outcomes (Supplementary Table 4 and Extended Data Figs. 1–3). No significant differences were observed in most subgroups, including time from diagnosis to randomization <3 d for both the intention-to-treat (Fig. 3) and per-protocol (Extended Data Fig. 4) populations. The subgroup of patients served by blood supplier 3 (Fig. 3) and the post hoc subgroup of patients who were not receiving corticosteroids (Extended Data Figs. 5 and 6) had worse outcomes with convalescent plasma compared to standard of care.

### Safety

Serious adverse events occurred in 205 (33.4%) of 614 patients in the convalescent plasma arm compared to 81 (26.4%) of 307 patients in the standard of care arm for the intention-to-treat population (RR = 1.27, 95% CI 1.02–1.57, *P* = 0.034; Fig. 2a and Supplementary Tables 4–6). Most of these events were worsening hypoxemia and respiratory failure. Transfusion-related complications were recorded in 35 (5.7%) of 614 patients in the convalescent plasma group (Supplementary Tables 7 and 8). Of the 35 reactions, four were life-threatening (two transfusion-associated circulatory overload, one possible transfusion-related acute lung injury and one transfusion-associated dyspnea), and none was fatal. Thirteen of the 35 reactions were classified as transfusion-associated dyspnea. Two patients underwent serological investigation for transfusion-related acute lung injury (both negative).

### Effect-modifying role of antibodies in convalescent plasma

**Fig. 4:**
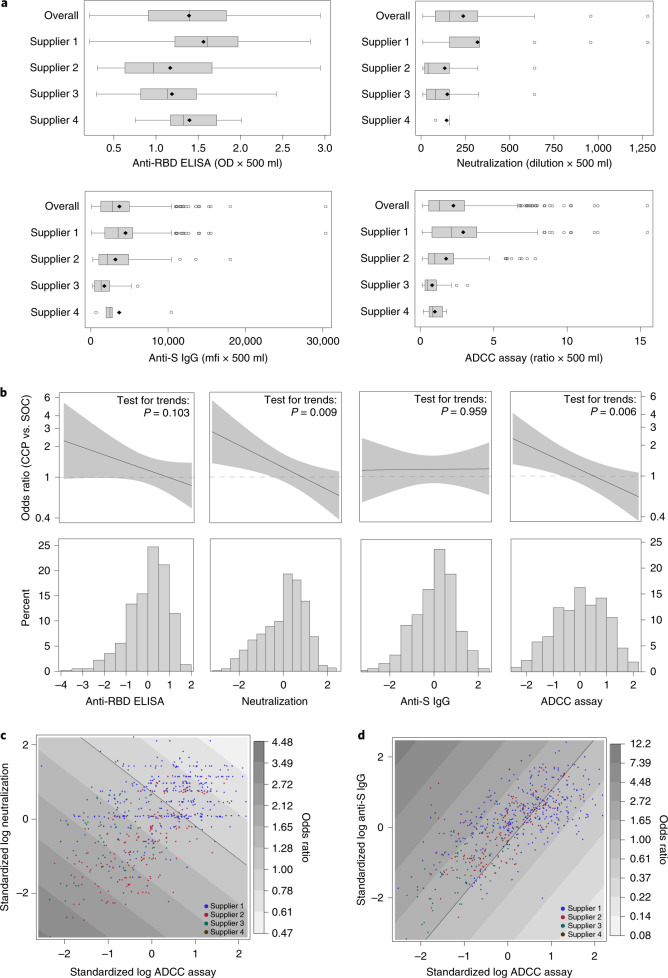
The effect-modifying role of convalescent plasma antibody content for the primary outcome. **a**, Absolute antibody amounts transfused per patient (*n* = 597) in the CCP arm for each marker, expressed as the product of volume and concentration. Center line: median; box limits: 25th and 75th percentiles; whiskers: 1.5× IQR; points: outliers. **b**, Effect-modifying role of CCP content for the primary outcome for each marker. The top row presents the trends in CCP effect compared to SOC as a function of the marker value, along with 95% CIs. Marker values are expressed as standard deviations of log values centered around the mean (standardized log). The horizontal dotted line represents CCP with no effect (OR = 1). The *P* values (two-sided test for trend without adjustment for multiple comparisons) refer to the effect modification observed with each marker (Supplementary Table 10). The histograms present the frequency distribution by marker. **c**,**d**, Contour plots of the OR for the primary outcome as a function of marker combinations. Overlaid data points indicate the value of the two markers for each CCP transfusion. Mfi, mean fluorescence intensity; OD, optical density; S, SARS-CoV-2 spike protein; SOC, standard of care.

**Extended Data Fig. 7:**
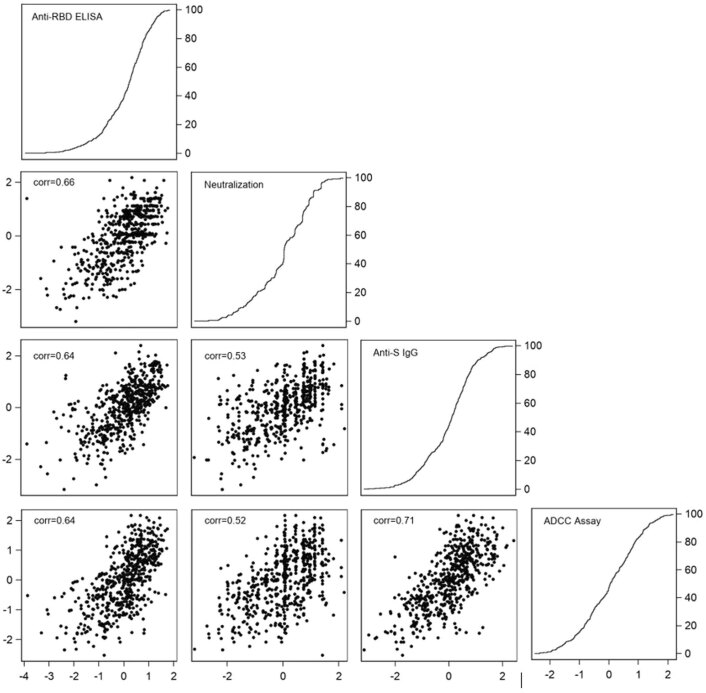
Pairwise scatter plots of plasma antibody markers and empirical distribution functions. Markers (log transformed and standardized) include antibody (IgM,IgA,IgG) against the receptor binding domain (anti-RBD) by ELISA, plaque reduction neutralization test, IgG antibody against the full transmembrane Spike protein (anti-S IgG) by flow cytometry and the antibody-dependent cellular cytotoxicity (ADCC) assay.corr: Pearson correlation coefficients of pair of antibody markers.

The distributions of antibodies in convalescent plasma units varied by blood supplier (Fig. 4, Supplementary Table 9 and Extended Data Fig. 7); therefore, antibody analyses controlled for supplier to address possible confounding. Transfusion of convalescent plasma with average (log-transformed) levels of antibody-dependent cellular cytotoxicity (ADCC) yielded an OR of 1.16 (95% CI 0.85–1.57) for the primary outcome relative to standard of care. Each one-unit increase in the standardized log-transformed ADCC was associated with a 24% reduction in the OR of the treatment effect (OR = 0.76, 95% CI 0.62–0.92) (Fig. 4 and Supplementary Table 10). This effect-modifying role was also significant for the neutralization test (OR = 0.77, 95% CI 0.63–0.94) but not for anti-receptor-binding domain (RBD) enzyme-linked immunosorbent assay (ELISA) (OR = 0.84, 95% CI 0.69–1.03) or IgG against the full transmembrane spike (OR = 1.01, 95% CI 0.82–1.23).

**Extended Data Fig. 8:**
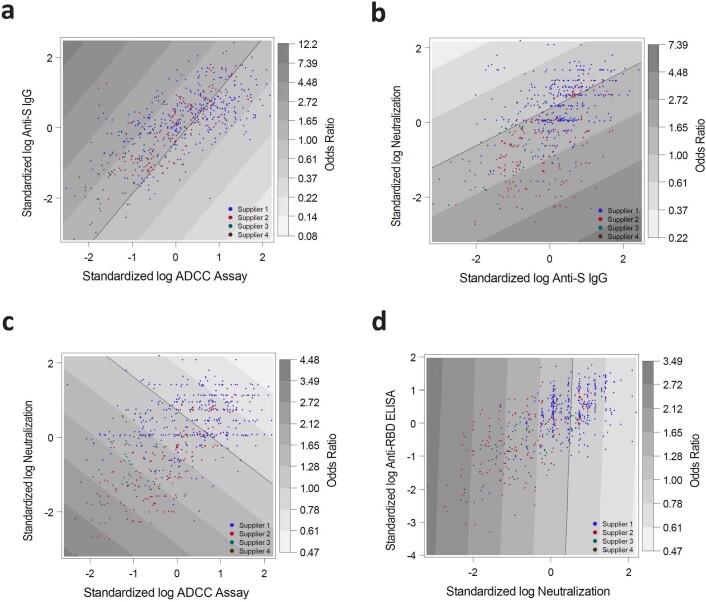
Contour plots of the joint effect-modifying role of antibody markers for convalescent plasma versus standard of care on the composite endpoint of intubation or death. The contours convey pairwise combinations of antibody markers yielding similar odds ratios for the CCP effect with the black line corresponding to an odds ratio of 1 (that is no effect of CCP). Data points for individual patients are overlaid with colours denoting the blood supply centre. Contours were obtained from fitting generalized additive logistic regression models for the primary outcome adjusting for blood supply center, treatment and the log transformed and standardized biomarkers - smoothing splines were used to relax linearity assumptions. The contour lines with positive slope suggest combinations of high (or low) values for both markers yield similar effects of CCP; the contour lines with negative slopes suggest high values of both markers yield strong CCP effects. For the combination of anti-S IgG with ADCC or anti-S IgG with anti-RBD, the general additive logistic regression models led to a complex equation that was not statistically significant nor clinical interpretable. These combinations were therefore excluded from this figure.

When all four serologic markers were included in the multivariate model, each one-unit increase in the standardized log-transformed anti-spike IgG marker was associated with a 53% increase in the OR for the deleterious effect of convalescent plasma on the primary outcome (OR = 1.53, 95% CI 1.14–2.05); increases in ADCC and neutralization independently improved the effect of CCP (OR = 0.66, 95% CI 0.50–0.87 and OR = 0.74, 95% CI 0.57–0.95, respectively), whereas levels of anti-RBD antibodies had no effect-modifying role (OR = 1.02, 95% CI 0.76–1.38) (Supplementary Table 10). There was no evidence of significant interaction among the four serologic measures in the general additive model (Fig. 4 and Extended Data Fig. 8).

### Meta-analysis

**Fig. 5:**
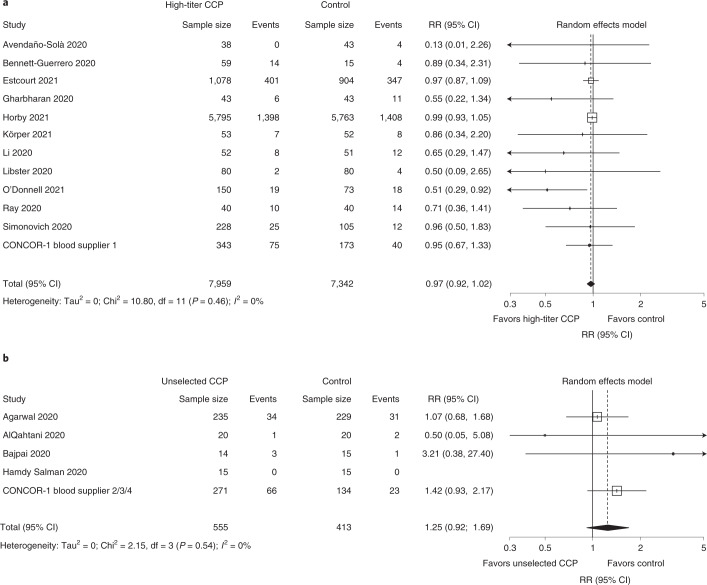
Meta-analysis of mortality at 30 d in CONCOR-1 and other trials according to convalescent plasma selection strategy. **a**, Meta-analysis of trials that used high-titer plasma. **b**, Meta-analysis of trials that used a mix of low-, medium- and high-titer plasma. df, degrees of freedom.

Of the 15 other reported randomized trials, 11 used only high-titer plasma^5,7,10–18^, and four applied less stringent plasma selection criteria, allowing for variable plasma titers^6,19–21^. Including the results from CONCOR-1, a total of 15,301 patients participated in trials using high-titer plasma, and 968 participated in trials applying less stringent criteria. The summary estimates for the RR of mortality in high-titer plasma trials was 0.97 (95% CI 0.92–1.02) compared to 1.25 (95% CI 0.92–1.69) in trials using unselected convalescent plasma (Fig. 5).

## Discussion

The CONCOR-1 trial found that the use of convalescent plasma for the treatment of hospitalized patients with COVID-19 did not reduce the risk of intubation or death at 30 d. Patients in the convalescent plasma arm experienced more serious adverse events. Convalescent plasma was not associated with an improvement in any of the secondary efficacy outcomes or in any of the subgroup analyses. These results are consistent with the RECOVERY trial and a recent Cochrane meta-analysis^8^. A major additional contribution of our study comes from the study of immunologic markers, which suggest that the antibody profile significantly modified the effect of convalescent plasma compared to standard of care.

**Extended Data Fig. 9:**
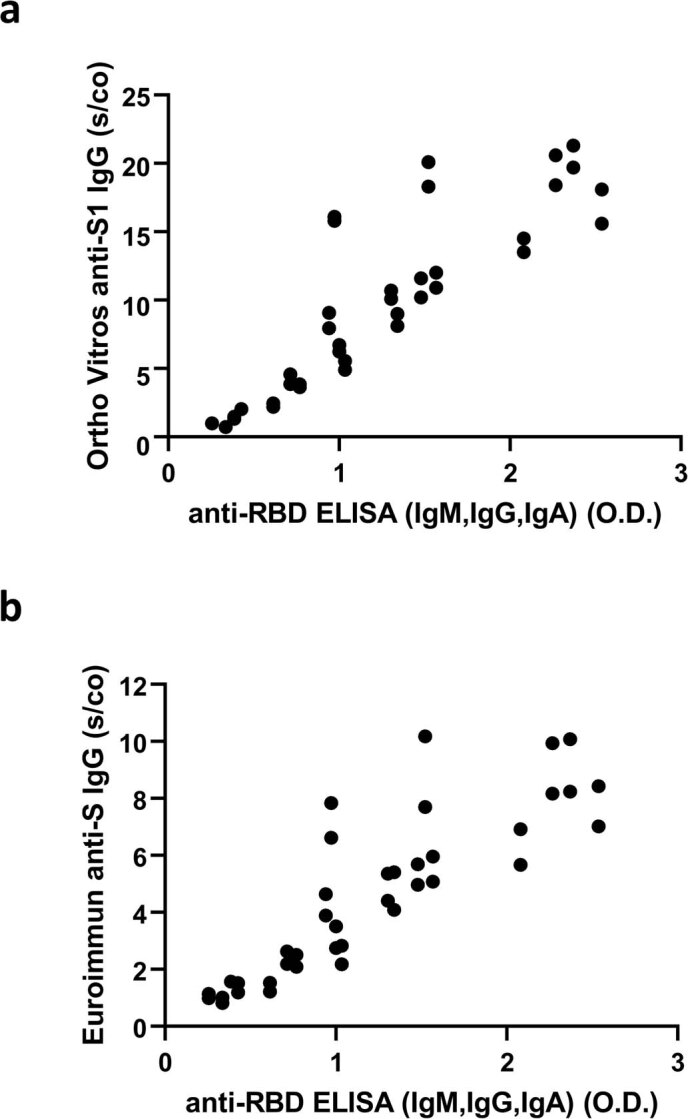
Comparison of in-house ELISA to commercial assays. Values from the Héma-Québec in-house ELISA measuring antibody (IgM, IgA, IgG) binding the receptor binding domain of SARS-CoV-2 Spike protein (used in the current study) are compared to results from Euroimmun (Panel A) and Ortho Vitros (Panel B) commercial assays measuring IgG binding to subunit 1 of the SARS-CoV-2 Spike protein, which contains the receptor binding domain and which were used to qualify convalescent plasma in previous clinical trials. Each sample was tested with the commercial assays twice.

The RECOVERY trial showed that transfusion of high-titer plasma was no better than standard of care in the prevention of key outcomes. The U.S. National Registry report showed that high antibody level plasma was associated with a 34% RR reduction in mortality compared to low antibody level plasma^9^. Our assessment of the role of antibody profile on the clinical effect relative to standard of care is aligned with both of these conclusions. In the RECOVERY trial, plasma with a commercial ELISA cutoff corresponding to a neutralizing antibody titer of 100 or greater was used, and the mortality rate ratio compared to standard of care was 1.00 (95% CI 0.93–1.07). In our trial, plasma from one of the blood suppliers (blood supplier 1) that used a similar antibody threshold (neutralizing antibody titer of 160 of greater) was associated with a similar effect size (OR = 0.95 (95% CI 0.73–1.25)) (Fig. 4). In contrast, the U.S. National Registry study, which lacked a control group, reported that plasma containing high antibody levels (Ortho VITROS IgG anti-spike subunit 1, which contains the RBD, signal-to-cutoff ratio >18.45) was associated with a 34% reduction in mortality compared to plasma containing low antibody levels (signal-to-cutoff ratio <4.62)^9^. In our regression model (Supplementary Table 10), plasma with anti-RBD ELISA values corresponding to this low antibody cutoff (Fig. 4 and Extended Data Figure 9) would have a predicted OR of 1.49 compared to controls (95% CI 0.98–2.29), whereas plasma with the corresponding high antibody cutoff would have a predicted OR of 0.91 (95% CI 0.60–1.40), representing a 38% RR reduction. Thus, the 34% RR reduction observed by the U.S. National Registry^9^ could be explained by increased mortality with low antibody plasma rather than improved mortality with high antibody plasma.

This conclusion is corroborated by the meta-analysis of previous trials based on plasma selection strategy. Although the vast majority of patients included in convalescent plasma trials received high-titer plasma, most patients treated outside of clinical trials did not, including many of those who received plasma according to the current U.S. Food and Drug Administration (FDA) requirements (Ortho VITROS ≥9.5). Only 20% of convalescent plasma included in the U.S. National Registry was considered high-titer^9^. In our study, blood supplier 3 issued the same plasma as the one used in clinical practice as part of the emergency use authorization, and, in our subgroup analysis, convalescent plasma from this blood supplier was associated with worse clinical outcomes (OR = 1.89, 95% CI 1.05–3.43).

The antibody content is critical in determining the potency and potential harm of passive antibody therapy. Convalescent plasma demonstrating high levels of viral neutralization and high levels of Fc-mediated function were independently associated with a reduced risk for intubation or death. The importance of Fc-mediated function is in line with the known functional determinants of the anti-SARS-CoV-2 humoral response. In animal models of COVID-19, mutation of monoclonal antibodies leading to loss of Fc-mediated function, but sparing the neutralizing function, abrogated the protective effect of the antibody^22–25^. In cohort studies of severe COVID-19, low Fc-mediated function, but not neutralization, was associated with mortality^26,27^.

In contrast, high levels of IgG antibodies against the full transmembrane spike protein measured by flow cytometry (which is distinct from commercial assays for IgG against spike subunit 1) were associated with an increased risk of intubation or death after controlling for other antibody markers, suggesting that the transfusion of convalescent plasma containing non-functional anti-SARS-CoV-2 antibodies might be harmful. Antibody Fc-mediated function is dependent on the ability to aggregate and crosslink Fc receptors on target cells. This process can be disrupted by competition from other antibodies with low or absent Fc function^28^. Similar observations were made during HIV vaccine trials, where the development of IgA antibodies against the virus envelope paradoxically increased the risk of infection due to competition with IgG^29,30^, and in animal models of passive immunization where transfer of antibodies could be deleterious to the host^31^.

One positive clinical trial in mild disease (*n* = 160) found that high-titer convalescent plasma administered within 72 h of the onset of mild COVID-19 symptoms improved clinical outcomes compared to placebo in an elderly outpatient population^13^. Furthermore, in a Bayesian re-analysis of the RECOVERY trial, the subgroup of patients who had not yet developed anti-SARS-CoV-2 antibodies appeared to benefit from convalescent plasma^32^. The C3PO trial, which also assessed early treatment with high-titer plasma in high-risk patients, was stopped prematurely for futility after enrolling 511 of 900 planned participants (NCT04355767). In our trial, the median time from the onset of symptoms was 8 d; however, we did not observe a difference in the primary outcome in the subgroup of patients who were randomized within 3 d of diagnosis.

The frequency of serious adverse events was higher in the convalescent plasma group compared to the standard of care group (33.4% versus 26.4%; RR = 1.27, 95% CI 1.02–1.57). Most of these events were caused by worsening hypoxemia and respiratory failure occurring throughout the 30-d follow-up period. This frequency is consistent with the recent Cochrane review that reported an OR of 1.24 (95% CI 0.81–1.90) for serious adverse events^8^. The frequency of transfusion-associated dyspnea and transfusion-associated circulatory overload was 2.1% and 0.8%, respectively, which is similar to other studies of non-convalescent plasma^33^. The rates of transfusion reactions in CONCOR-1 were higher than what were reported in the RECOVERY trial, where transfusion reactions were reported in 13 of 5,795 (0.22%) patients. CONCOR-1 site investigators included many transfusion medicine specialists, and the open-label design might have encouraged reporting. However, the rate of serious transfusion-related adverse events was low (4/614 (0.65%) patients treated with convalescent plasma) and, thus, does not explain the difference in serious adverse events between groups.

CONCOR-1 was a randomized trial designed to examine the effect of convalescent plasma versus standard of care for the primary composite outcome of intubation or death, with a capacity to explore the immunological profile of convalescent plasma and its impact on the effect of convalescent plasma. The trial involved four blood suppliers that provided local convalescent plasma units based on different antibody criteria. As a result, plasma units with a wide distribution of antibody content were included, and comprehensive antibody testing using both quantitative and functional assays provided a detailed description of the plasma product. The open-label design represents a limitation of this study, as knowledge of the treatment group could influence the decision to intubate, report adverse events or administer other treatments. The antibody profile of recipients was unavailable at the time of this analysis. In future work, we will investigate the value of convalescent plasma in patients without a detectable humoral immune response. In addition, other antibody isotypes (IgM and IgA) and IgG subclasses should be evaluated in future studies to determine their effect on clinical outcomes. Additional randomized trials are warranted to assess the early use of high-titer convalescent plasma units in immunocompromised patients with COVID-19 who are unable to mount an efficient anti-SARS-CoV-2 antibody response.

In summary, the CONCOR-1 trial did not demonstrate a difference in the frequency of intubation or death at 30 d with convalescent plasma or standard of care in hospitalized patients with COVID-19 respiratory illness. The antibody content had a significant effect-modifying role for the effect of convalescent plasma on the primary outcome. The lack of benefit and the potential concern of harm caution against the unrestricted use of convalescent plasma for hospitalized patients with COVID-19.

## Methods

### Trial design and oversight

CONCOR-1 was an investigator-initiated, multi-center, open-label, randomized controlled trial conducted at 72 hospital sites in Canada, the United States and Brazil^34^. Eligible patients were randomly assigned to receive either convalescent plasma or standard of care. The study was approved by Clinical Trials Ontario (research ethics board of record: Sunnybrook Health Sciences Centre), project no. 2159; the Quebec Ministry of Health and Social Services multicenter ethics review (research ethics board of record: Comité d’éthique de la recherche du CHU Sainte-Justine), project no. MP-21-2020-2863; the Weil Cornell Medicine General Institutional Review Board, protocol no. 20-04021981; the Comissão Nacional de Ética em Pesquisa, approval no. 4.305.792; the Héma-Québec Research Ethics Board; the Canadian Blood Services Research Ethics Board; Research Ethics BC (research ethics board of record: the University of British Columbia Clinical Research Ethics Board); the Conjoint Health Research Ethics Board; the University of Alberta Health Research Ethics Board (Biomedical Committee); the Saskatchewan Health Authority Research Ethics Board; the University of Saskatchewan Biomedical Research Ethics Board; the University of Manitoba Biomedical Research Board; the Queensway Carleton Hospital Research Ethics Board; the Scarborough Health Network Research Ethics Board; the Windsor Regional Hospital Research Ethics Board; and the Bureau de l’Éthique of Vitalité Health Network. Regulatory authorization was obtained from Health Canada (control no. 238201) and the U.S. FDA (IND 22075). The trial was registered at ClinicalTrials.gov (NCT04348656). An independent data safety monitoring committee performed trial oversight and made recommendations after review of safety reports planned at every 100 patients and at the planned interim analysis based on the first 600 patients. External monitoring was performed at all sites to assess protocol adherence, reporting of adverse events and accuracy of data entry. Full details of the study design, conduct, oversight and analyses are provided in the protocol and statistical analysis plan, which are available online.

### Participants

Eligible participants were (1) ≥16 years of age in Canada or ≥18 years of age in the United States and Brazil; (2) admitted to the hospital ward with confirmed COVID-19; (3) required supplemental oxygen; and (4) a 500-ml of ABO-compatible COVID-19 convalescent plasma (CCP) was available. The availability of ABO-compatible convalescent plasma from donors who had recovered from COVID-19 infection was an eligibility requirement. Exclusion criteria were (1) more than 12 d from the onset of respiratory symptoms; (2) imminent or current intubation; (3) a contraindication to plasma transfusion; or (4) a plan for no active treatment. Consent was obtained from all donors and participants (or their legally authorized representative).

### Randomization and intervention

Patients were randomized in a 2:1 ratio to receive convalescent plasma or standard of care using a secure, concealed, computer-generated, web-accessed randomization sequence (REDCap v11.0.1)^35^. Randomization was stratified by site and age (<60 and ≥60 years) with allocation made with permuted blocks of size 3 or 6. Patients randomized to convalescent plasma received one or two units of apheresis plasma amounting to approximately 500 ml from one or two donors. The plasma was stored frozen and was thawed as per standard blood bank procedures and infused within 24 h of randomization. Patients were monitored by clinical staff for transfusion-related adverse events as per local procedures. Individuals assigned to standard of care received usual medical care as per routine practices at each site. The investigational product was prepared by Canadian Blood Services and Héma-Québec (Canada), the New York Blood Center (United States)^36^ and Hemorio (Brazil). Each supplier had different criteria for qualifying convalescent plasma units that were based on the presence of either viral neutralizing antibodies, measured by the plaque reduction neutralization assay and expressed as the concentration of serum that reduced the number of virus-induced plaques by 50% (PRNT50)^37,38^ using a threshold titer of >1:160 or antibodies against the RBD of the SARS-CoV-2 spike protein using a threshold titer of >1:100. Female donors with previous pregnancies were excluded from donation, unless they tested negative for HLA antibodies. In addition, a retained sample from every plasma donation was tested at reference laboratories after the transfusion for (1) anti-RBD antibodies (IgM, IgA and IgG) by ELISA;^39,40^ (2) viral neutralization by the PRNT50 assay using live virus;^37,38^ (3) IgG antibodies binding to the full-length trimeric transmembrane SARS-CoV-2 spike protein expressed on 293T cells by flow cytometry;^41^ and (4) Fc-mediated function by ADCC assay against the full spike protein expressed on CEM.NKr cells (see supplement for complete description)^42,43^. For each plasma unit, the absolute antibody content was defined as the product of the unit volume and the concentration of the antibody (or functional capacity) in the plasma. These calculations were used to estimate the total antibody content from the transfusion of two units.

### Trial outcomes

The primary outcome was the composite of intubation or death by day 30. Secondary outcomes were: time to intubation or death; ventilator-free days by day 30; in-hospital death by day 90; time to in-hospital death; death by day 30; length of stay in critical care and hospital; need for extracorporeal membrane oxygenation; need for renal replacement therapy; convalescent plasma-associated adverse events; and occurrence of ≥3 grade adverse events by day 30 (classification of adverse events was performed using MedDRA (https://www.meddra.org/) and was graded by Common Terminology Criteria for Adverse Events, v4.03). All transfusion-related adverse events were classified and graded by International Society for Blood Transfusion (www.isbtweb.org) definitions. All patients were followed to day 30, including a 30-d telephone visit for patients who were discharged from hospital. Patients who were in hospital beyond day 30 were followed until discharge for the purpose of determining in-hospital mortality up to day 90.

### Statistical analysis

The primary analysis was based on the intention-to-treat population, which included all individuals who were randomized and for whom primary outcome data were available. The per-protocol population was comprised of eligible patients who were treated according to the randomized allocation of the intervention and received two units (or equivalent) of convalescent plasma within 24 h of randomization.

The effect of convalescent plasma on the composite primary outcome of intubation or death by day 30 was assessed by testing the null hypothesis that the composite event rate was the same under convalescent plasma and standard of care. The RR for the primary outcome (convalescent plasma versus standard of care) was computed with a 95% CI. Secondary outcomes were analyzed as described in the statistical analysis plan (Appendix B in the (Supplementary Information). No multiplicity adjustments were implemented for the secondary analyses. Procedures planned for addressing missing data and subgroup analyses are described in the statistical analysis plan. Forest plots were used to display point estimates, and CIs across subgroups with interaction tests were used to assess effect modification.

The effect-modifying role of antibody content on the primary outcome was assessed via logistic regression controlling for the blood supplier, treatment and the antibody marker. Antibody markers were log-transformed, centered and then divided by the corresponding standard deviation before being entered into logistic regression models (see statistical analysis plan, Appendix B in the Supplementary Information). A multivariate logistic regression model was then fitted adjusting for all four markers. Generalized additive models were used to examine the joint effect of each pair of serologic markers on the primary outcome^44^.

The results from CONCOR-1 were subsequently included in a meta-analysis based on the 20 May 2021 update of the Cochrane systematic review^8^ and known randomized trials published since comparing convalescent plasma to placebo or standard care in patients with COVID-19. These were divided based on whether they used plasma with high antibody titer or not. For each trial, we compared the observed number of deaths at 30 d (or closest available time point before a crossover, if applicable) of patients allocated to convalescent plasma or the control group. Summary estimates for RR with 95% CI were calculated using random effects meta-analysis to account for variation in effect size among studies. Heterogeneity was quantified using inconsistency index (*I*^2^) and *P* values from the chi-square test for homogeneity.

With a 2:1 randomization ratio, 1,200 patients (800 in the convalescent plasma group and 400 in the standard of care group) were needed to provide 80% power to detect an RR reduction of 25% with convalescent plasma for the primary outcome with a 30% event rate under standard of care, based on a two-sided test at the 5% significance level. An interim analysis by a biostatistician unblinded to the allocation of the intervention was planned for when the primary outcome was available for 50% of the target sample. An O’Brien–Fleming stopping rule was employed^45^ to control the overall type I error rate at 5%. Conditional power was used to guide futility decisions with the nominal threshold of 20% to justify early stopping.

### Reporting Summary

Further information on research design is available in the Nature Research Reporting Summary linked to this article.

## Online content

Any methods, additional references, Nature Research reporting summaries, source data, extended data, supplementary information, acknowledgements, peer review information; details of author contributions and competing interests; and statements of data and code availability are available at 10.1038/s41591-021-01488-2.

## Supplementary information

Supplementary Information List of CONCOR-1 investigators, Supplementary Methods, Supplementary Tables 1–10, Study Protocol and Statistical Analysis Plan.

Reporting Summary

## Data Availability

De-identified individual patient data with the data dictionary that underlie the reported results will be made available upon request if the intended use is concordant with existing research ethics board approvals (requests will be reviewed by the CONCOR-1 Steering Committee within 3 months). Proposals for access should be sent to arnold@mcmaster.ca. The protocol and statistical analysis plan are available in the online supplement.

## Code availability

Data were collected with RedCAP v11.0.1 (ref. ^35^). Statistical analyses were conducted in SAS v9.4 and R v4.0.2 using the mgcv package v1.8–36 (refs. ^46,47^).

## References

[1] Seow J (2020). Longitudinal observation and decline of neutralizing antibody responses in the three months following SARS-CoV-2 infection in humans. Nat. Microbiol..

[2] Devasenapathy N (2020). Efficacy and safety of convalescent plasma for severe COVID-19 based on evidence in other severe respiratory viral infections: a systematic review and meta-analysis. CMAJ.

[3] Wood EM, Estcourt LJ, McQuilten ZK (2021). How should we use convalescent plasma therapies for the management of COVID-19?. Blood.

[4] Blackall D (2020). Rapid establishment of a COVID-19 convalescent plasma program in a regional health care delivery network. Transfusion.

[5] Simonovich VA (2021). A randomized trial of convalescent plasma in Covid-19 severe pneumonia. N. Engl. J. Med..

[6] Agarwal A (2020). Convalescent plasma in the management of moderate covid-19 in adults in India: open label phase II multicentre randomised controlled trial (PLACID Trial). Brit. Med. J..

[7] Li L (2020). Effect of convalescent plasma therapy on time to clinical improvement in patients with severe and life-threatening COVID-19: a randomized clinical trial. JAMA.

[8] Piechotta V (2021). Convalescent plasma or hyperimmune immunoglobulin for people with COVID-19: a living systematic review. Cochrane Database Syst. Rev..

[9] Joyner MJ (2021). Convalescent plasma antibody levels and the risk of death from Covid-19. N. Engl. J. Med..

[10] The RECOVERY Collaborative Group. Convalescent plasma in patients admitted to hospital with COVID-19 (RECOVERY): a randomised controlled, open-label, platform trial. *Lancet* **397**, 2049–2059 (2021). 10.1016/S0140-6736(21)00897-7 PMC8121538 34000257

[11] Ray, Y. et al. Clinical and immunological benefits of convalescent plasma therapy in severe COVID-19: insights from a single center open label randomised control trial. Preprint at https://www.medrxiv.org/content/10.1101/2020.11.25.20237883v1 (2020).

[12] Gharbharan A (2021). Effects of potent neutralizing antibodies from convalescent plasma in patients hospitalized for severe SARS-CoV-2 infection. Nat. Commun..

[13] Libster R (2021). Early high-titer plasma therapy to prevent severe Covid-19 in older adults. N. Engl. J. Med..

[14] O’Donnell, M. R. et al. A randomized double-blind controlled trial of convalescent plasma in adults with severe COVID-19. *J. Clin. Invest*. **131**, e150646 (2021). 10.1172/JCI150646 PMC8245169 33974559

[15] Avendaño-Solà, C. et al. Convalescent plasma for COVID-19: a multicenter, randomized clinical trial. Preprint at https://www.medrxiv.org/content/10.1101/2020.08.26.20182444v3 (2020).

[16] Estcourt, L. J. Convalescent plasma in critically ill patients with Covid-19. Preprint at https://www.medrxiv.org/content/10.1101/2021.06.11.21258760v1 (2021).

[17] Bennett-Guerrero, E. et al. Severe acute respiratory syndrome coronavirus 2 convalescent plasma versus standard plasma in Coronavirus Disease 2019 infected hospitalized patients in New York: a double-blind randomized trial. *Crit. Care Med*. **49**, 1015–1025 (2021). 10.1097/CCM.0000000000005066 PMC9658886 33870923

[18] Körper, S. et al. High dose convalescent plasma in COVID-19: results from the randomized trial CAPSID. Preprint at https://www.medrxiv.org/content/10.1101/2021.05.10.21256192v1 (2021).

[19] AlQahtani M (2021). Randomized controlled trial of convalescent plasma therapy against standard therapy in patients with severe COVID-19 disease. Sci. Rep..

[20] Hamdy Salman O, Ail Mohamed HS (2020). Efficacy and safety of transfusing plasma from COVID-19 survivors to COVID-19 victims with severe illness. A double-blinded controlled preliminary study. Egypt. J. Anaesth..

[21] Bajpai, M. et al. efficacy of convalescent plasma therapy compared to fresh frozen plasma in severely ill COVID-19 patients: a pilot randomized controlled trial. Preprint at https://www.medrxiv.org/content/10.1101/2020.10.25.20219337v1 (2020).

[22] Winkler ES (2021). Human neutralizing antibodies against SARS-CoV-2 require intact Fc effector functions for optimal therapeutic protection. Cell.

[23] Suryadevara N (2021). Neutralizing and protective human monoclonal antibodies recognizing the N-terminal domain of the SARS-CoV-2 spike protein. Cell.

[24] Ullah, I. et al. Live imaging of SARS-CoV-2 infection in mice reveals neutralizing antibodies require Fc function for optimal efficacy. Preprint at https://www.biorxiv.org/content/10.1101/2021.03.22.436337v1.full (2021). 10.1016/j.immuni.2021.08.015 PMC8372518 34453881

[25] Schafer, A. et al. Antibody potency, effector function, and combinations in protection and therapy for SARS-CoV-2 infection in vivo. *J. Exp. Med*. **218**, e20201993 (2021). 10.1084/jem.20201993 PMC7673958 33211088

[26] Brunet-Ratnasingham, E. A. S. et al. Integrated immunovirological profiling validates plasma SARS-CoV-2 RNA as an early predictor of COVID-19 mortality. Preprint at https://www.medrxiv.org/content/10.1101/2021.03.18.21253907v1 (2021). 10.1126/sciadv.abj5629 PMC8626074 34826237

[27] Zohar T (2020). Compromised humoral functional evolution tracks with SARS-CoV-2 mortality. Cell.

[28] Casadevall A, Joyner MJ, Pirofski LA (2021). Neutralizing antibody LY-CoV555 for outpatient Covid-19. N. Engl. J. Med..

[29] Haynes BF (2012). Immune-correlates analysis of an HIV-1 vaccine efficacy trial. N. Engl. J. Med..

[30] Tomaras GD (2013). Vaccine-induced plasma IgA specific for the C1 region of the HIV-1 envelope blocks binding and effector function of IgG. Proc. Natl Acad. Sci. USA.

[31] Taborda CP, Rivera J, Zaragoza O, Casadevall A (2003). More is not necessarily better: prozone-like effects in passive immunization with IgG. J. Immunol..

[32] Hamilton FW, Lee T, Arnold DT, Lilford R, Hemming K (2021). Is convalescent plasma futile in COVID-19? A Bayesian re-analysis of the RECOVERY randomized controlled trial. Int. J. Infect. Dis..

[33] Narick C, Triulzi DJ, Yazer MH (2012). Transfusion-associated circulatory overload after plasma transfusion. Transfusion.

[34] Begin P (2021). Convalescent plasma for adults with acute COVID-19 respiratory illness (CONCOR-1): study protocol for an international, multicentre, randomized, open-label trial. Trials.

[35] Harris PA (2009). Research electronic data capture (REDCap)—a metadata-driven methodology and workflow process for providing translational research informatics support. J. Biomed. Inform..

[36] Budhai A (2020). How did we rapidly implement a convalescent plasma program?. Transfusion.

[37] Abe, K. T. et al. A simple protein-based surrogate neutralization assay for SARS-CoV-2. *JCI Insight*. **5**, e142362 (2020). 10.1172/jci.insight.142362 PMC7566699 32870820

[38] Mendoza EJ, Manguiat K, Wood H, Drebot M (2020). Two detailed plaque assay protocols for the quantification of infectious SARS-CoV-2. Curr. Protoc. Microbiol..

[39] Beaudoin-Bussieres, G. et al. Decline of humoral responses against SARS-CoV-2 spike in convalescent individuals. Preprint at https://www.biorxiv.org/content/10.1101/2020.07.09.194639v1 (2020). 10.1128/mBio.02590-20 PMC7569150 33067385

[40] Perreault J (2020). Waning of SARS-CoV-2 RBD antibodies in longitudinal convalescent plasma samples within 4 months after symptom onset. Blood.

[41] Anand, S. P. et al. High-throughput detection of antibodies targeting the SARS-CoV-2 spike in longitudinal convalescent plasma samples. *Transfusion* **61**, 1377–1382 (2021). 10.1111/trf.16318 PMC8013554 33604922

[42] Tauzin, A. N. M. et al. A single BNT162b2 mRNA dose elicits antibodies with Fc-mediated effector functions and boost pre-existing humoral and T cell responses. Preprint at https://www.biorxiv.org/content/10.1101/2021.03.18.435972v1 (2021).

[43] Anand, S. P. et al. Longitudinal analysis of humoral immunity against SARS-CoV-2 spike in convalescent individuals up to eight months post-symptom onset. *Cell Rep. Med*. **2**, 100290 (2021). 10.1016/j.xcrm.2021.100290 PMC8097665 33969322

[44] Wood, S. N. *Generalized Additive Models: An Introduction with R, Second Edition* (Taylor and Francis, 2017).

[45] O’Brien PC, Fleming TR (1979). A multiple testing procedure for clinical trials. Biometrics.

[46] R Development Core Team. *R: A Language and Environment for Statistical Computing* (R Foundation for Statistical Computing, 2010).

[47] Wood SN (2011). Fast stable restricted maximum likelihood and marginal likelihood estimation of semiparametric generalized linear models. J. R. Stat. Soc. Ser. B (Methodol.).

