# Author Correction: Convalescent plasma for hospitalized patients with COVID-19: an open-label, randomized controlled trial

**DOI:** 10.1038/s41591-021-01667-1

**Published:** 2022-01-12

**Authors:** Philippe Bégin, Jeannie Callum, Erin Jamula, Richard Cook, Nancy M. Heddle, Alan Tinmouth, Michelle P. Zeller, Guillaume Beaudoin-Bussières, Luiz Amorim, Renée Bazin, Kent Cadogan Loftsgard, Richard Carl, Michaël Chassé, Melissa M. Cushing, Nick Daneman, Dana V. Devine, Jeannot Dumaresq, Dean A. Fergusson, Caroline Gabe, Marshall J. Glesby, Na Li, Yang Liu, Allison McGeer, Nancy Robitaille, Bruce S. Sachais, Damon C. Scales, Lisa Schwartz, Nadine Shehata, Alexis F. Turgeon, Heidi Wood, Ryan Zarychanski, Andrés Finzi, the CONCOR-1 Study Group, Danièle Marceau, Andy Huang, Holly Carr, Yulia Lin, Rosemarie Lall, Christopher Graham, Christine Arsenault, Valerie Sales, Davinder Sidhu, Makeda Semret, Caroline Hamm, Eneko Arhanchiague, Ziad Solh, Nadim Srour, Karim Soliman, Colin Yee, Vincent Laroche, Susan Nahirniak, Christina Greenaway, Menaka Pai, Andréanne Côté, Jennifer L. Y. Tsang, Christine Cserti-Gazdewich, Danielle Talbot, Sébastien Poulin, Rodrigo Guimaraes, Moira Rushton-Marovac, Alexandra Langlois, Shuoyan Ning, Andrew Shih, Mélissa Boileau, Harjot Singh, Donna Ledingham, Arjuna Ponnampalam, Matthew Yan, Oksana Prokopchuk-Gauk, André Poirier, Gabriel Girouard, Katerina Pavenski, Olivier Drouin, David Harris, Madeleine Durand, Emily Rimmer, Daniel Ovakim, François Ménard, Glenna Cuccarolo, Julie Carruthers, Kayla Lucier, Valérie Arsenault, Marie-Christine Auclair, Meda Avram, Michael Brassard, Sabrina Cerro, Véronica Martinez, Julie Morin, Marie Saint-Jacques, Maxime Veillette, Chantal Armali, Amie Kron, Dimpy Modi, Joanne Duncan, Pauline Justumus, Melanie St John, Geneviève St-Onge, Milena Hadzi-Tosev, Pierre-Marc Dion, Lawrence McGillivary, Andre Valleteau de Moulliac, Sheila A. Nyman, Stephanie Perilli, Paulette Jean Van Vliet, Shannon Lane, Katerina Pavenski, Rebecca Pereira, Emily Sirotich, Julie Abelson, Saara Greene, Aditi Khandelwal, Swarni Thakar, Sarah Longo, Sai Priya Anand, Mehdi Benlarbi, Catherine Bourassa, Marianne Boutin, Jade Descôteaux-Dinelle, Gabrielle Gendron-Lepage, Guillaume Goyette, Annemarie Laumaea, Halima Medjahed, Jérémie Prévost, Jonathan Richard, Daniel Kaufmann, Elsa Brunet-Ratnasingham, Nicolas Chaumont, Michael Drebot, Alyssia Robinson, Emelissa Mendoza, Kristina Dimitrova, Kathy Manguiat, Clark Phillipson, Michael Chan, David Evans, James Lin, Lucie Boyer, Marc Cloutier, Mathieu Drouin, Éric Ducas, Nathalie Dussault, Marie-Josée Fournier, Patricia Landy, Marie-Ève Nolin, Josée Perreault, Tony Tremblay, Ishac Nazy, Feng Xie, David Liu, Michelle Wong, Gus Silverio, Kristin Walkus, Mikaela Barton, Katherine Haveman, Darlene Mueller, Ashley Scott, Matthew Moher, Gordon Wood, Tracey Roarty, Fiona Auld, Gayle Carney, Virginia Thomson, Rodrigo Onell, Keith Walley, Katie Donohoe, Crystal Brunk, Geraldine Hernandez, Tina Jacobucci, Lynda Lazosky, Puneet Mann, Geeta Raval, Ligia Araujo Zampieri, Mypinder Sekhon, Alissa Wright, Nicola James, Gaby Chang, Roy Chen, Kanwal Deol, Jorell Gantioqui, Elyse Larsen, Namita Ramdin, Margaret Roche, Kristin Rosinski, Lawrence Sham, Michelle Storms, Mark Gillrie, Etienne Mahe, Deepa Suryanarayan, Alejandra Ugarte-Torres, Traci Robinson, Mitchell Gibbs, Julia Hewsgirard, Marnie Holmes, Joanna McCarthy, Meagan Ody, Karen Doucette, Wendy Sligl, Ashlesah Sonpar, Kimberley Robertson, Jeffrey Narayan, Leka Ravindran, Breanne Stewart, Lori Zapernick, Stephen Lee, Eric Sy, Alexander Wong, Karolina Gryzb, Sarah Craddock, Dennaye Fuchs, Danielle Myrah, Sana Sunny, Sheila Rutledge Harding, Siddarth Kogilwaimath, Nancy Hodgson, Dawn Johnson, Simona Meier, Kim Thomson, Amila Heendeniya, Brett Houston, Yoav Kenyan, Sylvain Lother, Kendiss Olafson, Barret Rush, Terry Wuerz, Dayna Solvason, Lisa Albensi, Soumya Alias, Nora Choi, Laura Curtis, Maureen Hutmacher, Hessam Kashani, Debra Lane, Nicole Marten, Tracey Pronyk-Ward, Lisa Rigaux, Rhonda Silva, Quinn Tays, Renuka Naidu, Jane Mathews, Margaret Mai, Victoria Miceli, Liz Molson, Gayathri Radhakrishnan, Linda Schaefer, Michel Haddad, Shannon Landry, Robert Chernish, Rebecca Kruisselbrink, Theresa Liu, Jayna Jeromin, Atif Siddiqui, Carla Girolametto, Kristin Krokoszynski, Cheryl Main, Alison Fox-Robichaud, Bram Rochwerg, Erjona Kruja, Dana Ellingham, Disha Sampat, Ngan Tang, Daniela Leto, Meera Karunakaran, Daniel Ricciuto, Kelly Fusco, Taneera Ghate, Holly Robinson, Ian Ball, Sarah Shalhoub, Marat Slessarev, Michael Silverman, Eni Nano, Tracey Bentall, Eileen Campbell, Jeffery Kinney, Seema Parvathy, Evridiki Fera, Anthony La Delfa, Jeya Nadarajah, Henry Solow, Edeliza Mendoza, Katrina Engel, Diana Monaco, Laura Kononow, Sutharsan Suntharalingam, Mike Fralick, Laveena Munshi, Samia Saeed, Omar Hajjaj, Elaine Hsu, Karim Ali, Erick Duan, George Farjou, Lorraine Jenson, Mary Salib, Lisa Patterson, Swati Anant, Josephine Ding, Jane Jomy, Pavani Das, Anna Geagea, Sarah Ingber, Elliot Owen, Alexandra Lostun, Tashea Albano, Antara Chatterjee, Manuel Giraldo, Jennifer Hickey, Ida Lee, Nea Okada, Nicholas Pasquale, Romina Ponzielli, Mary Rahmat, Shelina Sabur, Maria Schlag, Leonita Aguiar, Ashmina Damani, Suhyoung Hong, Mona Kokabi, Carolyn Perkins, Juthaporn Cowan, Tony Giulivi, Derek MacFadden, Joe Cyr, Amanda Pecarskie, Rebecca Porteous, Priscila Ogawa Vedder, Irene Watpool, Phil Berardi, Laith Bustani, Alison Graver, Akshai Iyengar, Magdalena Kisilewicz, Jake Majewski, Misha Marovac, Ruchi Murthy, Karan Sharma, Marina Walcer, Zain Chagla, Jason Cheung, Erick Duan, France Clarke, Karlo Matic, Manuel Giraldo, Jennifer Hickey, Ida Lee, Nea Okada, Nicholas Pasquale, Romina Ponzielli, Mary Rahmat, Shelina Sabur, Maria Schlag, Travis Carpenter, Kevin Schwartz, Paril Suthar, Aziz Jiwajee, Daniel Lindsay, Aftab Malik, Brandon Tse, Larissa Matukas, Joel Ray, Shirley Bell, Elizabeth Krok, Ray Guo, Susan John, Vishal Joshi, Jessica Keen, Chris Lazongas, Jacqueline Ostro, Kevin Shore, Jianmin Wang, Jincheol Choi, Pujitha Nallapati, Tina Irwin, Victor Wang, Petra Sheldrake, Neill Adhikari, Hannah Wunsch, Jacob Bailey, Harley Meirovich, Connie Colavecchia, Eiad Kahwash, Sachin Sud, Martin Romano, Bryan Coburn, Lorenzo Del Sorbo, John Granton, Shahid Husain, Jacob Pendergrast, Abdu Sharkawy, Liz Wilcox, Samia Saeed, Omar Hajjaj, Maria Kulikova, Sophia Massin, Wendy Kennette, Ian Mazzetti, Krista Naccarato, Grace Park, Alex Pennetti, Corrin Primeau, Cathy Vilag, Yves Lapointe, Anne-Sophie Lemay, Emmanuelle Duceppe, Benjamin Rioux-Massé, Cécile Tremblay, Pascale Arlotto, Claudia Bouchard, Stephanie Matte, Marc Messier-Peet, Charles-Langis Francoeur, François Lauzier, Guillaume Leblanc, David Bellemare, Ève Cloutier, Olivier Costerousse, Émilie Couillard Chénard, Rana Daher, Marjorie Daigle, Stéphanie Grenier, Gabrielle Guilbeault, Marie-Pier Rioux, Maude St-Onge, Antoine Tremblay, Brian Beaudoin, Luc Lanthier, Pierre Larrivée, Pierre-Aurèle Morin, Élaine Carbonneau, Robert Lacasse, Julie Autmizguine, Isabelle Boucoiran, Geneviève Du Pont-Thibodeau, Annie La Haye, Vincent Lague, Karine Léveillé, Caroline Quach-Thanh, Guillaume Émériaud, Philippe Jouvet, Élie Haddad, Camille Turgeon-Provost, Susan Fox, Diaraye Baldé, Lorraine Ménard, Suzanne Morissette, Miriam Schnorr-Meloche, Andrée-Anne Turcotte, Caroline Vallée, Stéphanie Castonguay, Tuyen Nguyen, Natalie Rivest, Marios Roussos, Esther Simoneau, Andreea Belecciu, Marie-Hélène Bouchard, Eric Daviau, Cynthia Martin, Nicole Sabourin, Solange Tremblay, Émilie Gagné, Nancy-Lisa Gagné, Julie Larouche, Vanessa Larouche, Véronick Tremblay, Vicky Tremblay, Pierre Blanchette, David Claveau, Marianne Lamarre, Danielle Tapps, Martin Albert, Anatolie Duca, Jean-Michel Leduc, Jean-Samuel Boudreault-Pedneault, Annie Barsalou, Suzanne Deschênes-Dion, Stéphanie Ibrahim, Stéphanie Ridyard, Julie Rousseau, Stéphane Ahern, Marie-Pier Arsenault, Simon-Frédéric Dufresne, Luigina Mollica, Hang Ting Wang, Soizic Beau, Dominique Beaupré, Marjolaine Dégarie, Iris Delorme, Melissa Farkas, Michel-Olivier Gratton, Arnaud Guertin, Guylaine Jalbert, Mélanie Meilleur, Charles Ratté Labrecque, Élaine Santos, Julie Trinh Lu, Julien Auger, Marie-Claude Lessard, Louay Mardini, Yves Pesant, Laurie Delves, Lisa Delves, Sophie Denault, Sofia Grigorova, Michelle Lambert, Nathalie Langille, Corinne Langlois, Caroline Rock, Yannick Sardin-Laframboise, Patrick Archambault, Joannie Bélanger-Pelletier, Estel Duquet-Deblois, Vanessa Dupuis-Picard, Yannick Hamelin, Samuel Leduc, Mélanie Richard, Marc Fortin, Philippe Gervais, Marie-Ève Boulay, Claudine Ferland, Jakie Guertin, Johane Lepage, Annie Roy, Sarit Assouline, Stephen Caplan, Ling Kong, Christina Canticas, Carley Mayhew, Johanne Ouedraogo, Tévy-Suzy Tep, Gerald Batist, Matthew Cheng, Marina Klein, Nadine Kronfli, Patricia Pelletier, Salman Qureshi, Donald Vinh, Robert Dziarmaga, Hansi Peiris, Karène Proulx-Boucher, Jonathan Roger, Molly-Ann Rothschild, Chung-Yan Yuen, Sapha Barkati, Jean-Pierre Routy, Sondra Sinanan-Pelletier, Rémi LeBlanc, Eve St-Hilaire, Patrick Thibeault, Karine Morin, Gilberte Caissie, Jackie Caissie Collette, Line Daigle, Mélissa Daigle, Bianca Gendron, Nathalie Godin, Angela Lapointe, Gabrielle Moreau, Lola Ouellette-Bernier, Joanne Rockburn, Brigitte Sonier-Ferguson, Christine Wilson, Robert DeSimone, Grant Ellsworth, Rebecca Fry, Noah Goss, Roy Gulick, Carlos Vaamonde, Timothy Wilkin, Celine Arar, Jonathan Berardi, Dennis Chen, Cristina Garcia-Miller, Arthur Goldbach, Lauren Gripp, Danielle Hayden, Kathleen Kane, Jiamin Li, Kinge-Ann Marcelin, Christina Megill, Meredith Nelson, Ailema Paguntalan, Gabriel Raab, Gianna Resso, Roxanne Rosario, Noah Rossen, Shoran Tamura, Ethan Zhao, Cheryl Goss, Young Kim, Eshan Patel, Sonal Paul, Tiffany Romero, Naima ElBadri, Lina Flores, Tricia Sandoval, Shashi Kapadia, Ljiljana Vasovic, Shanna-Kay Griffiths, Daniel Alvarado, Fiona Goudy, Melissa Lewis, Marina Loizou, Rita Louie, Chantale Pambrun, Sylvia Torrance, Steven Drews, Janet McManus, Oriela Cuevas, Wanda Lafresne, Patrizia Ruoso, Christine Shin, Tony Steed, Rachel Ward, Isabelle Allard, Marc Germain, Sébastien Girard, Éric Parent, Claudia-Mireille Pigeon, Maria Esther Lopes, Margarida Pêcego, Natalia Rosario, Carlos Alexandre da Costa Silva, Thais Oliveira, Maria Cristina Lopes, Sheila Mateos, Lucette Hall, Sarai Paradiso, Donna Strauss, Donald M. Arnold

**Affiliations:** 1 Department of Pediatrics, CHU Sainte-Justine, Montreal, Quebec Canada; 2 Department of Medicine, Centre Hospitalier de l’Université de Montréal, Montreal, Quebec Canada; 3 Department of Pathology and Molecular Medicine, Kingston Health Sciences Centre and Queen’s University, Kingston, Ontario Canada; 4 Department of Laboratory Medicine and Molecular Diagnostics, Sunnybrook Health Sciences Centre, Toronto, Ontario Canada; 5 Department of Laboratory Medicine and Pathobiology, University of Toronto, Toronto, Ontario Canada; 6 Canadian Blood Services, Ottawa, Ontario Canada; 7 McMaster Centre for Transfusion Research, McMaster University, Hamilton, Ontario Canada; 8 Department of Statistics and Actuarial Science, University of Waterloo, Waterloo, Ontario Canada; 9 Department of Medicine, McMaster University, Hamilton, Ontario Canada; 10 Department of Medicine, University of Ottawa, Ottawa, Ontario Canada; 11 Ottawa Hospital Centre for Transfusion Research, Ottawa Hospital Research Institute, Ottawa, Ontario Canada; 12 Département de Microbiologie, Infectiologie et Immunologie, Université de Montréal, Montreal, Quebec Canada; 13 CHUM Research Center, Montreal, Quebec Canada; 14 Hemorio, Hospital and Regional Blood Center, Rio de Janeiro, Brazil; 15 Héma-Québec, Medical Affairs and Innovation, Quebec City, Quebec Canada; 16 CONCOR-1 Community Advisory Committee representative, Montreal, Quebec Canada; 17 Patient representative, Montreal, Quebec Canada; 18 Innovation Hub, Centre de Recherche du Centre Hospitalier de l’Université de Montréal, Montreal, Quebec Canada; 19 Transfusion Medicine and Cellular Therapy, New York-Presbyterian, New York, NY USA; 20 Department of Pathology and Laboratory Medicine, Weill Cornell Medicine, New York, NY USA; 21 Department of Medicine, Division of Infectious Diseases, Sunnybrook Health Sciences Centre, University of Toronto, Toronto, Ontario Canada; 22 Canadian Blood Services, Vancouver, British Columbia Canada; 23 Department of Pathology and Laboratory Medicine, University of British Columbia, Vancouver, British Columbia Canada; 24 Département de médecine, CISSS de Chaudière-Appalaches, Lévis, Quebec Canada; 25 Département de microbiologie-infectiologie et d’immunologie, Faculté de Médecine, Université Laval, Quebec City, Quebec Canada; 26 Clinical Epidemiology Program, Ottawa Hospital Research Institute, Ottawa, Ontario Canada; 27 Division of Infectious Diseases, Weill Cornell Medical College, New York, NY USA; 28 Department of Community Health Sciences, University of Calgary, Calgary, Alberta Canada; 29 Department of Computing and Software, McMaster University, Hamilton, Ontario Canada; 30 Department of Microbiology, Sinai Health System, Toronto, Ontario Canada; 31 Department of Laboratory Medicine and Pathobiology and Dalla Lana School of Public Health, University of Toronto, Toronto, Ontario Canada; 32 Héma-Québec, Montreal, Quebec Canada; 33 Division of Hematology and Oncology, Department of Pediatrics, CHU Sainte-Justine, Montreal, Quebec Canada; 34 Department of Pediatrics, Université de Montréal, Montreal, Quebec Canada; 35 New York Blood Center Enterprises, New York, NY USA; 36 Department of Critical Care Medicine, Sunnybrook Health Sciences Centre, Toronto, Ontario Canada; 37 Department of Medicine, Interdepartmental Division of Critical Care Medicine, University of Toronto, Toronto, Ontario Canada; 38 Department of Health Research Methods, Evidence & Impact, Faculty of Health Sciences, McMaster University, Hamilton, Ontario Canada; 39 Departments of Medicine, Laboratory Medicine and Pathobiology, Institute of Health Policy Management and Evaluation, University of Toronto, Toronto, Ontario Canada; 40 Division of Hematology, Mount Sinai Hospital, Toronto, Ontario Canada; 41 Department of Anesthesiology and Critical Care Medicine, Division of Critical Care Medicine, Faculty of Medicine, Université Laval, Quebec City, Quebec Canada; 42 CHU de Québec—Université Laval Research Centre, Population Health and Optimal Health Practices Research Unit, Trauma–Emergency–Critical Care Medicine, Université Laval, Quebec City, Quebec Canada; 43 Zoonotic Diseases and Special Pathogens, National Microbiology Laboratory, Public Health Agency of Canada, Winnipeg, Manitoba Canada; 44 Department of Internal Medicine, Sections of Hematology/Medical Oncology and Critical Care, University of Manitoba, Winnipeg, Manitoba Canada; 45 New York-Presbyterian Brooklyn Methodist Hospital, New York, NY USA; 46 Sunnybrook Health Sciences Centre, Toronto, Ontario Canada; 47 Scarborough Health Network, Scarborough, Ontario Canada; 48 Trillium Health Partners, Mississauga, Ontario Canada; 49 Hôpital du Sacré-Cœur-de-Montréal, Montreal, Quebec Canada; 50 Markham Stouffville Hospital, Markham, Ontario Canada; 51 Foothills Hospital, Peter Lougheed Centre, Rockyview General Hospital, Calgary, Alberta Canada; 52 McGill University Hospital Center, Montreal, Quebec Canada; 53 Windsor Regional Hospital, Windsor, Ontario Canada; 54 North York General Hospital, North York, Ontario Canada; 55 London Health Sciences Centre, London, Ontario Canada; 56 CISSS Montérégie-Centre, Hôpital Charles-Lemoyne, Greenfield Park, Quebec Canada; 57 Lakeridge Health, Oshawa and Ajax, Ontario Canada; 58 Grand River Hospital and St. Mary’s General Hospital, Kitchener, Ontario Canada; 59 University of Alberta, Royal Alexandra Hospital, Edmonton, Alberta Canada; 60 Jewish General Hospital, Montreal, Quebec Canada; 61 Hamilton General Hospital, Hamilton, Ontario Canada; 62 Institut de Cardiologie et Pneumologie de Québec, Université Laval, Quebec City, Quebec Canada; 63 Niagara Health System, St. Catharines Site, St. Catharines, Ontario Canada; 64 University Health Network, Toronto, Ontario Canada; 65 Hôpital Cité-de-la-Santé de Laval, Laval, Quebec Canada; 66 Hôpital régional de St-Jérôme, St-Jérôme, Quebec Canada; 67 Queensway Carleton Hospital, Ottawa, Ontario Canada; 68 CHU de Sherbrooke, Sherbrooke, Quebec Canada; 69 St. Joseph’s Healthcare, Hamilton, Ontario Canada; 70 Vancouver General Hospital, Vancouver, British Columbia Canada; 71 Hôpital Maisonneuve-Rosemont, Montreal, Quebec Canada; 72 New York-Presbyterian Lower Manhattan Hospital, New York, NY USA; 73 Pasqua Hospital, Regina General Hospital, Regina, Saskatchewan Canada; 74 Grace General Hospital, Health Sciences Centre, St. Boniface General Hospital, Winnipeg, Manitoba Canada; 75 Abbotsford Regional Hospital, Abbotsford, British Columbia Canada; 76 Royal University Hospital, St. Paul’s Hospital, Saskatoon, Saskatchewan Canada; 77 Hôpital de Trois-Rivières, Trois-Rivières, Quebec Canada; 78 Vitalité Health Network, Moncton, New Brunswick Canada; 79 St. Joseph’s Health Centre, Toronto, Ontario Canada; 80 St. Paul’s Hospital, Vancouver, British Columbia Canada; 81 Royal Jubilee Hospital & Victoria General Hospital, Victoria, British Columbia Canada; 82 Hôpital de Chicoutimi, Chicoutimi, Quebec Canada; 83 Bluewater Health, Sarnia, Ontario Canada; 84 University of Toronto Quality in Utilization, Education and Safety in Transfusion (QUEST) Research Program, Sunnybrook Health Sciences Centre, Toronto, Ontario Canada; 85 Medical Microbiology & Immunology, University of Alberta, Edmonton, Alberta Canada; 86 Juravinski Hospital, Hamilton, Ontario Canada

**Keywords:** Randomized controlled trials, Antibodies, Viral infection, Antibody therapy

Correction to: *Nature Medicine* 10.1038/s41591-021-01488-2, published online 9 September 2021.

In the version of this Article initially published, there was an omission in the member list for the CONCOR-1 Study Group. Valérie Arsenault (Héma-Québec, Montreal, Quebec, Canada) has now been included in the CONCOR-1 Study Group in the online version of the article.

