# Supplementary material for: Convalescent plasma for hospitalized patients with COVID-19: an open-label, randomized controlled trial
Source: Nat Med. 2021 Sep 9;27(11):2012–24. doi: 10.1038/s41591-021-01488-2 (PMC8604729; doi:10.1038/s41591-021-01488-2)
Supplement: Supplementary file 1 — List of CONCOR-1 investigators, Supplementary Methods, Supplementary Tables 1–10, Study Protocol and Statistical Analysis Plan. [file 41591_2021_1488_MOESM1_ESM.pdf]

nature portfolio

https://doi.org/10.1038/s41591-021-01488-2

Supplementary information

Convalescent plasma for hospitalized patients with COVID-19: an open-label, randomized controlled trial

In the format provided by the authors and unedited

CONCOR-1 Supplementary Information

List of CONCOR-1 Investigators 2
Supplemental methods 7
Definitions 7
Inclusion/Exclusion Criteria 8
Study outcomes 9
Electronic Case Report Form 10
Donor recruitment 11
Antibody assays 13
Anti-RBD ELISA 13
Cell surface staining 13
Antibody-dependent cellular cytotoxicity (ADCC) assay 13
Plaque reduction neutralization test (PRNT) assay 13
Supplementary tables 15
Supplementary Table 1: Reasons for exclusion from the intention to treat and per protocol populations. 15
Supplementary Table 2: Baseline characteristics of the intention to treat population. 16
Supplementary Table 3: Baseline characteristics of the per protocol population. 19
Supplementary Table 4: Primary and secondary end-points for the per protocol population. 22
Supplementary Table 5: Adverse events at day 30 for the intention to treat population. 23
Supplementary Table 6: Adverse events at day 30 for the per protocol population. 25
Supplementary Table 7: Convalescent plasma related adverse events at day 30 for the intention to treat population. 27
Supplementary Table 8: Convalescent plasma related adverse events at day 30 for the per protocol population. 28
Supplementary Table 9: Antibody test results overall and by participating blood supplier. 29
Supplementary Table 10: Summary of logistic regression models with standardized continuous log marker dose. 30

Appendix A – Study protocol

Appendix B – Statistical analysis plan

List of CONCOR-1 Investigators

Principal investigators: Donald Arnold MD, Philippe Bégin MD PhD, Jeannie Callum MD

Writing Committee: Philippe Bégin, Jeannie Callum, Erin Jamula, Richard Cook, Nancy M. Heddle, Alan Tinmouth, Michelle P. Zeller, Guillaume Beaudoin-Bussières, Luiz Amorim, Renée Bazin, Kent Cadogan Loftsgard, Richard Carl, Michaël Chassé, Melissa M. Cushing, Nick Daneman, Dana V. Devine, Jeannot Dumaresq, Dean A. Fergusson, Caroline Gabe, Marshall J. Glesby, Na Li, Yang Liu, Allison McGeer, Nancy Robitaille, Bruce S. Sachais, Damon C. Scales, Lisa Schwartz, Nadine Shehata, Alexis F. Turgeon, Heidi Wood, Ryan Zarychanski, Andrés Finzi and Donald M. Arnold.

Steering Committee: Luiz Amorim MD, Renée Bazin PhD, Richard Carl, Michaël Chassé MD PhD, Richard Cook PhD, Melissa M. Cushing MD, Nick Daneman MD, Dana V. Devine PhD, Dean A. Ferguson PhD, Nancy Heddle MSc, Kent Cadogan Loftsgard, Alison McGeer MD, Nancy Robitaille MD, Bruce S. Sachais MD PhD, Damon C. Scales MD PhD, Lisa Schwartz PhD, Nadine Shehata MD, Alan Tinmouth MD, Alexis F. Turgeon MD MSc, Ryan Zarychanski MD MSc, Michelle Zeller MD

Central Methods Centre: Julie Carruthers, Erin Jamula, Kayla Lucier; McMaster Centre for Transfusion Research

Québec Study Coordination: Marie-Christine Auclair, Meda Avram, Michael Brassard, Sabrina Cerro, Véronica Martinez, Julie Morin, Marie Saint-Jacques, Maxime Veillette

Logistics Methods Centre: Chantal Armali, Amie Kron, Dimpy Modi; University of Toronto Quality in Utilization, Education and Safety in Transfusion (QUEST) Research Program, Sunnybrook Health Sciences Centre

Database Design and Management: Joanne Duncan, Pauline Justumus, Melanie St John, Geneviève St-Onge, Milena Hadzi-Tosev

Study Monitors: Jackie Amaral, Tanja Cerovina, Mila Khanna, Monika Wiseman, Karlee Trafford, Samantha Libfeld, Tamara Bright, Louise Rousseau, Rocco Paniccia, Sergio Assis , Daniele Aguiar; Ozmosis Research Inc.

Community Advisory Committee: Pierre-Marc Dion, Kent Cadogan Loftsgard*, Lawrence McGillivary, Andre Valleteau de Moulliac, Sheila A. Nyman, Stephanie Perilli, Paulette Jean Van Vliet , Nancy Heddle, Shannon Lane, Katerina Pavenski, Rebecca Pereira, Emily Sirotich (Advisors: Julie Abelson, Saara Greene, Lisa Schwartz)
*referred to CONCOR-1 research partnership recruitment by the coordinators of the Strategy for Patient- Oriented Research – Primary and Integrated Health Care Innovations Network. - https://spor-pihci.com

Communications Committee: Michelle Zeller (Chair), Kayla Lucier, Aditi Khandelwal, Adrienne Silver, Dana Ellingham, Joanne Duncan, Caroline Gabe, Anne Trueman, Laurent Paul Ménard, Chantal Armali, Florence Meney, Menaka Pai, Julie Carruthers

Inventory and Distribution Committee: Alan Tinmouth (Chair)**,** Michelle Zeller, Melanie St. John, Swarni Thakar, Sarah Longo

Antibody-dependent cellular cytotoxicity assay, flow cytometry, cytokines and viral load: Centre Hospitalier de l'Université de Montréal Research Center: Guillaume Beaudoin-Bussières, Sai Priya Anand, Mehdi Benlarbi, Catherine Bourassa, Marianne Boutin, Jade Descôteaux-Dinelle, Gabrielle Gendron-Lepage, Guillaume Goyette, Annemarie Laumaea, Halima Medjahed, Jérémie Prévost, Jonathan Richard, Daniel Kaufman, Elsa Brunet-Ratnasingham, Nicolas Chaumont, Andrés Finzi.

Virus neutralization assay: Zoonotic Diseases and Special Pathogens, National Microbiology Laboratory, Public Health Agency of Canada, Winnipeg, Manitoba: Michael Drebot, Heidi Wood, Alyssia Robinson, Emelissa Mendoza, Kristina Dimitrova, Kathy Manguiat, Clark Phillipson, Michael Chan**;** Medical Microbiology & Immunology, University of Alberta, Edmonton, Alberta: David Evans, James Lin.

Anti-RBD antibody: Héma-Québec : Lucie Boyer, Marc Cloutier, Mathieu Drouin, Éric Ducas, Nathalie Dussault, Marie-Josée Fournier, Patricia Landy, Marie-Ève Nolin, Josée Perreault, Tony Tremblay.

Health Economics Expertise: Feng Xie PhD

Participating Sites:

British Columbia
Abbotsford Regional Hospital, Abbotsford, British Columbia: Matthew Yan (Principal Investigator), David Liu, Michelle Wong, Gus Silverio, Kristin Walkus, Mikaela Barton, Katherine Haveman, Darlene Mueller, Ashley Scott

Royal Jubilee Hospital & Victoria General Hospital, Victoria, British Columbia: Daniel Ovakim (Principal Investigator), Matthew Moher, Gordon Wood, Tracey Roarty, Fiona Auld, Gayle Carney, Virginia Thomson

St. Paul's Hospital, Vancouver, British Columbia: David Harris (Principal Investigator), Rodrigo Onell, Keith Walley, Katie Donohoe, Crystal Brunk, Geraldine Hernandez, Tina Jacobucci, Lynda Lazosky, Puneet Mann, Geeta Raval, Ligia Araujo Zampieri

Vancouver General Hospital, Vancouver, British Columbia: Andrew Shih (Principal Investigator), Mypinder Sekhon , Alissa Wright, Nicola James, Gaby Chang, Roy Chen, Kanwal Deol, Jorell Gantioqui, Elyse Larsen, Namita Ramdin, Margaret Roche, Kristin Rosinski, Lawrence Sham, Michelle Storms

Alberta
Foothills Hospital, Peter Lougheed Centre, Rockyview General Hospital, Calgary, Alberta: Davinder Sidhu (Principal Investigator), Mark Gillrie, Etienne Mahe, Deepa Suryanarayan, Alejandra Ugarte-Torres, Traci Robinson, Mitchell Gibbs, Julia Hewsgirard, Marnie Holmes, Joanna McCarthy, Meagan Ody

University of Alberta, Royal Alexandra Hospital, Edmonton, Alberta: Susan Nahirniak (Principal Investigator), Karen Doucette, Wendy Sligl, Ashlesah Sonpar, Kimberley Robertson, Jeffrey Narayan, Leka Ravindran, Breanne Stewart, Lori Zapernick

Saskatchewan
Pasqua Hospital, Regina General Hospital, Regina, Saskatchewan: Donna Ledingham (Principal Investigator), Stephen Lee, Eric Sy, Alexander Wong, Karolina Gryzb, Sarah Craddock, Dennaye Fuchs, Danielle Myrah, Sana Sunny

Royal University Hospital, St. Paul's Hospital, Saskatoon, Saskatchewan: Oksana Prokopchuk-Gauk (Principal Investigator), Sheila Rutledge Harding, Siddarth Kogilwaimath, Nancy Hodgson, Dawn Johnson, Simona Meier, Kim Thomson

Manitoba
Grace General Hospital, Health Sciences Centre, St. Boniface General Hospital, Winnipeg, Manitoba: Arjuna Ponnampalam (Principal Investigator), Emily Rimmer (Principal Investigator), Amila Heendeniya (Principal Investigator), Brett Houston, Yoav Kenyan, Sylvain Lother, Kendiss Olafson, Barret Rush, Terry Wuerz, Ryan Zarychanski, Dayna Solvason, Lisa Albensi, Soumya Alias, Nora Choi, Laura Curtis, Maureen Hutmacher, Hessam Kashani, Debra Lane, Nicole Marten, Tracey Pronyk-Ward (Canadian Blood Services), Lisa Rigaux, Rhonda Silva, Quinn Tays

Ontario
Bluewater Health, Sarnia, Ontario: Glenna Cuccarolo (Principal Investigator), Renuka Naidu, Jane Mathews, Margaret Mai, Victoria Miceli, Liz Molson, Gayathri Radhakrishnan, Linda Schaefer, Michel Haddad, Shannon Landry

Grand River Hospital and St. Mary's General Hospital, Kitchener, Ontario: Colin Yee (Principal Investigator), Robert Chernish, Rebecca Kruisselbrink, Theresa Liu, Jayna Jeromin, Atif Siddiqui, Carla Girolametto, Kristin Krokoszynski,

Hamilton General Hospital, Hamilton, Ontario: Menaka Pai (Principal Investigator), Daniela Leto, Cheryl Main, Alison Fox-Robichaud, Bram Rochwerg, Michelle Zeller, Erjona Kruja, Dana Ellingham, Erin Jamula, Meera Karunakaran, Shannon Lane, Kayla Lucier, Disha Sampat, Ngan Tang

Juravinski Hospital, Hamilton, Ontario: Michelle Zeller (Principal Investigator), Daniela Leto, Bram Rochwerg, Erjona

Kruja, Dana Ellingham, Erin Jamula, Meera Karunakaran, Shannon Lane, Kayla Lucier, Disha Sampat, Ngan Tang

Lakeridge Health, Oshawa and Ajax, Ontario: Karim Soliman (Principal Investigator), Daniel Ricciuto, Kelly Fusco, Taneera Ghate, Holly Robinson

London Health Sciences Centre, London, Ontario: Ziad Solh (Principal Investigator), Ian Ball, Sarah Shalhoub, Marat Slessarev, Michael Silverman, Eni Nano, Tracey Bentall, Eileen Campbell, Jeffery Kinney, Seema Parvathy, and the Medical Laboratory Technologists at London Health Sciences Centre

Markham Stouffville Hospital, Markham, Ontario: Valerie Sales (Principal Investigator), Evridiki Fera, Anthony La Delfa, Jeya Nadarajah, Henry Solow, Edeliza Mendoza, Katrina Engel, Diana Monaco, Laura Kononow, Sutharsan Suntharalingam

Mount Sinai Hospital, Toronto, Ontario: Nadine Shehata (Principal Investigator), Mike Fralick, Allison McGeer, Laveena Munshi, Samia Saeed, Omar Hajjaj, Elaine Hsu, and the Medical Laboratory Technologists at Mount Sinai Hospital

Niagara Health System, St. Catharines Site, St. Catharines, Ontario: Jennifer LY Tsang (Principal Investigator), Karim Ali, Erick Duan, George Farjou, Lorraine Jenson, Mary Salib, Lisa Patterson, Swati Anant, Josephine Ding, Jane Jomy

North York General Hospital, North York, Ontario: Eneko Arhanchiague (Principal Investigator), Pavani Das, Anna Geagea, Sarah Ingber, Elliot Owen, Alexandra Lostun, Tashea Albano, Antara Chatterjee, Manuel Giraldo, Jennifer Hickey, Ida Lee, Nea Okada, Nicholas Pasquale, Romina Ponzielli, Mary Rahmat, Shelina Sabur, Maria Schlag, Leonita Aguiar, Ashmina Damani, Suhyoung Hong, Mona Kokabi, Carolyn Perkins

The Ottawa Hospital, Ottawa, Ontario: Alan Tinmouth (Principal Investigator), Juthaporn Cowan, Tony Giulivi, Derek MacFadden, Holly Carr (lead RC), Joe Cyr, Amanda Pecarskie, Rebecca Porteous, Priscila Ogawa Vedder, Irene Watpool.

Queensway Carleton Hospital, Ottawa, Ontario: Moira Rushton-Marovac (Principal Investigator), Phil Berardi, Laith Bustani, Alison Graver, Akshai Iyengar, Magdalena Kisilewicz, Jake Majewski, Misha Marovac, Ruchi Murthy, Karan Sharma, Marina Walcer

St. Joseph's Healthcare, Hamilton, Ontario: Shuoyan Ning (Principal Investigator), Zain Chagla, Jason Cheung, Erick Duan, France Clarke, Karlo Matic, Manuel Giraldo, Jennifer Hickey, Ida Lee, Nea Okada, Nicholas Pasquale, Romina Ponzielli, Mary Rahmat, Shelina Sabur, Maria Schlag

St. Joseph's Health Centre, Toronto, Ontario: Katerina Pavenski (Principal Investigator), Travis Carpenter, Kevin Schwartz, Paril Suthar, Aziz Jiwajee, Daniel Lindsay, Aftab Malik, Brandon Tse

St. Michael's Hospital, Toronto, Ontario: Katerina Pavenski (Principal Investigator), Larissa Matukas, Joel Ray, Paril Suthar, Shirley Bell, Aziz Jiwajee, Elizabeth Krok, Daniel Lindsay, Aftab Malik, Brandon Tse

Scarborough Health Network, Scarborough, Ontario: Rosemarie Lall (Principal Investigator), Ray Guo, Susan John, Vishal Joshi, Jessica Keen, Chris Lazongas, Jacqueline Ostro, Kevin Shore, Jianmin Wang, Jincheol Choi, Pujitha Nallapati, Tina Irwin, Victor Wang, Petra Sheldrake

Sunnybrook Health Sciences Centre, Toronto, Ontario: Yulia Lin (Principal Investigator), Neill Adhikari, Jeannie Callum, Nick Daneman, Hannah Wunsch, Amie Kron, Chantal Armali, Jacob Bailey, Harley Meirovich, Dimpy Modi, Connie Colavecchia

Trillium Health Partners, Mississauga, Ontario: Christopher Graham (Principal Investigator), Eiad Kahwash, Sachin Sud, Martin Romano

University Health Network, Toronto, Ontario: Christine Cserti-Gazdewich (Principal Investigator), Bryan Coburn, Lorenzo Del Sorbo, John Granton, Shahid Husain, Jacob Pendergrast, Abdu Sharkawy, Liz Wilcox, Samia Saeed, Chantal Armali, Omar Hajjaj, Maria Kulikova, Sophia Massin

Windsor Regional Hospital, Windsor, Ontario: Caroline Hamm (Principal Investigator), Wendy Kennette, Ian Mazzetti, Krista Naccarato, Grace Park, Alex Pennetti, Corrin Primeau, Cathy Vilag

Québec

Centre Hospitalier de l'Université de Montréal (CHUM), Montréal, Québec: Madeleine Durand (Principal Investigator), Michaël Chassé, Yves Lapointe, Anne-Sophie Lemay, Emmanuelle Duceppe, Benjamin Rioux-Massé, Cécile Tremblay, Pascale Arlotto, Claudia Bouchard, Stephanie Matte, Marc Messier-Peet, COVID-19 Unit Personnel

CHU de Québec-Université Laval, Québec city, Québec: Alexis F. Turgeon (Principal Investigator), Charles-Langis Francoeur, François Lauzier, Vincent Laroche, Guillaume Leblanc, David Bellemare, Ève Cloutier, Olivier Costerousse, Émilie Couillard Chénard, Rana Daher, Marjorie Daigle, Stéphanie Grenier, Gabrielle Guilbeault, Marie-Pier Rioux, Maude St-Onge, Antoine Tremblay

CHU de Sherbrooke, Sherbrooke, Québec: Alexandra Langlois (Principal Investigator), Brian Beaudoin, Luc Lanthier, Pierre Larrivée, Pierre-Aurèle Morin, Élaine Carbonneau, Robert Lacasse

CHU Sainte-Justine, Montréal, Québec: Olivier Drouin (Principal Investigator), Julie Autmizguine, Philippe Bégin, Isabelle Boucoiran, Geneviève Du Pont Thibodeau, Meda Avram, Mary-Ellen French, Annie La Haye, Vincent Lague, Karine Léveillé, Élie Haddad, Caroline Quach-Thanh, Guillaume Émériaud, Philippe Jouvet, Camille Turgeon-Provost, Nancy Robitaille

CISSS Montérégie-Centre, Hôpital Charles-Lemoyne, Greenfield Park, Québec: Nadim Srour (Principal Investigator), Susan Fox, Diaraye Baldé, Lorraine Ménard, Suzanne Morissette, Miriam Schnorr-Meloche, Andrée-Anne Turcotte, Caroline Vallée

Hôpital Cité-de-la-Santé de Laval, Laval, Québec: Danielle Talbot (Principal Investigator), Stéphanie Castonguay, Tuyen Nguyen, Natalie Rivest, Marios Roussos, Esther Simoneau, Andreea Belecciu, Marie-Hélène Bouchard, Eric Daviau and his team, Cynthia Martin, Nicole Sabourin, Solange Tremblay

Hôpital de Chicoutimi, Chicoutimi, Québec: François Ménard (Principal Investigator), Émilie Gagné, Nancy-Lisa Gagné, Julie Larouche, Vanessa Larouche, Véronick Tremblay, Vicky Tremblay

Hôpital de Trois-Rivières, Trois-Rivières, Québec: André Poirier (Principal Investigator), Pierre Blanchette, David Claveau, Marianne Lamarre, Danielle Tapps

Hôpital du Sacré-Cœur-de-Montréal, Montréal, Québec: Christine Arsenault (Principal Investigator), Martin Albert, Anatolie Duca, Jean-Michel Leduc, Annie Barsalou, Suzanne Deschênes-Dion, Stéphanie Ibrahim, Stéphanie Ridyard, Julie Rousseau

Hôpital Maisonneuve-Rosemont, Montréal, Québec: Mélissa Boileau (Principal Investigator), Stéphane Ahern, Marie-Pier Arsenault, Simon-Frédéric Dufresne, Luigina Mollica, Hang Ting Wang, Soizic Beau, Dominique Beaupré, Marjolaine Dégarie, Iris Delorme, Melissa Farkas, Michel-Olivier Gratton, Arnaud Guertin, Guylaine Jalbert, Mélanie Meilleur, Charles Ratté Labrecque, Élaine Santos, Julie Trinh Lu

Hôpital régional de St-Jérôme, St-Jérôme, Québec: Sébastien Poulin (Principal Investigator), Julien Auger, Marie-Claude Lessard, Louay Mardini, Yves Pesant, Laurie Delves, Lisa Delves, Sophie Denault, Sofia Grigorova, Michelle Lambert, Nathalie Langille, Corinne Langlois, Caroline Rock, Yannick Sardin Laframboise

Hôtel-Dieu de Lévis, Lévis, Québec: Jeannot Dumaresq (Principal Investigator), Danièle Marceau, Patrick Archambault, Joannie Bélanger Pelletier, Estel Duquet-Deblois, Vanessa Dupuis-Picard, Yannick Hamelin, Samuel Leduc, Mélanie Richard

Institut de Cardiologie et Pneumologie de Québec – Université Laval, Québec city, Québec: Andréanne Côté (Principal Investigator), Marc Fortin, Philippe Gervais, Vincent Laroche, Marie-Ève Boulay, Claudine Ferland, Jakie Guertin, Johane Lepage, Annie Roy, BB and COVID-19 unit personnel

Jewish General Hospital, Montréal, Québec: Christina Greenaway (Principal Investigator), Sarit Assouline, Stephen Caplan, Ling Kong, Christina Canticas, Carley Mayhew, Johanne Ouedraogo, Tévy-Suzy Tep

McGill University Hospital Center, Montréal, Québec: Makeda Semret (Principal Investigator), Matthew Cheng, Marina Klein, Nadine Kronfli, Patricia Pelletier, Salman Qureshi, Donald Vinh, Robert Dziarmaga, Hansi Peiris, Karène Proulx-Boucher, Jonathan Roger, Molly-Ann Rothschild, Chung-Yan Yuen

Hôtel-Dieu de Lévis, Lévis, Québec: Jeannot Dumaresq (Principal Investigator), Patrick Archambault, Danièle Marceau, Joannie Bélanger Pelletier, Estel Duquet-Deblois, Vanessa Dupuis-Picard, Yannick Hamelin, Samuel Leduc, Mélanie Richard

New Brunswick
Vitalité Health Network, Moncton, New Brunswick: Gabriel Girouard (Principal Investigator), Richard Garceau, Rémi LeBlanc, Eve St-Hilaire, Patrick Thibeault, Karine Morin, Gilberte Caissie, Jackie Caissie Collette, Line Daigle, Mélissa Daigle, Bianca Gendron, Nathalie Godin, Angela Lapointe, Gabrielle Moreau, Lola Ouellette-Bernier, Joanne Rockburn, Brigitte Sonier-Ferguson, Christine Wilson, and the many collaborating nursing staff on the ICU and COVID units.

New York
Weill Cornell Medicine, New York City, New York: Marshall Glesby (Principal Investigator), Melissa M. Cushing, Robert DeSimone, Grant Ellsworth, Rebecca Fry, Noah Goss, Roy Gulick, Carlos Vaamonde, Timothy Wilkin, Celine Arar, Jonathan Berardi, Dennis Chen, Cristina Garcia-Miller, Arthur Goldbach, Lauren Gripp, Danielle Hayden, Kathleen Kane, Jiamin Li, Kinge-Ann Marcelin, Christina Megill, Meredith Nelson, Ailema Paguntalan, Gabriel Raab, Gianna Resso, Roxanne Rosario, Noah Rossen, Shoran Tamura, Ethan Zhao

New York-Presbyterian Brooklyn Methodist Hospital, New York City, New York: Andy Huang (Principal Investigator), Cheryl Goss, Young Kim, Eshan Patel, Sonal Paul, Tiffany Romero, Naima ElBadri, Lina Flores, Tricia Sandoval

New York-Presbyterian Lower Manhattan Hospital, New York City, New York: Harjot Singh (Principal Investigator), Shashi Kapadia, Ljiljana Vasovic, Shanna-Kay Griffiths, Daniel Alvarado, Fiona Goudy, Melissa Lewis, Marina Loizou, Rita Louie

Brazil
Hemorio, Hospital and Regional Blood Center, Rio de Janeiro: Luiz Amorim Filho (Principal Investigator), Rodrigo Guimaraes, Maria Esther Lopes, Margarida Pêcego, Caroline Gabe, Natalia Rosario, Carlos Alexandre da Costa Silva, Thais Oliveira, Maria Cristina Lopes, Sheila Mateos

Blood Suppliers
Canadian Blood Services: Dana Devine, Chantale Pambrun, Sylvia Torrance, Steven Drews, Janet McManus, Oriela Cuevas, Wanda Lafresne, Patrizia Ruoso, Christine Shin, Tony Steed, Rachel Ward, and the many CBS staff who assisted with the collection and provision of COVID-19 convalescent plasma.

Héma-Québec: Isabelle Allard, Renée Bazin, Marc Germain, Sébastien Girard, Éric Parent, Claudia-Mireille Pigeon, Nancy Robitaille.

Hemorio, Hospital and Regional Blood Center, Rio de Janeiro: Luiz Amorim Filho (Principal Investigator), Rodrigo Guimaraes, Maria Esther Lopes, Margarida Pêcego, Caroline Gabe, Natalia Rosario, Carlos Alexandre da Costa Silva, Thais Oliveira, Maria Cristina Lopes, Sheila Mateos

New York Blood Center: Lucette Hall, Sarai Paradiso, Bruce Sachais, Donna Strauss

The authors would like to acknowledge recovered patients that donated their plasma, the staff involved in the recruitment, collection, qualification, and distribution of COVID-19 convalescent plasma and the staff of the COVID-19 wards who made the study possible.

The authors would also like to thank the members of the **Independent Data Safety Monitoring Committee**: Keyvan

Karkouti MD, Robert Fowler MD, Meghan Delaney MD, George Tomlinson PhD, Darryl Davis MD, and Boris Juelg MD.

Supplemental methods

Definitions

Adverse event (AE)
An AE is any untoward medical occurrence that occurs in a patient or clinical investigation subject administered a pharmaceutical product, and which does not necessarily have a causal relationship with the treatment assignment. An AE can therefore be any unfavorable and unintended sign (including abnormal laboratory finding), symptom, or disease temporally associated with the use of an investigational product, whether or not considered related to the product.

Adverse drug reaction (ADR)
An ADR is any noxious and unintended response to an IMP related to any dose. The phrase 'response to an IMP' means that a causal relationship between the IMP and an AE carries at least a reasonable possibility, i.e., the relationship cannot be ruled out.

Other significant AEs
Any marked laboratory abnormalities or any AEs that lead to an intervention, including withdrawal of drug treatment, dose reduction, or significant additional concomitant therapy.

Severity of AEs
The severity of each AE must be assessed by the Investigator and graded based on CTCAE v4.03. A clinical AE NOT identified elsewhere in the grading table will be graded using one of the following categories:

1. **Mild**: Mild symptoms causing no or minimal interference with usual social & functional activities with intervention not indicated

2. **Moderate**: Moderate symptoms causing greater than minimal interference with usual social & functional activities with intervention indicated

3. **Severe**: Severe symptoms causing inability to perform usual social and functional activities with intervention or hospitalization indicated

4. **Life-threatening:** Potentially life-threatening symptoms causing inability to perform basic self-care functions with intervention indicated to prevent permanent impairment, persistent disability, or death.

5. **Death:** The AE resulted in the subject's death.

Serious AE (SAE)
A SAE is generally defined as any untoward medical occurrence that at any dose:

1. Results in death;

2. Is life-threatening; this means that the subject is at risk of death at the time of the event; it does not mean that the event hypothetically might have caused death if it was more severe;

3. Requires hospitalization (overnight or longer) or prolongation of existing hospitalization or invasive procedure;

4. Results in persistent or significant disability or incapacity;

5. Results in congenital anomaly or birth defect;

6. Is not be immediately life-threatening or result in death or hospitalization but may jeopardize the subject or require intervention to prevent one of the above outcomes.

Inclusion/Exclusion Criteria

Inclusion criteria

1. ≥16 years of age (≥18 years of age in the United States, Brazil, and Israel)
2. Admitted to hospital for confirmed COVID-19 respiratory illness
3. Receiving supplemental oxygen
4. 500 mL of ABO compatible CCP is available

Exclusion criteria

1. Onset of signs or symptoms of COVID-19 respiratory illness >12 days prior to randomization (eg. cough, chest pain, dyspnea, or hypoxia)
2. Intubated or plan in place for intubation
3. Plasma is contraindicated (e.g. history of anaphylaxis from transfusion)
4. Decision in place for no active treatment

Study outcomes

Primary outcomes

The primary outcome is a composite of intubation or death at Day 30.

Secondary outcomes

- Time to intubation or death
- Ventilator-free days at day 30
- In-hospital death by Day 90
- Time to in-hospital death
- Death by Day 30
- Length of stay in ICU
- Length of stay in hospital
- Need for ECMO
- Need for renal replacement therapy
- Myocarditis
- Patient-reported outcome at Day 30
- Incremental cost per quality-adjusted life year (QALY)
- CPP transfusion-associated AEs
- Grade 3 and 4 SAEs
- Cumulative incidence of Grade 3 and 4 SAEs (CTCAE)

Electronic Case Report Form

Study data were collected and managed using REDCap electronic data capture tools hosted at McMaster University. REDCap (Research Electronic Data Capture) is a secure, web-based application designed to support data capture for research studies, providing: 1) an intuitive interface for validated data entry; 2) audit trails for tracking data manipulation and export procedures; 3) automated export procedures for seamless data downloads to common statistical packages; and 4) procedures for importing data from external sources.

Donor recruitment

Donor recruitment for Canadian Sites

Recovered COVID-19 patients were identified as potential donors in collaboration with provincial public health services, local health authorities and individual co-investigators involved in the study. Potential donors were also recruited following self-identification on the routine donor questionnaire or through social media. They were contacted by phone and invited to participate in the program as potential donors. After obtaining consent and reviewing donor selection criteria, eligible participants were directed to a Héma-Québec or Canadian Blood Services apheresis collection site in their area to donate.

Criteria for donors:
All donors met the criteria set forth in the Manuals of donor selection criteria in use at Héma-Québec or Canadian Blood Services. In addition, donors required:

- Prior diagnosis of COVID-19 documented by a PCR test at time of infection or by positive antiSARS-CoV-2 serology following infection
- Male donors, or females with no pregnancy history or with negative anti-HLA antibodies
- At least 6 days since last plasma donation
- Provided informed consent
- A complete resolution of symptoms at least 14 days prior to first donation.

Donor recruitment for United States Sites

Recovered COVID-19 patients were recruited through the New York Blood Center and Weill Cornell Medicine in separate protocols. Potential donors could self-refer via websites or be referred by physicians or identified via the medical record system. Only donors with laboratory confirmed history of COVID-19 were screened.

After providing consent and reviewing FDA and CONCOR-1 Protocol 15 January 2021 v8.0 30 NYBC donor eligibility criteria, donors were screened for the presences of SARS-CoV-2 virus in the nasopharynx if screening within 14 days of complete resolution in accordance with current FDA guidance. Those found to be eligible were referred to NYBC for donation.

Criteria for donors:

- Provision of informed consent
- Aged 18 to 70 years. Donors not longer eligible after their 71st birthday.
- Documented molecular diagnosis of SARS-CoV-2 by RT-PCR by nasopharyngeal swab, oropharyngeal swab, or sputum or detection of anti-SARS-CoV-2 IgG in serum.
- Complete resolution of COVID-19 symptoms at least 14 days prior to donation
- Not currently pregnant or pregnant within 6 weeks by self-report
- Male donors, or females with no pregnancy history or with negative anti-HLA antibodies
- Meets blood donor criteria specified by NYBC, which is consistent with FDA regulations.

Donors were allowed to donate every 7 days. Sterile segments of frozen plasma were attached to all units consisting of at least 2 mL of plasma volume. These segments were submitted to the Centre Hospitalier de l'Université de Montréal Research Center for testing of SARS-COV-2 antibody titer and neutralizing antibody titer.

Donor recruitment for Brazil

Recovered COVID-19 patients were identified as potential donors in collaboration with provincial public health services, local health authorities, individual co-investigators involved in the study. Potential donors could also be recruited following self-identification on the routine donor questionnaire or through social media. They were contacted by phone and invited to come to Hemorio in order to undergo a medical examination that will include the collection of blood samples. If they were approved as plasma donors, after obtaining consent, they were invited to make an appointment to

come back and donate plasma, as soon as possible. Criteria for donors: All donors needed to meet the criteria set forth in the Manuals of donor selection criteria in use at Hemorio and in Brazilian specific regulations.

In addition, donors required:

- Aged 18 to 60 years. Donors are no longer eligible after their 61st birthday
- Prior diagnosis of COVID-19 documented by a PCR test at time of infection or by positive anti-SARS-CoV-2 serology following infection
- Male donors, or females with no pregnancy history or with negative anti-HLA antibodies
- At least 7 days since last plasma donation
- Provided informed consent CONCOR-1 Protocol 15 January 2021 v8.0 31
- 7-day interval between each donation, limited to 4 donations over a 2 month period
- A complete resolution of COVID-19 symptoms at least 14 days prior to first donation.

Antibody assays

Anti-RBD ELISA

Recombinant SARS-CoV-2 S RBD proteins (2.5 µg/ml), were prepared in PBS and were adsorbed to plates (100 µl/well; Immulon 2HB, Thermo Scientific) overnight at 4°C. Coated wells were subsequently blocked with blocking buffer (Phosphate-buffered saline [PBS] containing 0.1% Tween20 and 2% BSA) for 1h at room temperature. Wells were then washed four times with washing buffer (PBS containing 0.1% Tween20). CR3022 mAb (50ng/ml final concentration) or plasma from SARS-CoV-2-infected or uninfected donors (1/100 dilution)in blocking buffer) were incubated with the RBD-coated wells for 1h at room temperature. Plates were washed four times with washing buffer followed by incubation with HRP conjugated goat anti-human IgA+IgG+IgM diluted in blocking buffer (Jackson ImmunoResearch Laboratories, Inc.) for 1h at room temperature, followed by four washes. HRP enzyme activity was determined after a 20 minute incubation with 3,3',5,5'-Tetramethylbenzidine (TMB, ESBE Scientific) followed by blocking with H2SO4 1N. Plates were read using a microplate reader (Synergy H1, Bio-Tek) within 30 min after blocking the reaction. The cut-off value for seropositivity, set at 0.250, was calculated using the mean OD + 3 standard deviations of 13 COVID-19 negative plasma samples (collected in 2019, before the outbreak of SARS-CoV-2) plus a 15% inter-assay coefficient of variation. Following implementation of the assay, we used results from the analysis of 94 convalescent plasma samples and 88 negative samples and the determined cut-off value yielded a sensitivity of 98.9% and a specificity of 98.5%.

Cell surface staining

293T and 293T.SARS-CoV-2.Spike cells were mixed at a 1:1 ratio and stained with the anti-RBD monoclonal antibody CR3022 (5 µg/mL) or plasma (1/250 dilution) for 45 minutes at 37°C. Cells were then washed 2 times with PBS before being incubated with secondary antibodies. AlexaFluor-647 conjugated goat anti-human IgA, IgM, IgM+IgG+IgA (1,70 µg/mL; Jackson ImmunoResearch) or AlexaFluor-647 conjugated mouse anti-human IgG (3 µg/mL; Biolegend) were used as secondary antibodies to stain cells for 20 minutes at room temperature. Cells were then washed 2 times with PBS before being fixed in 2 % PBS-formaldehyde solution. The percentage of transduced cells (GFP+ cells) was determined by gating on the living cell population based on viability dye staining (AquaVivid, Thermo Fisher Scientific). Samples were acquired on a LSRII cytometer (BD Biosciences) and data analysis was performed using FlowJo v10.7.1 (Tree Star).

Antibody-dependent cellular cytotoxicity (ADCC) assay

ADCC was measured as described previously. Briefly, parental CEM.NKr CCR5+ cells were mixed at a 1:1 ratio with CEM.NKr.SARS-CoV-2.Spike cells. These cells were stained for viability (AquaVivid; Thermo Fisher Scientific) and cellular dye (cell proliferation dye eFluor670; Thermo Fisher Scientific) to be used as target cells. Overnight rested PBMCs from healthy donors were stained with another cellular marker (cell proliferation dye eFluor450; Thermo Fisher Scientific) and used as effector cells. Stained target and effector cells were mixed at a ratio of 1:10 in 96-well V-bottom plates. Plasma (1/500 dilution) or monoclonal antibodies CR3022 and CV3-13 (1 µg/mL) were added to the appropriate wells (Jennewein et al., bioRxiv 2021). The plates were subsequently centrifuged for 1 min at 300xg, and incubated at 37°C, 5% CO2 for 5 hours before being fixed in a 2% PBS-formaldehyde solution. ADCC activity (ADCC%) was calculated using the formula: [(% of GFP+ cells in Targets plus Effectors)-(% of GFP+ cells in Targets plus Effectors plus plasma/antibody)]/(% of GFP+ cells in Targets) x 100 by gating on transduced live target cells. Plasma from previous experiments and two monoclonal antibodies (CR3022, CV3-13) were used as quality controls and to standardize the ADCC ratio between experiments. All samples were acquired on an LSRII cytometer (BD Biosciences) and data analysis was performed using FlowJo v10.7.1 (Tree Star).

Plaque reduction neutralization test (PRNT) assay

The PRNT assay at NML was performed as previously published. SARS-CoV-2 (Canada/ON_ON-VIDO-01-2/2020, EPI_ISL_42517) stocks were titrated for use in the PRNT. Serological specimens were diluted 2-fold from 1:20 to 1:640 in DMEM supplemented with 2% FBS and incubated with 50 PFU of SARS-CoV-2 at 37°C and 5% CO2 for 1 hour. The sera-virus mixtures were added to 12-well plates containing Vero E6 cells at 100% confluence, followed by incubation at 37°C and 5% CO2 for 1 hour. After adsorption, a liquid overlay composed of 1.5% carboxymethylcellulose

diluted in MEM, supplemented with 4% FBS, L-glutamine, nonessential amino acids, and sodium bicarbonate, was added to each well; the plates were incubated at 37°C and 5% CO_2 for 72 hours. The liquid overlay was removed, and the cells were fixed with 10% neutral-buffered formalin for 1 hour at room temperature. The monolayers were stained with 0.5% crystal violet for 10 minutes and washed with 20% ethanol. Plaques were enumerated and compared with controls. The highest serum dilution resulting in 50% and 90% reduction in plaques compared with controls were defined as the PRNT50 and PRNT90 endpoint titers, respectively.

Supplementary tables

Supplementary Table 1: Reasons for exclusion from the intention to treat and per protocol populations.

	Convalescent Plasma (n, %)	Standard of Care (n, %)
**All randomized patients**	**627**	**313**
Consent withdrawal	2 (0.3)	0 (0.0)
**Baseline study population**	**625**	**313**
Lost to follow-up (data on primary outcome unavailable; these patients were discharged alive before day 30, and could not be contacted on day 30)	11 (1.8)	6 (1.9)
**Intention to treat population**	**614**	**307**
Ineligible for the trial		
Not receiving supplemental oxygen at the time of randomization (on oxygen at screening)	4 (0.6)	4 (1.3)
Any symptoms onset >12 days prior to randomization[a]	1 (0.2)	0 (0.0)
Protocol deviation		
CCP arm received no CCP		
Received regular plasma	1 (0.2)	
Investigator decision	9 (1.4)	
Patient refused	7 (1.1)	
Total volume transfused < 400 ml	11 (1.8)	
CCP transfusion ended > 24 hour post randomization	33 (5.3)	
**Per protocol population**	548 (87.4)	303 (96.8)

[a] For protocol version 5.0 or earlier (post-randomization, patient revised date of onset of symptoms)

Supplementary Table 2: Baseline characteristics of the intention to treat population.

Excluding the 19 patients lost to follow-up at day 30 or withdrawing consent before day 30. Categorical data presented as number (percentage) and continuous variables as mean ± standard deviation and median (interquartile range).

Characteristic	Convalescent Plasma n=614	Standard of Care n=307	Overall n=921
**Age** - yr	67.8±16.0; 69 (58,80)	67.3±14.8; 68 (58,78)	67.6±15.6; 69 (58,79)
≥60 years	433 (70.5)	216 (70.4)	649 (70.5)
**Sex**			
Male	362 (59.0)	183 (59.6)	545 (59.2)
Female	252 (61.0)	124 (60.4)	376 (60.8)
**Pregnant at randomization**	4(0.7)		4(0.4)
**Ethnicity**			
White	300 (48.9)	151 (49.2)	451 (49.0)
Asian	101 (16.4)	44 (14.3)	145 (15.7)
Hispanic or Latino	33 (5.4)	9 (2.9)	42 (4.6)
Black	25 (4.1)	11 (3.6)	36 (3.9)
Other	37 (6.0)	28 (9.1)	65 (7.1)
Unknown	118 (19.2)	64 (20.8)	182 (19.8)
**ABO blood group**			
O	265 (43.2)	112 (36.5)	377 (40.9)
A	231 (37.6)	119 (38.8)	350 (38.0)
B	87 (14.2)	54 (17.6)	141 (15.3)
AB	31 (5.0)	22 (7.2)	53 (5.8)
**BMI – kg/m^2**	30.1±7.5; 29 (25,33)	30.0±7.5; 29 (25,33)	30.1±7.5; 29 (25,33)
BMI <30	248 (40.4)	121 (39.4)	369 (40.1)
BMI ≥30	198 (32.2)	100 (32.6)	298 (32.4)
Unknown	168 (27.4)	86 (28.0)	254 (27.6)
**Smoking status**			
Never Smoking	324 (52.8)	159 (51.8)	483 (52.4)
Former Smoker	188 (30.6)	104 (33.9)	292 (31.7)
Current Smoker	19 (3.1)	6 (2.0)	25 (2.7)

Characteristic	Convalescent Plasma n=614	Standard of Care n=307	Overall n=921
Unknown	83 (13.5)	38 (12.4)	121 (13.1)
**Smoking history**			
<15 years or non-smoker	355 (57.8)	172 (56.0)	527 (57.2)
≥15 years	84 (13.7)	53 (17.3)	137 (14.9)
Smokers with unknown history	92 (15.0)	44 (14.3)	136 (14.8)
Unknown	83 (13.5)	38 (12.4)	121 (13.1)
**Presence of comorbidity**			
Diabetes	214 (34.9)	104 (33.9)	318 (34.5)
Cardiac disease	379 (61.7)	193 (62.9)	572 (62.1)
Baseline respiratory diseases	145 (23.6)	79 (25.7)	224 (24.3)
**Abnormal CT chest or chest x-ray result before randomization**	553 (90.1)	261 (85.0)	814 (88.4)
**Medication for other research study at baseline**			
No	562 (91.5)	267 (87.0)	829 (90.0)
Yes	52 (8.5)	40 (13.0)	92 (10.0)
**Medication for COVID-19 at baseline**			
Azithromycin	277(45.1)	134(43.6)	411(44.6)
Other antibiotics	400(65.1)	182(59.3)	582(63.2)
Steroid/Immunomodulator	489(79.6)	244(79.5)	733(79.6)
Antiviral medications	161(26.2)	78(25.4)	239(26.0)
Anticoagulants	345(56.2)	175(57.0)	520(56.5)
Other COVID-19 Medications	76(12.4)	36(11.7)	112(12.2)
**Medication not for COVID-19 at baseline**			
ACE inhibitor	84(13.7)	62(20.2)	146(15.9)
ACE receptor blocker(ARB)	76(12.4)	46(15.0)	122(13.2)
Non-steroidal anti-inflammatory drugs(NSAIDs)	77(12.5)	51(16.6)	128(13.9)
Colchicine	5(0.8)	2(0.7)	7(0.8)
Systemic corticosteroids	60(9.8)	35(11.4)	95(10.3)
Inhaled corticosteroids	82(13.4)	42(13.7)	124(13.5)

Characteristic	Convalescent Plasma n=614	Standard of Care n=307	Overall n=921
Immunomodulatory agents	22(3.6)	17(5.5)	39(4.2)
Anticoagulants	132(21.5)	63(20.5)	195(21.2)
**Systemic corticosteroids at baseline**	496(80.8)	258(84.0)	754(81.9)
**Oxygen status at baseline (FiO2)**	49.7±25.3; 40 (30,65)	49.1±25.3; 40 (30,60)	49.5±25.3; 40 (30,64)
**Time from first Covid-19 symptom to randomization (days)**	8.0±3.8; 8 (5,10)	7.8±3.4; 8 (5,10)	7.9±3.7; 8 (5,10)
**Time from Covid-19 diagnosis[a] to randomization (days)**	5.0±3.6; 4 (2,8)	5.0±4.4; 4 (2,7)	5.0±3.9; 4 (2,7)
**Location at randomization**			
Ward	494(80.5)	255(83.1)	749(81.3)
ICU	120(19.5)	52(16.9)	172(18.7)
**Enrolled in other clinical trials**	166(27.0)	96(31.3)	262(28.4)

[a]Day of positive Covid-19 test.

Supplementary Table 3: Baseline characteristics of the per protocol population.

Categorical data presented as number (percentage) and continuous variables as mean ± standard deviation and median (interquartile range).

Characteristic	Convalescent Plasma n=548	Standard of Care n=303	Overall n=851
**Age** - yr	67.1±16.1; 68 (57,79)	67.3±14.9; 68 (58,78)	67.2±15.7; 68 (57,79)
<60 years	169(30.8)	90(29.7)	259(30.4)
≥60 years	379(69.2)	213(70.3)	592(69.6)
**Sex**			
Male	323(58.9)	182(60.1)	505(59.3)
Female	225(41.1)	121(39.9)	346(40.7)
**Pregnant at randomization**	4(0.7)		4(0.5)
**Ethnicity**			
White	267(48.7)	150(49.5)	417(49.0)
Asian	94(17.2)	44(14.5)	138(16.2)
Hispanic or Latino	29(5.3)	8(2.6)	37(4.3)
Black	21(3.8)	11(3.6)	32(3.8)
Other	34(6.2)	28(9.2)	62(7.3)
Unknown	103(18.8)	62(20.5)	165(19.4)
**ABO blood group**			
O	236(43.1)	111(36.6)	347(40.8)
A	206(37.6)	117(38.6)	323(38.0)
B	78(14.2)	53(17.5)	131(15.4)
AB	28(5.1)	22(7.3)	50(5.9)
**BMI – kg/m^2**	30.1±7.6; 29 (25,33)	30.0±7.5; 29 (25,33)	30.1±7.5; 29 (25,33)
BMI <30	220(40.1)	121(39.9)	341(40.1)
BMI ≥30	177(32.3)	100(33.0)	277(32.5)
Unknown	151(27.6)	82(27.1)	233(27.4)
**Smoking status**			
Never Smoking	291(53.1)	157(51.8)	448(52.6)
Former Smoker	165(30.1)	103(34.0)	268(31.5)

Characteristic	Convalescent Plasma n=548	Standard of Care n=303	Overall n=851
Current Smoker	16(2.9)	6(2.0)	22(2.6)
Unknown	76(13.9)	37(12.2)	113(13.3)
**Smoking history**			
<15 years or non-smoker	319(58.2)	170(56.1)	489(57.5)
≥15 years	73(13.3)	52(17.2)	125(14.7)
Smokers with unknown history	80(14.6)	44(14.5)	124(14.6)
Unknown	76(13.9)	37(12.2)	113(13.3)
**Presence of comorbidity**			
Diabetes	184(33.6)	104(34.3)	288(33.8)
Cardiac disease	328(59.9)	193(63.7)	521(61.2)
Baseline respiratory diseases	130(23.7)	79(26.1)	209(24.6)
**Abnormal CT chest or chest x-ray result before randomization**	494(90.1)	258(85.1)	752(88.4)
**Medication for other research study at baseline**			
No	504(92.0)	263(86.8)	767(90.1)
Yes	44(8.0)	40(13.2)	84(9.9)
**Medication for COVID-19 at baseline**			
Azithromycin	244(44.5)	132(43.6)	376(44.2)
Other antibiotics	353(64.4)	181(59.7)	534(62.7)
Steroid/Immunomodulator	434(79.2)	240(79.2)	674(79.2)
Antiviral medications	144(26.3)	78(25.7)	222(26.1)
Anticoagulants	304(55.5)	172(56.8)	476(55.9)
Other COVID-19 Medications	69(12.6)	36(11.9)	105(12.3)
**Medication not for COVID-19 at baseline**			
ACE inhibitor	74(13.5)	62(20.5)	136(16.0)
ACE receptor blocker(ARB)	63(11.5)	46(15.2)	109(12.8)
Non-steroidal anti-inflammatory drugs(NSAIDs)	69(12.6)	51(16.8)	120(14.1)
Colchicine	5(0.9)	2(0.7)	7(0.8)
Systemic corticosteroids	54(9.9)	35(11.6)	89(10.5)

Characteristic	Convalescent Plasma n=548	Standard of Care n=303	Overall n=851
Inhaled corticosteroids	76(13.9)	41(13.5)	117(13.7)
Immunomodulatory agents	21(3.8)	17(5.6)	38(4.5)
Anticoagulants	114(20.8)	63(20.8)	177(20.8)
**Systemic corticosteroids at baseline**	441(80.5)	254(83.8)	695(81.7)
**Oxygen status at baseline (FiO_2)**	49.9±25.6; 40 (30,70)	49.1±25.3; 40 (30,60)	49.6±25.5; 40 (30,65)
**Time from any symptom onset to randomization (days)**	8.0±3.8; 8 (5,10)	7.8±3.4; 8 (5,10)	7.9±3.7; 8 (5,10)
**Time from Covid-19 diagnosis[a] to randomization (days)**	5.0±3.6; 4 (2,8)	5.0±4.4; 4 (2,7)	5.0±3.9; 4 (2,7)
**Location at randomization**			
Ward	438(79.9)	251(82.8)	689(81.0)
ICU	110(20.1)	52(17.2)	162(19.0)
**Enrolled in other clinical trials**	143(26.1)	96(31.7)	239(28.1)

[a]Day of positive Covid-19 test.

Supplementary Table 4: Primary and secondary end-points for the per protocol population.

Per protocol analysis	Population	CCP n=548	SOC n=303	Treatment effect[a]
**Intubation or death at day30**	**851**[b]	**167 (30.5)**	**85 (28.1)**	**RR=1.09 (0.87,1.35), p=0.4612**
Time to Intubation or in-hospital death (days) by day30	851			HR=1.062 (0.819,1.376), p=0.6516
Number of ventilation-free days by day30	851	23.9 ±10.2	24.0 ±10.5	MD=-0.1 (-1.5, 1.3), p=0.8520
Any death by day30	851	114 (20.8)	62 (20.5)	RR=1.02 (0.77,1.34), p=0.9065
Length of stay in ICU by day 30	851	4.3 ±8.0	3.7 ±7.1	MD=0.6 (-0.4, 1.7), p=0.2485
Need for Renal replacement therapy by day30[c]	842	7 (1.3)	6 (2.0)	RR=0.64 (0.22,1.89), p=0.4225
Serious Adverse event by day30	851	172 (31.4)	80 (26.4)	RR=1.19 (0.95,1.49), p=0.1321
**Per protocol analysis**		**n=557**	**n=309**	**Treatment effect**
In-hospital death by day 90	866[d]	127 (22.8)	68 (22.0)	RR=1.04 (0.80,1.34), p=0.7890
Time to in-hospital death by day 90	866			HR=0.946 (0.704,1.270), p=0.946
Length of stay in hospital by day 90	866			HR=0.930 (0.810,1.067), p=0.2999

[a] Relative Risk (RR, 95% confidence interval), Hazard Ratio (HR, 95% confidence interval) and Mean Difference (MD, with 95% CI based on robust bootstrap standard errors)

[b]See Figure 1 for exclusions to the per protocol population

[c]Excluding 9 patients on chronic kidney replacement therapy at baseline

[d]Including 15 additional patients above the per-protocol, n=851, who were lost to follow-up after discharge from hospital

Supplementary Table 5: Adverse events at day 30 for the intention to treat population.

Categorical data presented as number (percentage) and continuous variables as mean ± standard deviation and median (interquartile range).

**Intention to treat analysis**	Convalescent Plasma n=614	Standard of Care n=307	Overall n=921
**Patients with adverse events**			
Grade ≥ 3 (Severe)	260(42.3)	109(35.5)	369(40.1)
Grade ≥ 4 (Life-Threatening)	188(30.6)	74(24.1)	262(28.4)
Grade 5 (Fatal)	141 (23.0)	63 (20.5)	204 (22.1)
**Number of adverse events per patient**			
Grade ≥ 3 (Severe)	0.9±1.7; 0 (0,1)	0.6±1.2; 0 (0,1)	0.8±1.5; 0 (0,1)
Grade ≥ 4 (Life-Threatening)	0.4±0.7; 0 (0,1)	0.3±0.7; 0 (0,0)	0.4±0.7; 0 (0,1)
Grade 5 (Fatal)	0.2±0.4; 0 (0,0)	0.2±0.4; 0 (0,0)	0.2±0.4; 0 (0,0)
**Serious adverse event (grade >3)**	205 (33.4)	81 (26.4)	286 (31.1)
**Number of SAEs per patient**	0.4±0.8; 0 (0,1)	0.3±0.7; 0 (0,1)	0.4±0.7; 0 (0,1)
**Grade 3 non CCP related adverse event**			
Hypoxia	23 (3.7)	7 (2.3)	30 (3.3)
Lymphocyte count decreased	17 (2.8)	8 (2.6)	25 (2.7)
Infections and infestations - Other, specify	15 (2.4)	8 (2.6)	23 (2.5)
Anemia	15 (2.4)	3 (1.0)	18 (2.0)
Hyperglycemia	13 (2.1)	5 (1.6)	18 (2.0)
Hypertension	11 (1.8)	2 (0.7)	13 (1.4)
Pneumonitis	10 (1.6)	1 (0.3)	11 (1.2)
Hypermagnesemia	9 (1.5)	1 (0.3)	10 (1.1)
**Grade 4 non CCP related adverse event**			
Hypoxia	22 (3.6)	6 (2.0)	28 (3.0)
Acute kidney injury	12 (2.0)	7 (2.3)	19 (2.1)
Sepsis	8 (1.3)	4 (1.3)	12 (1.3)
**Grade 5 non CCP related adverse event**			
Respiratory failure	63 (10.3)	32 (10.4)	95 (10.3)
Hypoxia	30 (4.9)	15 (4.9)	45 (4.9)

**Intention to treat analysis**	Convalescent Plasma n=614	Standard of Care n=307	Overall n=921
Death[a]	14 (2.3)	4 (1.3)	18 (2.0)
**Grade ≥3 non CCP related adverse event**			
Respiratory failure	76 (12.4)	35 (11.4)	111 (12.1)
Hypoxia	75 (12.2)	28 (9.1)	103 (11.2)
Lymphocyte count decreased	17 (2.8)	8 (2.6)	25 (2.7)
Infections and infestations - Other	16 (2.6)	8 (2.6)	24 (2.6)
Acute kidney injury	13 (2.1)	10 (3.3)	23 (2.5)
Sepsis	14 (2.3)	7 (2.3)	21 (2.3)
Anemia	16 (2.6)	3 (1.0)	19 (2.1)
Death[a]	14 (2.3)	4 (1.3)	18 (2.0)
Hyperglycemia	13 (2.1)	5 (1.6)	18 (2.0)
Adult respiratory distress syndrome	10 (1.6)	3 (1.0)	13 (1.4)
Dyspnea	9 (1.5)	4 (1.3)	13 (1.4)
Hypertension	11 (1.8)	2 (0.7)	13 (1.4)
Hypotension	10 (1.6)	3 (1.0)	13 (1.4)
Respiratory, thoracic, mediastinal disorders	10 (1.6)	3 (1.0)	13 (1.4)
Lung infection	9 (1.5)	4 (1.3)	13 (1.4)
Thromboembolic event	7 (1.1)	5 (1.6)	12 (1.3)
Pneumonitis	10 (1.6)	1 (0.3)	11 (1.2)
Hypermagnesemia	9 (1.5)	1 (0.3)	10 (1.1)

[a]Adverse event classified by the investigator as CTCAE term “death (not otherwise specified)”.

Supplementary Table 6: Adverse events at day 30 for the per protocol population.

Categorical data presented as number (percentage) and continuous variables as mean ± standard deviation and median (interquartile range).

**Per protocol analysis**	Convalescent Plasma n=548	Standard of Care n=303	Overall n=851
**Patients with adverse events**			
Grade ≥ 3 (Severe)	218(39.8)	108(35.6)	326(38.3)
Grade ≥ 4 (Life-Threatening)	156(28.5)	73(24.1)	229(26.9)
Grade ≥ 5 (Fatal)	114 (20.8)	62 (20.5)	176 (20.7)
**Number of adverse events per patient**			
Grade ≥ 3 (Severe)	0.8±1.7; 0 (0,1)	0.6±1.2; 0 (0,1)	0.7±1.5; 0 (0,1)
Grade ≥ 4 (Life-Threatening)	0.4±0.7; 0 (0,1)	0.3±0.7; 0 (0,0)	0.3±0.7; 0 (0,1)
Grade 5 (Fatal)	0.2±0.4; 0 (0, 0)	0.2±0.4; 0 (0, 0)	0.2±0.4; 0 (0, 0)
**Serious adverse event**	172 (31.4)	80 (26.4)	252 (29.6)
**Number of SAEs per patient**	0.4±0.7; 0 (0, 1)	0.3±0.7; 0 (0, 1)	0.4±0.7; 0 (0, 1)
**Grade 3 non CCP related adverse event**			
Hypoxia	20 (3.6)	7 (2.3)	27 (3.2)
Infections and infestations - Other, specify	14 (2.6)	8 (2.6)	22 (2.6)
Lymphocyte count decreased	14 (2.6)	8 (2.6)	22 (2.6)
Anemia	14 (2.6)	3 (1.0)	17 (2.0)
Hyperglycemia	11 (2.0)	5 (1.7)	16 (1.9)
Hypertension	10 (1.8)	2 (0.7)	12 (1.4)
Pneumonitis	10 (1.8)	1 (0.3)	11 (1.3)
Hypermagnesemia	8 (1.5)	1 (0.3)	9 (1.1)
Hypophosphatemia	8 (1.5)	1 (0.3)	9 (1.1)
Urinary tract infection	7 (1.3)	2 (0.7)	9 (1.1)
**Grade 4 non CCP related adverse event**			
Hypoxia	20 (3.6)	6 (2.0)	26 (3.1)
Acute kidney injury	11 (2.0)	7 (2.3)	18 (2.1)
Sepsis	8 (1.5)	4 (1.3)	12 (1.4)

**Per protocol analysis**	Convalescent Plasma n=548	Standard of Care n=303	Overall n=851
**Grade 5 non CCP related adverse event**			
Respiratory failure	53 (9.7)	31 (10.2)	84 (9.9)
Hypoxia	25 (4.6)	15 (5.0)	40 (4.7)
Death[a]	11 (2.0)	4 (1.3)	15 (1.8)
**Grade 3/4/5 non CCP related adverse event**			
Respiratory failure	64 (11.7)	34 (11.2)	98 (11.5)
Hypoxia	65 (11.9)	28 (9.2)	93 (10.9)
Infections and infestations - Other, specify	14 (2.6)	8 (2.6)	22 (2.6)
Lymphocyte count decreased	14 (2.6)	8 (2.6)	22 (2.6)
Acute kidney injury	12 (2.2)	10 (3.3)	22 (2.6)
Sepsis	13 (2.4)	7 (2.3)	20 (2.4)
Anemia	15 (2.7)	3 (1.0)	18 (2.1)
Hyperglycemia	11 (2.0)	5 (1.7)	16 (1.9)
Death[a]	11 (2.0)	4 (1.3)	15 (1.8)
Dyspnea	8 (1.5)	4 (1.3)	12 (1.4)
Hypertension	10 (1.8)	2 (0.7)	12 (1.4)
Respiratory, thoracic and mediastinal disorders	9 (1.6)	3 (1.0)	12 (1.4)
Lung infection	8 (1.5)	4 (1.3)	12 (1.4)
Pneumonitis	10 (1.8)	1 (0.3)	11 (1.3)
Thromboembolic event	6 (1.1)	5 (1.7)	11 (1.3)
Adult respiratory distress syndrome	7 (1.3)	3 (1.0)	10 (1.2)
Hypotension	7 (1.3)	3 (1.0)	10 (1.2)
Hypermagnesemia	8 (1.5)	1 (0.3)	9 (1.1)
Hypophosphatemia	8 (1.5)	1 (0.3)	9 (1.1)
Urinary tract infection	7 (1.3)	2 (0.7)	9 (1.1)

[a]Adverse event classified by the investigator as CTCAE term “death (not otherwise specified)”.

Supplementary Table 7: Convalescent plasma related adverse events at day 30 for the intention to treat population.

**Intention to treat analysis**	**Convalescent Plasma** (n=614)
CCP related adverse event, n	
CTCAE Grade 1	10 (1.6)
CTCAE Grade 2	10 (1.6)
CTCAE Grade 3 (Severe)	8 (1.3)
CTCAE Grade 4 (Life-Threatening)	4 (0.7)
CTCAE Grade 5 (Fatal)	
Number of CCP related AEs per patient*	0.0±0.1; 0 (0, 0)
CTCAE Grade 1	0.0±0.1; 0 (0, 0)
CTCAE Grade 2	0.0±0.1; 0 (0, 0)
CTCAE Grade 3 (Severe)	0.0±0.1; 0 (0, 0)
CTCAE Grade 4 (Life-Threatening)	0.0±0.0; 0 (0, 0)
CTCAE Grade 5 (Fatal)	
CCP related adverse event, n	
ISBT Grade 1	18 (2.9)
ISBT Grade 2	10 (1.6)
ISBT Grade 3	4 (0.7)
ISBT Grade 4	
Number of CCP related AEs per patient[a]	
ISBT Grade 1	0.0±0.2; 0 (0,0)
ISBT Grade 2	0.0±0.1; 0 (0,0)
ISBT Grade 3	0.0±0.1; 0 (0,0)
ISBT Grade 4	0.0±0.0; 0 (0,0)
Event name, n	
TAD	13 (2.1)
Minor Allergic Reaction	9 (1.5)
TACO	5 (0.8)
Febrile Non-Hemolytic Reaction	4 (0.7)
Other (Hypertensive Reaction)	2 (0.3)
Hypotensive Reaction	1 (0.2)
Possible TRALI	1 (0.2)

[a]Mean/standard deviation); median (interquartile range)

Supplementary Table 8: Convalescent plasma related adverse events at day 30 for the per protocol population.

**Per protocol analysis**	**Convalescent Plasma** (n=548)
CCP related adverse event, n	
CTCAE Grade 1	10 (1.8)
CTCAE Grade 2	10 (1.8)
CTCAE Grade 3 (Severe)	4 (0.7)
CTCAE Grade 4 (Life-Threatening)	4 (0.7)
CTCAE Grade 5 (Fatal)	
Number of CCP related AEs per patient*	0.0±0.2; 0 (0, 0)
CTCAE Grade 1	0.0±0.2; 0 (0, 0)
CTCAE Grade 2	0.0±0.1; 0 (0, 0)
CTCAE Grade 3 (Severe)	0.0±0.1; 0 (0, 0)
CTCAE Grade 4 (Life-Threatening)	0.0±0.0; 0 (0, 0)
CTCAE Grade 5 (Fatal)	
CCP related adverse event, n	17 (3.1)
ISBT Grade 1	7 (1.3)
ISBT Grade 2	4 (0.7)
ISBT Grade 3	
ISBT Grade 4	
Number of CCP related AEs per patient[a]	0.0±0.2; 0 (0, 0)
ISBT Grade 1	0.0±0.1; 0 (0, 0)
ISBT Grade 2	0.0±0.1; 0 (0, 0)
ISBT Grade 3	0.0±0.0; 0 (0, 0)
ISBT Grade 4	0.0±0.2; 0 (0, 0)
Event name, n	
TAD	10 (1.8)
Minor Allergic Reaction	9 (1.6)
TACO	5 (0.9)
Febrile Non-Hemolytic Reaction	4 (0.7)
Hypotensive Reaction	1 (0.2)
Other	1 (0.2)
Possible TRALI	1 (0.2)

[a]Mean/standard deviation); median (interquartile range)

Supplementary Table 9: Antibody test results overall and by participating blood supplier.

	CCP units n=1192	Concentration Median (Q1,Q3) (min, max)
**ADCC ratio**[a]		
All sites	1186 (99.5)	1.359 (0.603, 3.051) (0.127,15.481)
Supplier 1	553 (99.1)	2.284 (0.788, 4.375) (0.127,15.481)
Supplier 2	454 (100.0)	1.122 (0.571, 2.437) (0.127,10.321)
Supplier 3	174 (100.0)	0.583 (0.351, 1.091) (0.127,3.568)
Supplier 4	5 (83.3)	0.844 (0.599, 1.506) (0.196,1.795)
**Anti-RBD ELISA**[b]		
All sites	1186 (99.5)	1.410 (0.959, 1.836) (0.215, 2.943)
Supplier 1	553 (99.1)	1.638 (1.342, 2.029) (0.215, 2.829)
Supplier 2	454 (100.0)	1.102 (0.668, 1.678) (0.304, 2.943)
Supplier 3	174 (100.0)	1.131 (0.810, 1.580) (0.232, 2.423)
Supplier 4	5 (83.3)	1.321 (1.171, 1.718) (0.758, 2.011)
**Anti-S IgG**[c]		
All sites	1186 (99.5)	2853 (1372, 4967) (117, 30402)
Supplier 1	553 (99.1)	3530 (2116, 5547) (117, 15506)
Supplier 2	454 (100.0)	2308 (1248, 4967) (261, 30402)
Supplier 3	174 (100.0)	1333 (578, 2469) (193, 9271)
Supplier 4	5 (83.3)	2477 (1974,2777) (700,10428)
**PRNT-50**[d,e]		
All sites	1191(99.9)	160 (80,320) (10,1280)
Supplier 1	558 (100.0)	160 (160, 640) (40, 1280)
Supplier 2	454(100.0)	80 (20,160) (10, 640)
Supplier 3	174(100.0)	80 (30,160) (10, 640)
Supplier 4	5 (83.3)	160 (160, 160) (80, 160)

[a]Mean of ADCC ratio (1-7 measures). ADCC % with limit 11.3% (equivalent to ratio 0.127); ADCC ratio=ADCC %/(100 -ADCC%)

[b]Mean of anti-RBD ELISA conc. (1-8 measures)

[c]Mean of anti-S IgG conc. (1-7 measures)

[d]Median of PRNT-50 (1-6 measures): PRNT-50 titer=1: n then PRNT=n, PRNT-50 titer≥1:1280 then PRNT=1280, PRNT-50 titer≥1:640 then PRNT=640, PRNT-50 titer=negative then PRNT=10

[e]Includes interpolations (NYBC n=6 and CBS n=1)

Supplementary Table 10: Summary of logistic regression models with standardized continuous log marker dose.

The odds ratio reported in the third column reflects the effect modification corresponding to a 1 standard deviation unit increase in the log transformed and entered marker; in multivariate results markers other than the one labeled in the left column are set to mean log marker dose so the other covariates are zero. The convalescent plasma units transfused in the trial came from 530 distinct plasma donations. Five were missing all serologic marker values except for the original qualifying test and were excluded from analyses. Seven were missing only the neutralization titer value, which was interpolated from the other serologic markers, as described in the statistical analysis plan. All p-values are two-sided with no adjustment for multiple comparisons.

**Intention to treat analysis**	Treatment effect at mean level of antibody, CCP vs. SOC OR (95% CI), p-value	OR for effect modification (95% CI), p-value
**Univariate analysis**		
ADCC assay	1·158 (0·853, 1·572), p=0·3454	0·757 (0·620, 0·924), p=0·0062
Anti-RBD ELISA	1·167 (0·860, 1·584), p=0·3205	0·843 (0·686, 1·035), p=0·1026
Anti-S IgG	1·164 (0·859, 1·579), p=0·3275	1·005 (0·821, 1·231), p=0·9586
Neutralization	1·182 (0·871, 1·604), p=0·2818	0·768 (0·629, 0·937), p=0·0095
**Multivariate analysis**		
	1·143 (0·840, 1·555), p=0·3940	
ADCC assay		0·656 (0·494, 0·871), p=0·0035
Anti-RBD ELISA		1·018 (0·753, 1·376), p=0·9076
Anti-S IgG		1·528 (1·140, 2·049), p=0·0046
Neutralization		0·735 (0·567, 0·953), p=0·0200

A Randomized Open-Label Trial of **CON**valescent Plasma for Hospitalized Adults With Acute **CO**VID-19 **R**espiratory Illness (CONCOR-1)

**Co-Lead Principal-Investigators:**	Dr. Philippe Bégin and Dr. Donald Arnold
**U.S. Principal Investigator:**	Dr. Marshall Glesby
**Brazil Principal Investigator**:	Dr. Luiz Amorim Filho
**Israel Principal Investigator:**	Dr. Martin Ellis
**Sponsor:**	Hamilton Health Sciences
**Country Specific Regulatory Sponsors/Sponsor Representatives:**	Weill Cornell Medical College (United States Only)<br>Fundação Pró-Instituto de Hematologia/Fundarj (Brazil only)<br>Meir Medical Center (Israel only)
**Investigational product:**	COVID-19 Convalescent Plasma
**Protocol #:**	GRAAL-2020-01
**Version and Date of Protocol:**	Version 8.0, 15 January 2021

The study will be conducted according to the protocol and in compliance with Good Clinical Practice (GCP), with the Declaration of Helsinki and with other applicable regulatory requirements.

This document contains confidential information.
Do not copy or distribute without written permission from the Sponsor.

SIGNATURE PAGE

Declaration of Sponsor or Responsible Medical Officers

Title: A Randomized Open-Label Trial of CONvalescent Plasma for Hospitalized Adults With Acute COVID-19 Respiratory Illness (CONCOR-1)

This study protocol was subjected to critical review. The information it contains is consistent with current knowledge of the risks and benefits of the investigational product, as well as with the moral, ethical, and scientific principles governing clinical research as set out in the Declaration of Helsinki [2013] (APPENDIX 1), and the guidelines on Good Clinical Practice.

________________________________	________________________________
Philippe Bégin, MD, PhD, FRCPC	Donald Arnold MD, FRCPC
________________________________	________________________________
Date	Date

DECLARATION OF THE SITE INVESTIGATOR

Title: A Randomized Open-Label Trial of CONvalescent Plasma for Hospitalized Adults With Acute COVID-19 Respiratory Illness (CONCOR-1)

I have read, understood and agree to abide by all the conditions and instructions contained in this protocol.

All documentation for this study that is supplied to me and that has not been previously published will be kept in the strictest confidence. This documentation includes this study protocol, product monograph, electronic case report form (CRF), and other scientific data.

The study will not be commenced without the prior written approval of a properly constituted Research Ethics Board (REB), Institutional Review Board (IRB) or Independent Ethics Committee (IEC). No changes will be made to the study protocol without the prior written approval of the Sponsor and the REB, IRB or IEC, except where necessary to eliminate an immediate hazard to the subjects.

Responsible Investigator of the local study centre

_______________________________________ ______________________

Signature Date

____

Name (block letters)

____

Title (block letters)

____

Institution (block letters)

____

Phone number

PROTOCOL SYNOPSIS

Title: A Randomized, Open-Label Trial of CONvalescent Plasma for Hospitalized Adults With Acute COVID-19 Respiratory Illness (CONCOR-1)

**Development Phase:**	Phase 3
**Sponsor:**	Hamilton Health Sciences
**Study Centers:**	Centre Hospitalier Universitaire Sainte-Justine (CHUSJ), Montreal (Québec Coordinating Centre); McMaster Centre for Transfusion Research, McMaster University, Hamilton (Methods Centre); University of Toronto Quality in Utilization, Education and Safety in Transfusion (QUEST) Research Program (Logistics Centre); Weill Cornell Medicine/New York-Presbyterian (New York Coordinating Centre); Hemorio (Brazilian Coordinating Centre); Meir Medical Center, Kfar Saba, Israel (Israeli Centre)
**Participating Sites**	Approximately 40-60 centres across Canada,one Institution in the United States, approximately 7 centres in Brazil and one in Israel have agreed to participate
**Objective:**	To determine the effect of COVID-19 Convalescent Plasma at reducing the risk of intubation or death at Day 30 in adult patients hospitalized for COVID-19 respiratory illness.
**Investigational product:**	Convalescent plasma from patients who have recovered from COVID-19.
**Control**:	Standard of care
**Population:**	Patients ≥16 years of age (≥18 years of age in the United States, Brazil, and Israel), who admitted for COVID-19 respiratory illness and receiving supplemental oxygen for whom there is ABO-compatible COVID-19 Convalescent Plasma available. Patients are excluded if: 1) COVID-19 respiratory signs or symptoms began more than 12 days before randomization; 2) Currently intubated or a plan for intubation is in place; 3) Plasma is contraindicated (e.g. history of anaphylaxis from transfusion); 4) Decision in place for no active treatment.
**Design:**	Multicentre randomized open-label trial comparing COVID-19 Convalescent Plasma vs. standard of care.

Sample size: 1200 patients

Randomization: 2:1 Randomization (COVID-19 Convalescent Plasma to standard of care) stratified by centre and age (<60 years and ≥60 years). Web accessed randomization with variable block sizes to ensure concealment.

Intervention: **COVID-19 Convalescent Plasma**: one unit of approximately 500 mL apheresis plasma collected from a single donor, or 2 units of approximately 250 mL collected from one or two donors who have recovered from C19 infection.

Control Arm: standard of care

Blinding: Unblinded

Primary outcome: Intubation or death at Day 30

Secondary outcomes:

1. Time to intubation or death
2. Ventilator-free days
3. In-hospital death by Day 90 (at Day 90)
4. Time to in-hospital death
5. Death by Day 30
6. Length of stay in intensive care unit (ICU)
7. Length of stay in hospital
8. Need for extracorporeal membrane oxygenation (ECMO)
9. Need for renal replacement therapy
10. Myocarditis
11. Patient-reported outcome at Day 30
12. Incremental cost per quality-adjusted life year (QALY)
13. CCP transfusion-associated adverse events (AE)
14. Grade 3 and 4 serious adverse events (SAE)
15. Cumulative incidence of Grade 3 and 4 SAEs

Primary Analysis: Based on a 2-sided Wald test of the null hypothesis that intubation or death at Day 30 is the same among individuals receiving COVID-19 Convalescent Plasma or standard of care (relative risk and 95% confidence intervals will be presented).

Interim Analysis: Performed when information on intubation or death at Day 30 is available for 50% of the target sample using an O'Brien-Fleming stopping rule. Blinded sample size re-estimation will be done at that time.

Summary of Distribution of Protocol Versions:

	**Version 3.0**	**Version 4.0**	**Version 5.0**	**Version 6.0**	**Version 7.0**	**Version 7.1**	**Version 8.0**
**Canada**	X		X	X			X
**USA**		X	X	X			X
**Brazil**					X	X	X
**Israel**					X		X

KEY ROLES

**Sponsor**	**Hamilton Health Sciences**<br>1200 Main St W<br>Hamilton, ON L8N 3Z5
**Country Specific Regulatory Sponsors/Sponsor Representatives:**	**Weill Cornell Medical College**<br>1300 York Avenue<br>New York, NY 10065<br>(United States Only)<br><br>**Fundação Pró-Instituto de Hematologia/Fundarj**<br>8 Frei Caneca Street<br>Rio de Janeiro, RJ 20211-030<br>(Brazil only)<br><br>**Meir Medical Centre**<br>59 Tchernichovsky St<br>Kfar Saba 44281, ISRAEL<br>(Isreal only)
**Central Methods Centre**	**McMaster Center for Transfusion Research**<br>**McMaster University**<br>1280 Main Street West, HSC-3H50<br>Hamilton, ON L8S 4K1
**Logistics Center**	**University of Toronto Quality in Utilization, Education and Safety in Transfusion (QUEST) Research Program**<br>**Sunnybrook Health Sciences Centre**<br>2075 Bayview Ave, B204<br>Toronto, ON M4N 3M5
**Québec Co-ordinating Centre**	**Centre Hospitalier Universitaire Sainte-Justine**<br>3175 Côte-Ste-Catherine,<br>Montreal, QC H3T 1C5
**US Co-ordinating Centre**	**Weill Cornell Medical College**<br>1300 York Avenue<br>New York, NY 10065

**Brazil Co-ordinating Centre**	**Hemorio**<br>8 Frei Caneca Street<br>Rio de Janeiro, RJ 20211-030
**Israel Centre:**	**Meir Medical Centre**<br>59 Tchernichovsky St<br>Kfar Saba 44281, ISRAEL
**Investigational Product Supply**	**Héma-Québec (Québec)**<br>4045, Boulevard de la Côte-Vertu,<br>Saint-Laurent, QC H4R 2W7
**Investigational Product Supply**	**Canadian Blood Services (Rest of Canada)**<br>1800 Alta Vista Drive<br>Ottawa, ON K1G 4J5
**Investigational Product Supply**	**New York Blood Center (New York, USA)**<br>310 E 67th St<br>New York, NY 10065
**Investigational Product Supply**	**Hemorio (Brazil)**<br>8 Frei Caneca Street<br>Rio de Janeiro, RJ 20211-030
**Investigational Product Supply**	**Magen David Adom (Israel)**<br>National Blood Services<br>Tel Hashomer, ISRAEL
**Co-principal investigators**	**Donald Arnold, MD, MSc**<br>Hematologist, McMaster University<br><br>**Philippe Bégin, MD, PhD**<br>Clinical immunologist, CHUSJ and CHUM<br><br>**Jeannie Callum, MD**<br>Hematologist, University of Toronto
**Steering Committee**	**See Appendix 2**
**Registration:**	**Clinicaltrials.gov –** NCT04348656

TABLE OF CONTENTS

DECLARATION OF THE SITE INVESTIGATOR 3
PROTOCOL SYNOPSIS 4
TABLE OF CONTENTS 9
LIST OF ABBREVIATIONS AND DEFINITIONS OF TERMS 14
1. INTRODUCTION 16
1.1. Immunologic response to SARS-CoV-2 16
1.2. Passive immunization 17
1.3. Experience with the use of convalescent plasma against other coronavirus diseases 17
1.4. Experience with the use of convalescent plasma in COVID-19 18
1.5. Known potential risks 18
1.6. Known potential benefits 19
1.7. Optimal timing 19
1.8. Optimal dosage 20
2. STUDY OBJECTIVES 21
2.1. Primary objective: 21
2.2. Secondary objectives 21
3. OVERALL DESIGN AND PLAN OF THE STUDY 22
3.1. Overview 22
4. STUDY POPULATION 24
4.1. Eligibility Criteria 24
4.1.1. Inclusion Criteria 24
4.1.2. Exclusion Criteria 24
4.2. Participant recruitment 24
4.3. Randomization Procedures 25
4.4. Intervention: 25
4.4.1. CCP 25
4.4.2. Standard of Care 25
4.5. Participant Withdrawal and Discontinuation of IP 26
4.5.1. Data Collection and Follow-up for Withdrawn Participants 26
4.6. Blinding and Unblinding Procedures 26
4.7. Number of Study Centers 26

4.8. Subject Identification and Randomization 27
4.8.1. Subject Identification 27
4.9. Interim Analysis 27
5. INVESTIGATIONAL TREATMENT 28
5.1. Identity 28
5.2. Mechanism of action 28
5.3. Approved indication 28
5.3.1. Approved Indications for Canada and Israel 28
5.3.2. Approved Indications for the United States and Brazil 28
5.4. Acquisition, Formulation and Packaging 29
5.4.1. Donor recruitment 29
5.4.2. Acquisition and Formulation 31
5.5. Packaging, Labeling and Storage 34
5.5.1. For Canadian Sites: 34
5.5.2. For United States Sites: 34
5.5.3. For Brazilian sites: 35
5.5.4. For the Israeli site: 35
5.6. Anti-SARS-CoV-2 seropositivity 36
5.6.1. ELISA or Chemoluminescence 36
5.6.2. Functional assays 36
5.7. Administration 37
5.8. Dose of CCP 37
5.9. Return and/or Destruction of Investigational Product 37
5.10. Blinding and Breaking the Blind 38
5.11. Investigational Product Accountability 38
5.12. Compliance 38
5.13. Prior and Concomitant Medication 38
6. STUDY OUTCOMES 39
6.1. Primary Outcome: 39
6.2. Secondary Outcomes: 39
7. DURATION OF FOLLOW-UP 39
8. DATA COLLECTION AND SCHEDULE OF EVENTS 40
8.1. Data to be collected from the blood supplier 40

8.2. Subject Screening 40
8.3. Collection of daily hospital data 40
8.4. Baseline/Enrollment (Study Day 1) 42
8.5. Collection of daily hospital data (Study Day 2 until discharge, death or Day 30 post randomization). 42
8.6. Laboratory Test Results 43
8.7. Discharge 43
8.8. Day 30 43
8.9. Day 90 follow-up 43
8.10. Protocol Deviations 44
9. SAFETY VARIABLES 45
9.1. Adverse Event 45
9.2. Serious Adverse Event 45
9.3. Severity of Adverse Events 46
9.4. Causality of Adverse Event 46
9.4.1. Relation to investigational product 46
9.5. Recording Adverse Events occurring after the end of the study 48
9.6. Adverse Event Reporting 48
9.6.1. Serious Adverse Event Reporting 48
9.6.2. Sponsor Reporting of SUSARs: Notifying Health Canada 49
9.6.3. Reporting of Serious Adverse Events to the FDA 49
9.6.4. Reporting of Serious Adverse Events to Agência Nacional de Vigilância Sanitária (ANVISA-Brazil) 50
9.6.5. Reporting of Serious Adverse Events to the Meir Medical Centre Ethics Committee and Israeli Ministry of Health 50
9.6.6. Sponsor Reporting of SUSARs: Notifying Sites 50
9.7. Type and Duration of Follow-up for Adverse Events 50
9.8. Reporting and Entry Timelines 50
9.9. Reporting of Events Other than Serious Adverse Event 51
9.10. Adverse Events to Convalescent Plasma Transfusion 51
9.10.1. Pregnancy 51
9.10.2. Treatment of Overdose of investigational product 52
10. INDEPENDENT COMMITTEES 52
10.1. Independent Data Safety Monitoring Committee 52

10.2. Community Advisory Committee 53
11. SITE MONITORING, AUDITING AND INSPECTING 54
11.1. Informed Consent 54
11.2. Data Quality Assurance 55
11.2.1. Database Management and Quality Control 55
11.3. Case Report Forms and Source Documentation 55
11.3.1. Data Collection 55
11.4. Access to Source Data 56
11.4.1. Remote Monitoring 56
11.4.2. Inspection and Audit Procedures 56
11.5. Data Processing 57
11.6. Archiving Study Records 57
11.7. Good Clinical Practice 57
11.8. Protocol Approval and Amendment 58
11.9. Duration of the Study 58
11.10. Premature Termination of the Study 58
11.11. Confidentiality 59
11.12. Publication Policy 59
11.13. Critical Documents 60
11.14. Clinical Study Report 60
12. STATISTICAL CONSIDERATIONS 61
12.1. Sample Size Considerations 61
12.2. Planned Interim Analyses 61
12.2.1. Safety Review 61
12.2.2. Efficacy Review 61
12.3. Stopping Rules 62
12.4. Final Analysis Plan 62
12.4.1. Primary Efficacy Endpoint 63
12.4.2. Secondary Endpoints 63
12.4.3. Safety Analysis 64
12.4.4. Sub-Group Analysis 65
12.4.5. Exploratory Analysis 65
13. LIST OF APPENDICES 66

14. REFERENCES 67
APPENDIX 1 : Declaration of Helsinki 71
APPENDIX 2: Steering Committee 76

LIST OF ABBREVIATIONS AND DEFINITIONS OF TERMS

ADE	Antibody-dependent enhancement of infection
AE	Adverse Event
AFP	Apheresis Frozen Plasma
CBS	Canadian Blood Services
CCP	COVID-19 Convalescent Plasma
CHU	Centre Hospitalier Universitaire
CI	Confidence Interval
CoV	Coronavirus
CRF	Case report form
ECMO	Extracorporeal Membrane Oxygenation
e-CRF	Electronic Case Report Form
EDC	Electronic Data Capture
ELISA	Enzyme-linked immunosorbent assay
FDA	U.S. Food and Drug Administration
GCP	Good Clinical Practice
HBV	Hepatitis B virus
HCV	Hepatitis C virus
HLA	Human leukocyte antigen
HIV	Human immunodeficiency virus
HUS	Hemolytic Uremic Syndrome
IRB	Institutional Review Board
ICF	Informed Consent Form
ICU	Intensive Care Unit
ICH	International Conference on Harmonization
IDSMC	Independent Data Safety Monitoring Committee
IEC	Independent Ethics Committee
IMP	Investigational Medicinal Product
IND	Investigational New Drug
LAR	Legally Authorized Representive
MedDRA	Medical Dictionary for Regulatory Activities
MERS-CoV	Middle Eastern respiratory syndrome coronavirus
NYBC	New York Blood Center
NYP	NewYork-Presbyterian
OR	Odds ratio
PCR	Polymerase chain reaction
RBD	Receptor-binding domain
REB	Research Ethics Board
RNA	Ribonucleic acid
RT-PCR	Reverse transcriptase polymerase chain reaction
SAE	Serious Adverse Event
SAP	Statistical Analysis Plan
SARS-CoV-1	Severe Acute Respiratory Syndrome Coronavirus 1

SARS-CoV-2	Severe Acute Respiratory Syndrome Coronavirus 2
SpO_2	Peripheral capillary oxygen saturation
SUSAR	Suspected Unexpected Serious Adverse Reaction
TACO	Transfusion associated cardiac overload
TRALI	Transfusion related acute lung injury
TTP	Thrombotic Thrombocytopenic Purpura
USA	United States of America
WCMC	Weill Cornell Medical College
WMA	World Medical Association

1. INTRODUCTION

Coronavirus (CoVs) are positive-sense single stranded enveloped ribonucleic acid (RNA) viruses, many of which are commonly found in humans and cause mild symptoms. Over the past two decades, emerging pathogenic CoVs capable of causing life-threatening disease in humans and animals have been identified, namely severe acute respiratory syndrome coronavirus (SARS-CoV-1) and Middle Eastern respiratory syndrome coronavirus (MERS-CoV).

In December 2019, the Wuhan Municipal Health Committee (Wuhan, China) identified an outbreak of viral pneumonia cases of unknown cause. Coronavirus RNA was quickly identified in some of these patients. This novel coronavirus has been abbreviated as SARS-CoV-2 and has 89% nucleotide identity with bat SARS-like-CoVZXC21 and 82% with that of human SARS-CoV-1[1]. Initially, most of the infections outside China were travel-associated cases in those who had recently visited Wuhan City and were thought to have acquired the virus through contact with infected animals or contact with infected people. There has subsequently been global spread of the virus, including local (human-to-human) spread in many countries, including Canada.

This novel coronavirus has been designated SARS-CoV-2, and the disease caused by this virus has been designated COVID-19. Outbreak forecasting and mathematical modelling suggest that the number of people with COVID-19 will continue to rise in many countries over the coming weeks to months[2]. Global efforts to evaluate novel antivirals and therapeutic strategies to treat COVID-19 have intensified. There is an urgent public health need for rapid development of novel interventions.

At present, there is no specific antiviral therapy for coronavirus infections. Few treatment studies have been conducted because most human coronavirus strains cause self-limited disease and management is supportive care.

1.1. Immunologic response to SARS-CoV-2

Much is still unknown concerning the immune response to SARS-Cov-2 viral infection both in normal individuals and in those that develop severe disease. A previous study of patients with severe SARS-CoV-1 infection found that recovered patients had higher and sustainable levels of both N protein-specific and S glycoprotein-specific neutralizing antibody responses compared to those who succumbed to the disease[3]. This suggests that antibody responses likely plays an important role in determining the ultimate disease outcome of SARS-CoV-infected patients. Immunologic senescence is also a hypothesis to explain the increased risk of complication of death or complication in the elderly population[4].

The details of how this inappropriate humoral response translates to the severe inflammatory phenotype observed in complicating cases are still unknown[5]. One hypothesis is that inefficient clearance of virus leads to disproportionate activation of cellular and innate immunity mechanisms, similar to macrophage activation syndromes seen with viral infections in patients suffering from hemophagocytic lymphohistiocytosis. In fact, ineffective non neutralizing antibodies

against SARS-CoV-1 have been shown to worsen the inflammatory state by contributing to macrophage response activation and skewing[6].

Most therapies currently under investigation for COVID-19 are aimed either at neutralizing the virus (e.g. remdesivir) or at preventing this disproportionate inflammatory response, with ARDS and cytokine storm (e.g. anti-IL-6). Given the suspected link between dysfunctional humoral response and COVID-19 complications, passive immunization approaches with hyperimmune products or convalescent plasma could potentially do both and constitute a promising therapeutic avenue for severe COVID-19.

1.2. **Passive immunization**

Passive immunization consists in the transfer of antibodies from immunized donor to non-immunized individual in order to transfer transient protection against an infective agent. A physiological example of passive immunization is the transfer of maternal IgG antibodies to the foetus through the placenta to confer humoral protection to newborns in the first years of life. Passive immunization differs from active immunization in which the patient develops their own immune response following contact with the infective agent or vaccine.

Passive antibody therapy was used as early as the late 19th century to treat certain infectious diseases prior to the development of antimicrobial therapy in the 1940s[7,8]. Convalescent plasma was also used in the 2013 African Ebola epidemic. A small non-randomized study in Sierra Leone showed an improvement in survival with convalescent whole blood[9] but another study with convalescent plasma showed no improvement in survival[10].

Experience from prior outbreaks with other coronaviruses, such as SARS-CoV-1 finds that convalescent plasma contains neutralizing antibodies to the relevant virus[11]. Viral neutralization is a mechanism whereby antibodies directed against viral surface antigens prevent their interaction with receptors on host cells and fusion of membranes of action of passive immunisation[12]. However, other mechanisms may be possible, such as antibody dependent-cellular cytotoxicity, phagocytosis or virolysis. The relative importance of these mechanisms in passive immunization or in normal defenses against SARS-CoV-2 is unknown. Importantly, studies in HIV have shown that results of assays testing these various functions (e.g. neutralization vs antibody-dependent cytoxicity) may not always correlate and may be complementary [13,14].

1.3. **Experience with the use of convalescent plasma against other coronavirus diseases**

In the 21st century, there were two other epidemics with coronaviruses that were associated with high mortality, SARS-CoV-1 in 2003 and MERS -CoV in 2012. In both outbreaks, the high mortality and absence of effective therapies led to the use of convalescent plasma. The largest study involved a cohort of 80 patients with severe SARS-CoV-1 pneumonia deteriorating despite methylprednisolone in Hong Kong[15]**.** The convalescent plasma dose of 4-5mL/kg was well tolerated in all patients and 33 (41%) were discharged from hospital by day 22 after the onset of symptoms. Three seriously ill patients with SARS in Taiwan were treated with 500 ml of convalescent plasma, which was associated with a reduction in plasma virus titer and good clinical

outcomes[16]. Three patients with MERS-CoV in South Korea were treated with convalescent plasma, but only two of the donors had neutralizing antibody in their plasma[17]. The latter study highlights a challenge in using convalescent plasma; namely, that some people who recover from viral disease may not have high titers of neutralizing antibody[18]. Consistent with this point, an analysis of 99 samples of convalescent sera from patients with SARS-CoV-1 reported that 87 had neutralizing antibody with a geometric mean titer of 1:61 [3].

1.4. **Experience with the use of convalescent plasma in COVID-19**

A non-peer-reviewed phase I study from China enrolled 10 severe patients with COVID-19 who received one dose of 200 mL of convalescent plasma showed a significant improvement in clinical symptoms and oxyhemoglobin saturation within 3 days. Also, within 7 days, all the patients showed different degrees of resorption of lung lesions. In seven patients, viral load became undetectable after transfusion. Importantly, no severe adverse reactions were observed[19].

Another group from China also published their uncontrolled experience with convalescent plasma in 5 ventilated critically ill patients with COVID-19[20]. Following plasma transfusion, body temperature normalized within 3 days in 4 of 5 patients, the SOFA score decreased, and PaO_2/FiO_2 increased within 12 days (range, 172-276 before and 284-366 after). Viral loads also decreased and became negative within 12 days of the transfusion, and SARS-CoV-2–specific enzyme-linked immunosorbent assay (ELISA) and neutralizing antibody titers increased following the transfusion (range, 40-60 before and 80-320 on day 7). Acute respiratory distress syndrome (ARDS) resolved in 4 patients at 12 days after transfusion, and 3 patients were weaned from mechanical ventilation within 2 weeks of treatment. Of the 5 patients, 3 have been discharged from the hospital (length of stay: 53, 51, and 55 days), and 2 are in stable condition at 37 days after transfusion. A third report from China described 4 critically ill patients including a pregnant woman who received convalescent plasma and recovered from their illness[21].

On March 25th, 2020, the United States Food and Drug Administration (FDA) approved the use of convalescent plasma under emergency investigational new drug category[22]. This has allowed licensed physicians to prescribe and obtain approval for this investigational product for the treatment of critically ill patients on very short notice. Our approach is to make this product available to Canadians in a Canada-wide clinical trial. In parallel, the U.S.-based sites and Brazil-based sites will make product available for this trial under an investigational new drug (IND) application from their regulatory agencies. The product is available at the Israeli site with per-patient Ministry of Health approval.

1.5. **Known potential risks**

1. There is a theoretical risk of antibody-dependent enhancement of infection (ADE) whereby virus targeted by non-neutralizing antibodies contained in the convalescent plasma could facilitate entry into and activation of macrophages. This is based on experiments using antibodies from peptide vaccination in macaques. The available evidence from the use of convalescent plasma in patients with SARS-CoV-1 and MERS-CoV[23] and anecdotal evidence of

its use in 245 patients with COVID-19[24], suggest this does not happen when using convalescent plasma, which contains high titres of polyclonal and likely neutralizing antibodies.

2. Another theoretical risk is that antibody administration to those exposed to SARS-CoV-2 may mitigate severe disease but will modify the immune response such that those individuals mount attenuated immune responses, which would leave them vulnerable to subsequent re-infection. In this regard, passive antibody administration before vaccination with respiratory syncytial virus was reported to attenuate humoral but not cellular immunity[25]. Experience with measles prophylaxis has been that active immunization was indeed suppressed when treatment was given in the first 5 days of viral incubation and completely aborted the infection. However, patients for whom treatment was given 5 to 7 days after the incubation period had attenuated disease and later developed normal long-lived immunization. This suggests that active immunization would not be attenuated in patients with active SARS-CoV-2 infection.
3. Finally, there are risks associated with any transfusion of plasma including transmission of blood transmitted viruses (risk <1 in 1 million for all known pathogens, e.g. HIV, HBV, HCV, etc.), allergic transfusion reactions, including anaphylaxis, febrile non-hemolytic transfusion reaction, transfusion related acute lung injury (TRALI), transfusion associated cardiac overload (TACO), and hemolysis should ABO incompatible plasma be administered (not permitted in the CONCOR-1 trial, except for centers where blood group A plasma can be transfused to blood group B or AB patients if AB plasma is not available provided the donor´s anti-B titers are lower than or equal to 1:50). Donors must fulfill all donor requirements for apheresis plasma donation.

1.6. **Known potential benefits**

Potential benefits of COVID-19 convalescent plasma (CCP) include improved survival, improvement in symptoms, decreased risk of intubation for mechanical ventilation, decreased risk of intensive care unit (ICU) admission, shortened hospitalization time and suppression of viral load.

1.7. **Optimal timing**

A general principle of passive antibody therapy is that it is more effective when used early. When used in prophylaxis for measles, treatment within 5 days of contact would usually lead to infection abortion, whereas treatment between 5 and 7 days would lead to infection attenuation[26]. When used for therapy, passive antibodies are most effective when administered shortly after the onset of symptoms. In the Hong Kong SARS cohort, convalescent plasma was found to be most effective in patients who were reverse transcriptase polymerase chain reaction (RT-PCR) positive and seronegative for coronavirus[15]. Passive antibody therapy for pneumococcal pneumonia was most effective when administered shortly after the onset of symptoms and there was no benefit if antibody administration was delayed passed the third day of disease[27]. With passive antibody therapy for SARS-CoV-1, Chen and al. found that administration of convalescent plasma before Day 14 of illness resulted in a higher discharge rate by day 22 compared to participants who

received it later than Day 14. Two controlled cohort studies from the Spanish influenza pandemic reported efficacy only if treatment was administered within 5 days of onset of viral pneumonia (around day 12 of symptom onset). In the 2 studies, the risk of death was reduced [odd ratios (OR) for mortality = 0.60 and OR=0.31, respectively] when administered before 5 days, compared to an OR=1.13 and OR=0.93 when given after 5 days[28,29]. In a cohort of H1N1 influenza patients treated with convalescent plasma from 2013, the risk of death was reduced (OR = 0.14) when treatment was administered in the first 4 days, but the risk of death was increased when administered after 5 days [30]. Given the natural evolution of COVID-19 which involves a slower progression of initial symptoms compared to influenza (Figure 1), CONCOR-1 will enrol patients who are within 12 days of respiratory symptom onset and not yet intubated.

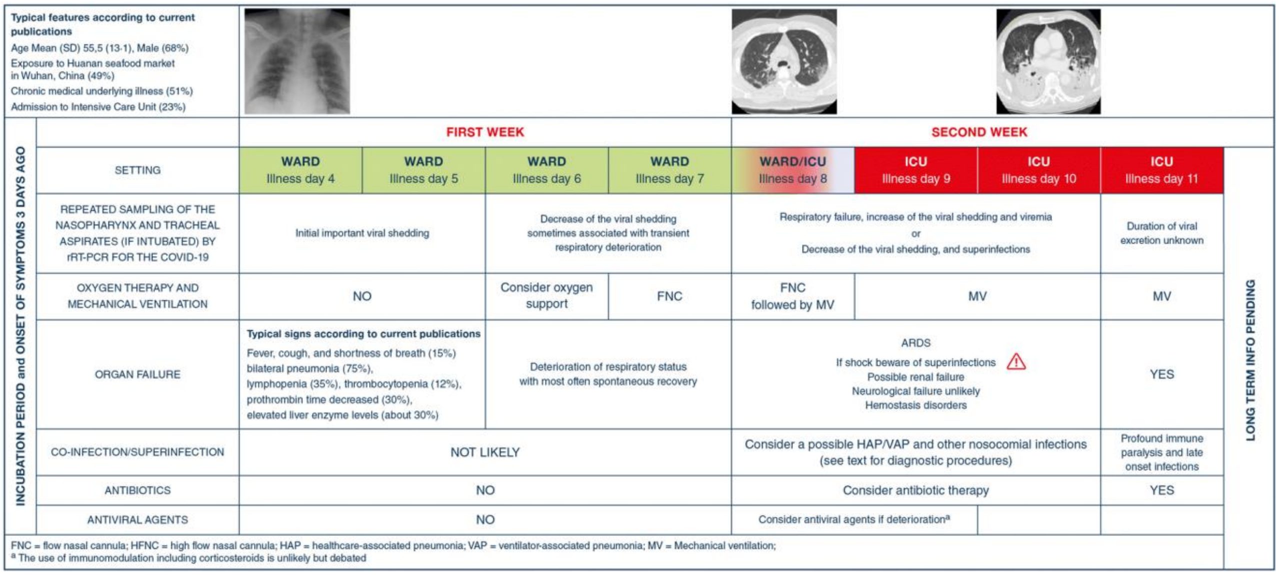

Figure 1 (from Bouadma et al Int Care Med 2020) describes what is known about the potential patient course of COVID-19 pneumonia. Plasma therapy is expected to be most effective if given before the red zone[31].

1.8. **Optimal dosage**

The optimal dosage for passive antibody therapy is unknown. The following table presents dosages used in previous studies.

Indication	Dosages used
Spanish influenza pneumonia[32]	Single dose of 75 to 500mL<br>*Pediatric:* single dose of 50mL
SARS-CoV1 pneumonia[15,16,23,33–35]	Single dose of 50 to 500ml
Avian influenza (H5N1) pneumonia[36–38]	3 doses of 200mL<br>5 doses of 100mL
Influenza A (H1N1) pneumonia[39,40]	Single dose of 500mL
Ebola infection[41]	2 to 6 doses of 500mL
COVID-19[19,20]	Single dose of 200ml

	Single dose of 400 ml
Measles prophylaxis[26,42]	Single dose of 15mL<br>*Pediatrics:* 2.5 to 15mL
Mumps prophylaxis[43]	Single dose of 40mL

2. STUDY OBJECTIVES

2.1. **Primary objective:**

To determine the effect of CCP for adults admitted to hospital with COVID-19 respiratory illness requiring supplemental oxygen on rates of death and intubation at 30 days.

2.2. **Secondary objectives**

Safety: Determine the safety of CCP. We will capture adverse events (AE) associated with the transfusion of CCP including suspected TRALI, and all other serious adverse events (SAE).

Relevant data will be captured during the trial to provide information on the following:

- Time to intubation or death
- Ventilator-free days
- In-hospital death by Day 90
- Time to in-hospital death
- Death by Day 30
- Length of stay in intensive care unit (ICU)
- Length of stay in hospital
- Need for extracorporeal membrane oxygenation (ECMO)
- Need for renal replacement therapy
- Myocarditis
- Patient-reported outcome at Day 30
- Incremental cost per quality-adjusted life year (QALY)
- Transfusion-associated adverse events (AE)
- Grade 3 and 4 serious adverse events (SAE)
- Cumulative incidence of Grade 3 and 4 SAEs

Exploratory studies: To determine the association between CCP characteristics and rates of intubation or death in hospital at day 30 in participants receiving CCP. CCP characteristics to be assessed include the amount of antibodies against the SARS-CoV-2 spike protein and its receptor-binding domain (RBD),its neutralizing function, its Fc function and clinical characteristics of donors.

3. OVERALL DESIGN AND PLAN OF THE STUDY

3.1. Overview

Research Question – Does the transfusion of CCP collected from donors who have recovered from COVID-19 infection and who have detectable anti-SARS-CoV-2 antibodies reduce the risk of intubation or death at Day 30 in adults who are admitted to hospital with COVID-19 respiratory illness compared to standard of care?

Study Hypothesis - We hypothesize that treating hospitalized adult patients with COVID-19 respiratory illness will reduce the risk of intubation or death at Day 30. In addition, we hypothesize that other outcomes will be improved including risk of ICU admission, length of stay in ICU and length of stay in hospital.

Study Design: Multicentre open-label randomized controlled trial. An overview of the study design is included in the flow chart below.

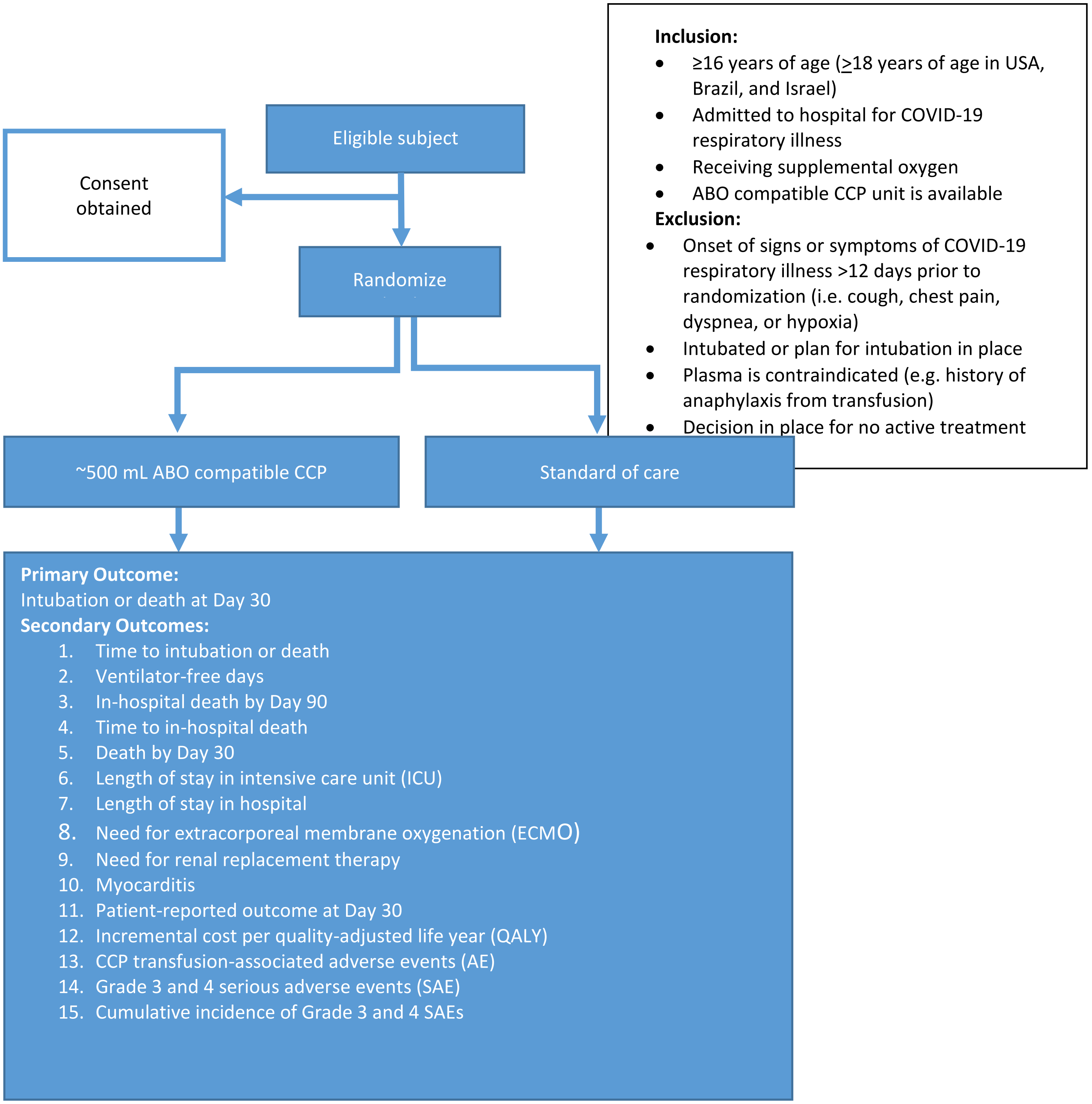
Inclusion:
• ≥16 years of age (≥18 years of age in USA, Brazil, and Israel)
• Admitted to hospital for COVID-19 respiratory illness
• Receiving supplemental oxygen
• ABO compatible CCP unit is available
Exclusion:
• Onset of signs or symptoms of COVID-19 respiratory illness >12 days prior to randomization (i.e. cough, chest pain, dyspnea, or hypoxia)
• Intubated or plan for intubation in place
• Plasma is contraindicated (e.g. history of anaphylaxis from transfusion)
• Decision in place for no active treatment
Eligible subject
Consent obtained
Randomize
~500 mL ABO compatible CCP
Standard of care
Primary Outcome:
Intubation or death at Day 30
Secondary Outcomes:
1. Time to intubation or death
2. Ventilator-free days
3. In-hospital death by Day 90
4. Time to in-hospital death
5. Death by Day 30
6. Length of stay in intensive care unit (ICU)
7. Length of stay in hospital
8. Need for extracorporeal membrane oxygenation (ECMO)
9. Need for renal replacement therapy
10. Myocarditis
11. Patient-reported outcome at Day 30
12. Incremental cost per quality-adjusted life year (QALY)
13. CCP transfusion-associated adverse events (AE)
14. Grade 3 and 4 serious adverse events (SAE)
15. Cumulative incidence of Grade 3 and 4 SAEs

4. STUDY POPULATION

4.1. Eligibility Criteria

4.1.1. Inclusion Criteria

1. ≥16 years of age (≥18 years of age in the United States, Brazil, and Israel)
2. Admitted to hospital for confirmed COVID-19 respiratory illness
3. Receiving supplemental oxygen
4. 500 mL of ABO compatible CCP is available

4.1.2. Exclusion Criteria

1. Onset of signs or symptoms of COVID-19 respiratory illness >12 days prior to randomization (eg. cough, chest pain, dyspnea, or hypoxia)
2. Intubated or plan in place for intubation
3. Plasma is contraindicated (e.g. history of anaphylaxis from transfusion)
4. Decision in place for no active treatment

Potential patients will be approached for informed consent. After consent is obtained, a screening ABO blood group will be collected if not already performed during the current hospital admission. Co-enrolment in other trials is allowed. For patients who fail screening, we will collect only the reason for screen failure with no personal identifiers.

Pregnant patients will not be excluded from the study if they meet eligibility criteria, except for sites where pregnant patients are not permitted to participate in the research as per local regulatory requirements. While young adults and pregnant women have been infrequently reported to present with complications, data from current COVID-19 wards indicate that those who develop severe COVID-19 pneumonia appear to have a similar risk of progression to intubation and critical disease than older patients. At the CHUM in Montreal, over half of COVID-19 patients admitted to the ICU were younger than 55 years of age. From a safety standpoint, CCP was well tolerated in one pregnant patient in a case series reported from China[21]. Therefore, based on current eligibility criteria, the same risk/benefit considerations apply to pregnant and younger patients as compared to the elderly, hence the reason not to exclude them from this trial.

4.2. Participant recruitment

Only hospitalized COVID-19 patients are eligible so recruitment efforts will be focused on identifying consecutive patients admitted to hospital with acute COVID-19 infection. No other external recruitment efforts are planned. At each participating hospital, a process for immediately identifying admitted patients with COVID-19 will be established. The mechanism of alerting the research assistants (RA) will be determined by local site principal investigator.

Patients who are confirmed to have COVID-19 infection and are admitted to hospital will be assessed for eligibility. Consent may be obtained verbally, given the infection control precautions

in place for these participants, as permitted by local research ethics committees. The RA (or a bedside physician if deemed acceptable by local research ethics boards [REB]) will obtain consent from the patient or legally authorized representative (LAR) by phone or videoconference. After consent is obtained, the RA will confirm eligibility remotely through a review of the electronic medical record and discussion with the bedside physician/nurse. Informed consent will be obtained remotely and may use a variety of visual aids (video brochure, a visual decision board). Documented written consent will be obtained from the patient or LAR as soon as possible as required by local REBs. At the time of assessing eligibility the RA will confirm with the site storing CCP that an ABO-compatible CCP plasma unit is available (for certain sites, group A plasma with low-titer anti-B (≤1:50) is allowed to be transfused to group B or AB patients). If 500 mL of CCP is available and eligibility criteria are met, the RA will randomize the patient. The RA will notify the blood bank of the allocation and a marker will be placed in the Laboratory Information System and the patient chart indicating that the patient is enrolled in the CONCOR-1 trial. For patients randomized to CCP, the RA will notify the bedside physician to order the study product. The CCP will be thawed in the blood bank, issued, and infused by the bedside nurse within 24 hours of randomization.

4.3. **Randomization Procedures**

Patients will be randomized in a 2:1 ratio (CCP to standard of care). Patients will be randomized using a secure, concealed, computer-generated, web-accessed randomization sequence. Randomization will be stratified by centre and age (<60 and ≥ 60 years). Within each stratum, variable permuted block sizes will be used. This approach will ensure that concealment of the treatment sequence is maintained.

4.4. **Intervention:**

Patients will be assigned to the experimental treatment (CCP) or the control (standard of care).

4.4.1. **CCP**

Patients will receive approximately 500 mL of CCP (from one single-donor unit of 500 mL or 2 units of 250 mL from one or two donors) collected by apheresis from donors who have recovered from COVID-19 and frozen (1 year expiration date from date of collection). The plasma unit will be thawed as per standard blood bank procedures and infused into the patient slowly over 4 hours or as per local procedures. When administering 2 units of 250 mL, the 2nd unit should be administered immediately after the first unit, and we recommend that it should be given within 12 hours after the first unit. The patient will be monitored for adverse events as per each site's policies. Details regarding the CCP are summarized in Section 5.0.

4.4.2. **Standard of Care**

Patients receiving standard of care will receive all medical care as per routine practices at each site.

4.5. **Participant Withdrawal and Discontinuation of IP**

Subjects in this clinical trial may discontinue the investigational product for any of the following reasons:

- Subject requests to discontinue the investigational product during transfusion or to be removed from clinical trial activities for any reason (reason for withdrawal will be requested)
- Occurrence of any medical condition or circumstance that exposes the patient to substantial risk and/or does not allow the patient to adhere to the requirements of the protocol
- Any SAE, clinically significant AE, severe laboratory abnormality, intercurrent illness, or other medical condition deemed related to the investigational product that indicates to the Investigator that continued participation is not in the best interest of the patient (in such case the investigational product must be discontinued).
- Patient fails to comply with protocol requirements or study-related procedures

Unless the patient withdraws consent, those who discontinue the investigational product during the infusion should remain in the study and be included in the intention-to-treat population. The reason for participant discontinuation of investigational product should be documented in the case report form where known.

Should the participant withdraw consent or requests discontinuation for any reason, whenever possible, the patient or LAR will be asked if the patient can be followed for outcome and safety evaluations (where permitted by the local REB).

4.5.1. **Data Collection and Follow-up for Withdrawn Participants**

Participants who withdraw from this study or who are lost to follow-up after randomization will not be replaced. The reason for patient discontinuation from the study will be recorded on the appropriate case report form. In the case of a participant's lost to follow-up, attempts to contact the patient will be made and documented in the patient's study file.

4.6. **Blinding and Unblinding Procedures**

This is an open-label trial. The decision to perform the trial unblinded was based on the following reasons: (1) to allow for participation of academic and non-academic hospital sites where masking procedures are not possible given the required short implementation timelines; (2) to avoid exposing patients to the risks of a standard plasma arm as the placebo has no anticipated benefit; (3) to avoid the need to create an inert placebo (e.g. coloured saline) including the need to mask, which would cause significant disruption of workflow and additional use of PPE during the pandemic.

4.7. **Number of Study Centers**

The number of hospital sites across Canada will be ~40-60 sites, including sites in every Province and potentially every territory in both academic and community-based hospitals. There will be a

gradual onboarding of sites starting with larger centres in Québec and Ontario where the burden of the pandemic is expected to be the largest. We will use Clinical Trials Ontario (CTO), the central REB for Ontario sites and expedite time to first patient treated. The multi-centre ethics evaluation process will be similarly used in Québec. Thereafter sites will be on-boarded based on size and the current distribution of cases of COVID-19 to ensure the greatest access to the trial based on need. In addition, to enhance the rapidity of study enrollment, one New York City Institution in the U.S. will participate, as well as several sites in Brazil and one site in Israel.

4.8. **Subject Identification and Randomization**

4.8.1. **Subject Identification**

Subjects in this study will have specific screening number. Subjects failing screening will retain their screening number. The screening number for each subject will be a combination of the site number plus a number assigned to the subject according to her/his chronological order of screening at that site. The screening number will be used as the subject identifier throughout the study.

4.9. **Interim Analysis**

A single interim analysis is planned when the primary outcome (intubation or mortality at Day 30) is available for 50% of the target sample. An O'Brien- Fleming stopping rule[44] will be used at that time, but treated as a guideline, so there is minimal impact on the threshold for statistical significance for the final significance test of the primary outcome.

5. INVESTIGATIONAL TREATMENT

5.1. Identity

The investigational product consists of approximately 500 mL Apheresis Frozen Plasma (AFP) collected from patients who have recovered from COVID-19.

5.2. Mechanism of action

Transfusion of AFP from COVID-19 convalescent patients allows the transfer of donor neutralizing antibodies directed against SARS-CoV-2 antigens in the recipient, thus allowing the generation of passive immunization. Naturally produced human antibody are polyclonal, meaning they are directed against a variety of different viral antigens and epitopes allowing for a general neutralizing effect against the virus rather than focussing on a specific target. Administration of CCP has been associated with rapid decrease in viral load. It is also possible that passive immunization contributes to improved cell-mediated immunity by favoring the phagocytosis and presentation of viral antigens to host T cells.

5.3. Approved indication

5.3.1. Approved Indications for Canada and Israel

AFP transfusions are currently indicated in Canada for the management of:

1. Bleeding patients or patients undergoing invasive procedures who require replacement of multiple plasma coagulation factors
2. Patients with massive transfusion with clinically significant coagulation abnormalities
3. Patients on warfarin who are bleeding of need to undergo an invasive procedure before vitamin K could reverse the warfarin effect, and where prothrombin complex concentrate is not available or is contraindicated
4. Patients with selected coagulation factor or with rare specific plasma protein deficiencies for which a more appropriate alternative therapy is not available
5. Preparation of reconstituted whole blood for exchange transfusion in neonate
6. Patients with thrombotic thrombocytopenic purpura (TTP) or hemolytic uremic syndrome (HUS) undergoing plasma exchange

5.3.2. Approved Indications for the United States and Brazil

AFP transfusions are currently indicated in the United States for the management of:

1. Preoperative or bleeding patients who require replacement of multiple plasma coagulation factors (eg. Liver disease, DIC).
2. Patients undergoing massive transfusion who have clinically significant coagulation deficiencies.
3. Patients taking warfarin who are bleeding or need to undergo an invasive procedure before vitamin K could reverse the warfarin effect or who need only transient reversal of warfarin effect.

4. Transfusion or plasma exchange in patients with thrombotic thrombocytopenic purpura
5. Management of patients with selected coagulation factor deficiencies, congenital or acquired, for which no specific coagulation concentrates are available.
6. Management of patients with rare specific plasma protein deficiencies, such as C1 inhibitor, when recombinant products are unavailable.

5.4. Acquisition, Formulation and Packaging

For Canadian sites, the investigational product will be provided by Héma-Québec and Canadian Blood Services. For United States sites, the investigational product will be provided by the New York Blood Center (NYBC). For Brazilian sites, the investigational product will be provided by Hemorio. For the Israeli site the product will be provided by the Magen David Adom National Blood Services.

5.4.1. Donor recruitment

5.4.1.1. Donor recruitment for Canadian Sites

Recovered COVID-19 patients will be identified as potential donors in collaboration with provincial public health services, local health authorities and individual co-investigators involved in the study. Potential donors may also be recruited following self-identification on the routine donor questionnaire or through social media. They will be contacted by phone and invited to participate in the program as potential donors. After obtaining consent and reviewing donor selection criteria, eligible participants will be directed to a Héma-Québec collection or Canadian Blood Services apheresis collection site in their area to donate.

Criteria for donors: All donors will need to meet the criteria set forth in the Manuals of donor selection criteria in use at Héma-Québec or Canadian Blood Services.

In addition, donors will require:

- Prior diagnosis of COVID-19 documented by a PCR test at time of infection or by positive anti-SARS-CoV-2 serology following infection
- Male donors, or females with no pregnancy history or with negative anti-HLA antibodies
- At least 6 days since last plasma donation
- Provided informed consent
- A complete resolution of symptoms at least 14 days prior to first donation.

Criteria for donation are subject to change based on future revision of Health Canada guidance.

5.4.1.2. Donor recruitment for United States Sites

Recovered COVID-19 patients are being recruited through the New York Blood Center and Weill Cornell Medicine in separate protocols. Potential donors can self-refer via websites but also be referred by physicians or identified via the medical record system. Only donors with laboratory-confirmed history of COVID-19 will be screened. After providing consent and reviewing FDA and

NYBC donor eligibility criteria, donors are screened for the presences of SARS-CoV-2 virus in the nasopharynx if screening within 14 days of complete resolution in accordance with current FDA guidance. Criteria for donation are subject to change based on future revision of FDA guidance. Those found to be eligible will be referred to NYBC for donation.

Criteria for donors:

- Provision of informed consent
- Aged 18 to 70 years. Donors are not longer eligible after their 71st birthday.
- Documented molecular diagnosis of SARS-CoV-2 by RT-PCR by nasopharyngeal swab, oropharyngeal swab, or sputum or detection of anti-SARS-CoV-2 IgG in serum.
- Complete resolution of COVID-19 symptoms at least 14 days prior to donation
- Not currently pregnant or pregnant within 6 weeks by self-report
- Male donors, or females with no pregnancy history or with negative anti-HLA antibodies
- Meets blood donor criteria specified by NYBC, which is consistent with FDA regulations.

Donors will be allowed to donate every 7 days. The following information will be collection from donors: ABO group, sex, age, date of onset of symptoms (when available), date of resolution of symptoms (when available) and CCP collection date(s).

Sterile segments of frozen plasma will be attached to all units consisting of at least 2 mL of plasma volume. These segments will be submitted to the Centre Hospitalier de l'Université de Montréal Research Center for testing of SARS-COV-2 antibody titer and neutralizing antibody titer.

5.4.1.3. **Donor recruitment for Brazil**

Recovered COVID-19 patients will be identified as potential donors in collaboration with provincial public health services, local health authorities, individual co-investigators involved in the study. Potential donors may also be recruited following self-identification on the routine donor questionnaire or through social media. They will be contacted by phone and invited to come to Hemorio in order to undergo a medical examination that will include the collection of blood samples. If they are approved as plasma donors, after obtaining consent, they are invited to make an appointment to come back and donate plasma, as soon as possible.

Criteria for donors: All donors will need to meet the criteria set forth in the Manuals of donor selection criteria in use at Hemorio and in Brazilian specific regulations.

In addition, donors will require:

- Aged 18 to 60 years. Donors are no longer eligible after their 61st birthday
- Prior diagnosis of COVID-19 documented by a PCR test at time of infection or by positive anti-SARS-CoV-2 serology following infection
- Male donors, or females with no pregnancy history or with negative anti-HLA antibodies
- At least 7 days since last plasma donation
- Provided informed consent

- 7-day interval between each donation, limited to 4 donations over a 2 month period
- A complete resolution of COVID-19 symptoms at least 14 days prior to first donation.

Criteria for donation are subject to change based on future revision of Brazilian federal regulation.

5.4.1.4. Donor recruitment for Israel

Recovered COVID-19 patients will be identified as potential donors in collaboration with national health services, local health authorities and individual co-investigators involved in the study. Potential donors may also be recruited following self-identification on the routine donor questionnaire or through social media. They will be contacted by phone and invited to participate in the program as potential donors. After obtaining consent and reviewing donor selection criteria, eligible participants will be directed to the Magen David Adom National Blood Services apheresis collection site in their area to donate.

Criteria for donors: All donors will need to meet the criteria set forth in the Manuals of donor selection criteria in use at Magen David Adom National Blood Services

In addition, donors will require:

- Prior diagnosis of COVID-19 documented by a PCR test at time of infection or by positive anti-SARS-CoV-2 serology following infection
- Male donors, or females with no pregnancy history or with negative anti-HLA antibodies
- At least 14 days since last plasma donation
- Provided informed consent
- A complete resolution of symptoms at least 14 days prior to first donation.

Criteria for donation are subject to change based on future revision of Israel Ministry of Health guidance.

5.4.2. Acquisition and Formulation

5.4.2.1. For Canadian Sites

Plasma will be collected by apheresis from qualified donors who have consented to donate, using the equipment and technologies already in operation at Héma-Québec and Canadian Blood Services following standard operating procedures.

Because apheresis collection allows for plasma only collection while blood cells are returned to the donor, donor are eligible to donate every 6 days (at minimum). This will permit the collection of CCP from the same donor at weekly intervals. SARS-CoV-2 antibody titers will be monitored at each donation to ensure the presence of sufficient amounts of antibody for the treatment of patients suffering from COVID-19.

Plasma collection using the TRIMA® Accel™ technology (Terumo BCT)
The TRIMA® Accel™ technology allows the automated collection of different blood components and contains programs for plasma collection only. Disposable sterile kits for plasma collection are available (kits with 3 or 4 plasma bags). Depending on the donor characteristics (calculated blood volume), plasma volumes up to 750 mL can be collected. The procedure starts with the collection of a small volume of blood in a sampling bag; this blood will be used for regulatory analyses at Héma-Québec and Canadian Blood Services (transfusion transmissible diseases) and to establish the anti-SARS-CoV-2 seropositivity. During collection, blood is mixed with ACD-A (anticoagulant citrate dextrose, solution A), plasma is separated from blood cells by centrifugation and collected in 2 or 3 bags (depending on the target volume that has been predefined) based on the estimated total blood volume of the donor whereas blood cells are returned to the donor.

Plasma collection using the PCS®2 technology (Haemonetics)
This automated system is used to collect plasma only. Samples required for regulatory analyses and antibody seropositivity determination are taken directly from the collected unit after plasma collection is completed. During collection, blood is mixed with anticoagulant (sodium citrate 4%) to avoid clotting and centrifuged in a single use, sterile centrifugation bowl to separate plasma from blood cells. When separation is completed, plasma is automatically transferred to the collection bag and thereafter, blood cells are returned to the donor.

5.4.2.2. **For United States Sites**

Plasma will be collected by apheresis from qualified donors who have consented to donate using the equipment and technologies already in operation at the New York Blood Center following standard operating procedures. Donors are eligible to donate every 7 days. This will permit collection of CCP from the same donor at weekly intervals.

Plasma collection using the TRIMA® Accel™ technology (Terumo BCT)

The TRIMA® Accel™ technology allows the automated collection of different blood components and contains programs for plasma collection only. Disposable sterile kits for plasma collection are available (kits with 3 or 4 plasma bags). Dependent on the donor characteristics (calculated blood volume), plasma volumes up to 750 mL can be collected. The procedure starts with the collection of a small volume of blood in a sampling bag; this blood will be used for regulatory analyzes at NYBC (transfusion transmitted disease testing). During collection, blood is mixed with ACD-A (anticoagulant citrate dextrose, solution A), plasma is separated from blood cells by centrifugation and collected in 2 or 3 bags (depending on the target volume that has been pre-defined) based on the estimated total blood volume of the donor whereas blood cells are returned to the donor.

Plasma collection using the Alyx® component collection system (Fresenius Kabi)

This automated system can be used to collect plasma only. Samples required for regulatory analyses are taken directly from the collected unit after plasma collection is completed. During collection, blood is mixed with ACD-A to avoid clotting and centrifuged in a single use, sterile centrifugation bowl to separate plasma from blood cells. When separation is completed, plasma is

automatically transferred to the collection bag and thereafter, blood cells are returned to the donor.

5.4.2.3. **For Brazilian sites**

Plasma collection using the MCS®+ technology (Haemonetics)

This automated system can be used to collect plasma. Samples required for regulatory analyses and antibody seropositivity determination are taken directly from the collected unit after plasma collection is completed. During collection, blood is mixed with anticoagulant (sodium citrate 4%) to avoid clotting and centrifuged in a single use, sterile centrifugation bowl to separate plasma from blood cells. When separation is completed, plasma is automatically transferred to the collection bag and thereafter, blood cells are returned to the donor. Depending on donor characteristics, plasma volumes up to 600 mL can be collected.

5.4.2.4. **For the Israeli site**

Plasma will be collected by apheresis from qualified donors who have consented to donate, using the equipment and technologies already in operation at Magen David Adom Nationa Blood Services following standard operating procedures.

Because apheresis collection allows for plasma only collection while blood cells are returned to the donor, donor are eligible to donate every 14 days (at minimum). This will permit the collection of CCP from the same donor at weekly intervals. SARS-CoV-2 antibody titers will be monitored at each donation to ensure the presence of sufficient amounts of antibody for the treatment of patients suffering from COVID-19.

Plasma collection using the TRIMA® Accel™ technology (Terumo BCT)

The TRIMA® Accel™ technology allows the automated collection of different blood components and contains programs for plasma collection only. Disposable sterile kits for plasma collection are available (kits with 3 or 4 plasma bags). Depending on the donor characteristics (calculated blood volume), plasma volumes up to 750 mL can be collected. The procedure starts with the collection of a small volume of blood in a sampling bag; this blood will be used for regulatory analyses at Magen David Adom National Blood Services (transfusion transmissible diseases) and to establish the anti-SARS-CoV-2 seropositivity. During collection, blood is mixed with ACD-A (anticoagulant citrate dextrose, solution A), plasma is separated from blood cells by centrifugation and collected in 2 or 3 bags (depending on the target volume that has been predefined) based on the estimated total blood volume of the donor whereas blood cells are returned to the donor.

Plasma collection using the PCS®2 technology (Haemonetics)

This automated system is used to collect plasma only. Samples required for regulatory analyses and antibody seropositivity determination are taken directly from the collected unit after plasma collection is completed. During collection, blood is mixed with anticoagulant (sodium citrate 4%)

to avoid clotting and centrifuged in a single use, sterile centrifugation bowl to separate plasma from blood cells. When separation is completed, plasma is automatically transferred to the collection bag and thereafter, blood cells are returned to the donor.

5.5. **Packaging, Labeling and Storage**

Labels bearing the ISBT 128 donation identification will be printed and placed on all collection bags and blood tubes before donation. After donation, plasma will be shipped to a component processing facility at Héma-Québec, Canadian Blood Services, or New York Blood Center and will be frozen at -18°C within 8-24 hours of collection. In Hemorio and in Magen David Adom National Blood Services the plasma will be collected at the same place where the component process facility is located. When results of regulatory analyses become available (this usually takes less than 48 hours) and the product can be released for transfusion, a new label will be applied on the plasma bag showing all product information including the ABO group. The plasma will also be labeled as “convalescent plasma” at Canadian, Brazilian, and Israeli sites and “apheresis plasma” at United States sites.

5.5.1. **For Canadian Sites:**

The sponsor shall ensure that the drug bears a label that sets out the following information in both official languages:
(a) a statement indicating that the drug is an investigational drug to be used only by a qualified investigator;
(b) the name, number or identifying mark of the drug;
(c) the expiration date of the drug;
(d) the recommended storage conditions for the drug;
(e) the lot number of the drug;
(f) the name and address of the sponsor;
(g) the protocol code or identification.

A segregated storage area will be set up and used to keep CCP apart from the other plasma products. A product list will be kept up to date in order to know at all times the volume of CCP available for each ABO blood group. When requested for transfusion, the products will be shipped to the hospital through the regular transportation system for blood products or to pre-identified regional “hub” hospitals where inventory will be maintained. Large sites and those at a geographical distance of more than 200 km may be permitted to hold a stock of one 500 mL dose for each ABO blood group and smaller sites will only be shipped the investigation product once a patient has been randomized to the active treatment arm.

5.5.2. **For United States Sites:**

The sponsor shall ensure that the drug bears a label that sets out the following information in English:

(a) A statement indicating that the drug is a new drug limited by federal (or United States) law for investigational use only;
(b) The name, number or identifying mark of the drug;

(c) The expiration date of the drug;
(d) The recommended storage conditions of the drug;
(e) The donor identification number

After collection, processing, and completion of donor regulatory analyses, the product will be shipped to the hospital blood bank for storage. A segregated storage area will be set up and used to keep the CCP apart from the other plasma products. A product list will be kept up to date in order to know at all times the number of units available and their corresponding blood volumes and ABO types.

5.5.3. **For Brazilian sites:**

The sponsor shall ensure that the drug bears a label that sets out the following information in both official languages:
(a) a statement indicating that the drug is for research use and should only be used by a qualified investigator;
(b) the name, number or identifying mark of the drug;
(c) the expiration date of the drug;
(d) the recommended storage conditions for the drug;
(e) the donor identification number;
(f) the name and address of the blood center;

A segregated storage area will be set up and used to keep CCP apart from the other plasma products. A product list will be kept up to date in order to know at all times the volume of CCP available for each ABO blood group. When requested for transfusion, the products will be shipped to the hospital through the regular transportation system for blood products (to hospitals located in Rio de Janeiro area) and by plane to hospital located in other states, where inventory will be kept. Sites at a geographical distance of more than 200 km may be permitted to hold a stock of three 500 mL doses for each ABO blood group.

5.5.4. **For the Israeli site:**

The sponsor shall ensure that the drug bears a label that sets out the following information in Hebrew:
(a) The name, number or identifying mark of the drug;
(b) The expiration date of the drug;
(c) The recommended storage conditions of the drug;
(d) The donor identification number

After collection, processing, and completion of donor regulatory analyses, the product will be shipped to the hospital blood bank for storage. A segregated storage area will be set up and used to keep the CCP apart from the other plasma products. A product list will be kept up to date in order to know at all times the number of units available and their corresponding blood volumes and ABO types.

5.6. **Anti-SARS-CoV-2 seropositivity**

There is currently no clinically validated method to characterize the therapeutic potency of COVID-19 convalescent plasma. Importantly, non-convalescent plasma selected from the general population based on titers of neutralizing antibodies did not show efficacy in the treatment of influenza in 3 large RCTs questioning reliance of such assay to predict efficacy[45–47]. Because the validity of such assays to determine the therapeutic value of CCP constitutes an important clinical question, the neutralizing activity of plasma samples will be documented for all samples and correlated with treatment effect as an exploratory objective. These assays can be performed before or after the plasma has been released.

However, as a minimal requirement, anti-SARS-CoV-2 seropositivity must be demonstrated on donor sample prior to release of product. This can be done by ELISA or with a neutralizing assay. In the U.S sites, seropositivity may be assessed after transfusion of the product for a minority of donors. The majority of donors will be referred by the treating center to the donor center and pre-screened for seropositivity prior to donation. However, if CCP units from the referred donors are unavailable, a general pool of CCP plasma can be used with seropositivity assessed from a tail segment following transfusion.

5.6.1. **ELISA or Chemoluminescence**

The ELISA or Chemoluminescence will specifically test for antibodies directed against the receptor binding domain (RBD) of SARS-CoV-2 glycoprotein S, which have been reported to correlated with plasma neutralizing activity (R^2=0.96) [48].

The American Association of Blood Banks (AABB) provides no guidance with regards to a specific titer cut-off value to qualify convalescent plasma as the optimal titer is currently unknown. The cut-off for seropositivity will be set as the mean value + 3 SD of the ELISA signal obtained with SARS-CoV-2 negative plasma (pool of plasma samples collected before 2020) at a 1:100 plasma dilution.

5.6.2. **Functional assays**

Functional neutralization assays can also be used as a criteria to confirm seropositivity. The most direct method involves adding the diluted plasma to a specific number of infectious particles (in this case SARS-CoV-2 virus), plating the virus, and measuring the number of plaques that can still be formed. This provides insights into the levels of neutralizing antibodies and for treating Covid-19. If used to qualify donation, a dilution of 1/160 will be used as threshold neutralizing titer will be used to confirm donor eligibility.

The antibody-dependent cell cytotoxicity assay, which measure Fc function of antibodies will also be performed on all plasma samples as per the study's exploratory objective but will not be used as a criteria to qualify donations.

5.7. **Administration**

The investigational product will be administered by a bedside nurse or other clinical team member that is qualified and licensed to administer blood products. Administration date and time, will be entered into the case report form (CRF). The one (500 mL) or two (250 mL) plasma units collected from one or two donors will be considered one treatment dose.

a. The plasma will be administered at an infusion rate of no more than 150 mL/h and a standard test dose of 50 mL/hour for the 15 minutes or as per local procedures is mandatory.
b. If an AE develops during infusion, the infusion will be stopped and can be restarted as per investigator’s decision in consultation with the bedside physician.
    i. Most reactions to plasma are relatively minor (urticaria or volume overload) and the infusion can generally be continued. Infusion can generally be continued in cases of itching or urticarial reactions after pausing the transfusion, administering antihistamines, and observing the patient for worsening.
c. Severe allergic reactions such as, bronchospasm and hypotension, and will require discontinuation of the infusion.
d. Transfusion of each unit must be completed within 4 hours of removal from storage. The complete study product (either 1 x 500 mL or 2 x 250 mL) must be infused within 24 hours of randomization.
e. No medication or solution may be added to or infused through the same tubing simultaneously, unless the solution has been approved by Health Canada for this use (i.e. normal saline).
f. Participants must be under direct or remote observation during the first 15 minutes. Vital signs should be assessed at 15 minutes, midway through the infusion and at the end of the infusion.

The maximum tolerated dose of AFP has not been determined. The most likely adverse effect from the transfusion would be hypervolemia which may be treated with diuretics in symptomatic patients by the bedside physician. Any over-dosage needs to be reported as per section 9.10.2.

5.8. **Dose of CCP**

The dose of CCP will be approximately 500 mL. This will be administered as be either 1 unit of 500 mL or 2 units of 250 mL of apheresis CCP.

5.9. **Return and/or Destruction of Investigational Product**

After the study treatment period has ended or as appropriate over the course of the study, disposition of unused and used plasma will occur following site procedures in the study protocol. In the context of the COVID-19 pandemic, this may include disposal of the used plasma in the biohazard disposal in the patient room. The final disposition of the product will be confirmed in the Laboratory Information System used by the hospital Blood Bank and provided to the investigator team to be kept in the investigational product log. These logs will be verified periodically by study monitors. Any site found to use a study product on a non-CONCOR-1 trial patient will be

immediately suspended to trial enrollment and the local REB/IRB and regulatory authorities will be notified.

At the completion of the study, there will be a final reconciliation of investigational product shipped, used, and remaining. This reconciliation will be documented. Any discrepancies noted will be investigated, resolved, and documented prior to return or destruction of unused investigational product. Documentation of investigational product destroyed on site will be retained in the study files.

5.10. Blinding and Breaking the Blind

This is an open-label trial.

5.11. Investigational Product Accountability

The site PI is responsible for study product distribution and disposition and has ultimate responsibility for study product accountability. The site PI may delegate to the participating site's blood bank responsibility for study product accountability.

The participating site's blood bank will be responsible for maintaining complete records and documentation of study product receipt, accountability, dispensation, storage conditions, and final disposition of the study product(s).

All study product(s), whether administered or not, must be documented on the appropriate study product accountability record or dispensing log. The sponsor's monitoring staff will verify the participating site's study product accountability records and dispensing logs per the site monitoring plan.

5.12. Compliance

CCP will be transfused in the clinical area by the patient's nurse or physician, with oversight from the study team (site PI and/or site RA) who will not enter the room, thereby ensuring compliance with the investigational product and good practices in the context of the COVID-19 pandemic.

5.13. Prior and Concomitant Medication

A concomitant medication is any treatment received by the subject concomitantly to any investigational medicinal products (IMPs) or during study participation.

Concomitant medications in a hospitalized population change daily and are difficult to collect and attribute to success and failure of therapy and impact on safety. Therefore, only selected concomitant medications will be recorded in this trial. The list of medications will be assessed only from 7 days prior to enrolment to Day 30 or discharge, whichever comes first, and will be detailed in the study operations manual.

6. STUDY OUTCOMES

6.1. **Primary Outcome:**

The primary outcome is a composite of intubation or death at Day 30.

6.2. **Secondary Outcomes:**

- Time to intubation or death
- Ventilator-free days
- In-hospital death by Day 90
- Time to in-hospital death
- Death by Day 30
- Length of stay in ICU
- Length of stay in hospital
- Need for ECMO
- Need for renal replacement therapy
- Myocarditis
- Patient-reported outcome at Day 30
- Incremental cost per quality-adjusted life year (QALY)
- CPP transfusion-associated AEs
- Grade 3 and 4 SAEs
- Cumulative incidence of Grade 3 and 4 SAEs (CTCAE)

7. DURATION OF FOLLOW-UP

Subjects will be followed daily until hospital discharge or death. Patients discharged from hospital before Day 30 will be contacted by telephone on Day 30 ± 3 days to ascertain any AEs, vital status (dead/ alive), hospital readmission and need for mechanical ventilation after discharge. Patients with a prolonged hospital admission will be censored for the outcome of in-hospital death at Day 90.

For patients who are pregnant or who become aware that they are pregnant between the period of randomization and Day 30, we will record their expected date of delivery and we will contact the patient after delivery to obtain information regarding any maternal or fetal adverse outcomes.

8. DATA COLLECTION AND SCHEDULE OF EVENTS

The local study coordinator will collect all study data and record the data in the CRF (electronic and/or paper) as per local study procedures and requirements for each site.

8.1. **Data to be collected from the blood supplier**

De-identified data on the plasma donor will be obtained from the blood suppliers. For Canadian and Brazilian sites, this data will include unit information (such as ABO group, volume, antibody titre) and donor information (such as age, sex, symptom onset, severity, treatment, date of resolution) and will be sent via secure file transfer in accordance with institutional protocols. For US sites and the Israeli site, the data will include ABO group, volume, antibody titre (retrospectively) and donor information (such as age, sex, symptom onset, date of resolution) and will be sent via secure file transfer in accordance with institutional protocols.

8.2. **Subject Screening**

At each participating site, there will be a process developed to immediately identify patients admitted to hospital with COVID-19. The local RA will then screen the patient for study eligibility, provide study details to the patient, and seek informed consent. Information from all patients approached for the study will be documented and logged using the screening study number including reasons for any exclusion. Once informed consent is obtained, a screening ABO blood group will be performed if one was not already performed during the patient's current hospital admission.

Once the ABO blood group testing has been performed, the remaining sample (discard) will be retained for future testing. Alternatively, an extra tube can be added to the routine blood draw for research.

8.3. **Collection of daily hospital data**

Baseline data will be collected on Day 1 (see Section 8.5).

During the index hospital admission, daily data will be collected until discharge or Day 30 from randomization, whichever comes first. For enrolled patients who are still in hospital beyond Day 30, the date of discharge or death will be recorded up until Day 90 at which point data collection will be censored. This information will be obtained by reviewing the medical chart, by telephone follow-up or, if feasible through, provincial/state or national databases.

If the patient has routine blood drawn for laboratory testing pre-randomization and between 24 hours and 5 days (sample closest to 48 hours will be selected in preference) post-randomization, the discard plasma/serum from that sample will be retrieved, aliquoted and frozen for future testing. Alternatively, an extra tube can be added to the routine blood draw for research.

A Schedule of Assessment Events is available below for reference.

Schedule of Assessment Events

			Follow up		
Visit	Screening	Baseline (D1)	D2 – D30[1] (or until discharge)	D30[2] (if patients discharged)	D90
Time Window (days)				+/- 3	+/- 7 days
Informed Consent/Assent	X				
Verify Eligibility Criteria	X				
Randomization		X			
CCP Administration [3]		X			
Demographics		X			
Co-morbidities		X			
COVID-19 History[4]		X			
Height and Weight		X			
Vital Signs		X			
SpO_2		X	X		
FiO_2		X	X		
COVID treatments		X	X	X	
Hospitalization Daily Assessment [5]		X	X		
Results of blood tests (done as per clinical need)		X	X[6]		
EQ-5D-5L[7]		X		X	
Vital Status (dead/ alive)			X	X	X
Adverse Event Review		X	X	X	
Discharge status [8]				X	
Mechanical Ventilation since discharge				X	
Viral load, cytokines and anti-CoV-2 titers[9]		X	X		

[1] Only collected if still hospitalized
[2] Phone call if subject is already discharged from the hospital
[3] Only for subject randomized to the convalescent plasma treatment
[4] Includes date of first symptoms; description of symptoms, and date of diagnostic test
[5] Mechanical ventilation, ECMO, renal replacement therapy or myocarditis
[6] Daily from Day 1-7, then Day 14 +/- 3 days
[7] EQ-5D-5L to be collected on Day 1 after consent and prior to CCP administration and on Day 30 regardless of hospitalization status
[8] Discharge or hospital status (admitted, home, local hospital, longterm care facility)
[9] To be performed using frozen leftover sample or extra tube collected once at baseline and once during follow-up between 24h and 5 days post-randomization for both arms

8.4. **Baseline/Enrollment (Study Day 1)**

Procedures/Assessments/Evaluations in this section will be completed after consent has been obtained, eligibility has been confirmed and the subject has been randomized. These do not need to be performed in the order listed below, but the administration of CCP must be the last procedure to be completed.

1- Demographics
2- Co-morbidities
3- Smoking status and pack-years for smokers
4- COVID-19 history (symptom onset, date of test, results of test)
5- Admission date
6- Oxygen saturation and PaO_2 (where available)
7- FiO2 and oxygen requirement (nasal prongs, oxygen masks, high-flow nasal cannula, noninvasive positive pressure ventilation)
8- Vital signs (temperature, pulse, respiratory rate, height, weight, and blood pressure)
9- Lab values
10- Patient-reported outcome instrument EQl-5D-5L [49]
11- AEs since screening (if screening was not completed on the same day)
12- Concomitant medication review (if screening was not completed on the same day)
13- COVID-19 concomitant treatments including co-enrolment in another clinical trial
14- Administration of investigational product (according to study arm)

8.5. **Collection of daily hospital data (Study Day 2 until discharge, death or Day 30 post randomization).**

The following data and procedures will be collected daily.

1- Oxygen saturation and FiO_2
2- Mechanical ventilation requirement (via endotracheal tube or tracheostomy tube)
3- Admission to ICU
4- ECMO requirement
5- Renal replacement therapy requirement
6- Myocarditis
7- COVID-19 concomitant treatments
8- Blood product and blood transfusion information
9- AEs
10- Concomitant medication review
11- Chest x-ray and CT chest results
12- Discharge status (home, transfer to another facility, palliative discharge) - collected from the hospital chart

8.6. **Laboratory Test Results**

The following laboratory testing results will be recorded only if ordered by the bedside team at baseline (most recent results from day of randomization or previous 2 days), daily until Day 7, and at Day 14 (+/- 3 days).

1. CBC
2. INR, PT, aPTT, Fibrinogen, D-dimer
3. ALT, ALP, Bilirubin (total)
4. Albumin
5. Creatinine
6. LD
7. CK, C-Reactive Protein, Total Protein
8. Blood gas results (PaO_2, $PaCO_2$)
9. Ferritin
10. Total CO2
11. Troponin I HS
12. Lactate

8.7. **Discharge**

1. Hospital / Discharge status (home/local hospital/longterm care facility)
2. Oxygen requirement
3. Need for mechanical ventilation
4. AEs

8.8. **Day 30**

1. Vital status (dead or alive)
2. Requirement for mechanical ventilation since discharge
3. Oxygen requirement
4. Hospital status (home/local hospital/longterm care facility)
5. Readmission to hospital (if discharged home/longterm care facility)
6. EQ-5D-5L
7. AEs

8.9. **Day 90 follow-up**

Occurrence and date of hospital death or discharge (for participants still hospitalized at Day 30 only).

8.10. **Protocol Deviations**

A protocol deviation is any noncompliance with the clinical trial protocol, any process that is noted in the protocol and refers to details in the protocol-specific operations manual or Good Clinical Practice (GCP) requirements or any critical study procedures with specific instructions in ancillary documents referenced in the protocol such as a protocol-specific operations manual.

It is the responsibility of the investigator to ensure that only investigative procedures, as outlined in this protocol are performed on study participants; the occurrence of deviations from the protocol or SOPs are limited; and compliance with the regulations is maintained. Planned deviations from the protocol must not be implemented without prior agreement from the sponsor and approval from the local REB/ethics committee, as required, unless to eliminate an immediate hazard to a participant.

Planned or unplanned deviations may occur on the part of the participant, the investigator, or study research team. In resolution to a deviation, corrective/preventative actions are to be developed and implemented in a timely manner. Protocol deviations will be documented and reported as required and assessed where necessary during analysis. All individual protocol deviations will be addressed in subject study records.

It is the responsibility of the site PI and personnel to use continuous vigilance to identify and report deviations within five working days of identification of the protocol deviation, or within five working days of the scheduled protocol-required activity. All deviations must be promptly reported per the protocol deviation reporting procedures. Protocol deviations must be sent to the local REB per their guidelines. The site PI and personnel are responsible for knowing and adhering to their REB requirements. A completed copy of the Protocol Deviation Form must be maintained in the Essential Documents, as well as in the subject's chart if the deviation is subject specific.

9. SAFETY VARIABLES

9.1. Adverse Event

An AE is any untoward medical occurrence that occurs in a patient or clinical investigation subject administered a pharmaceutical product, and which does not necessarily have a causal relationship with the treatment assignment. An AE can therefore be any unfavorable and unintended sign (including abnormal laboratory finding), symptom, or disease temporally associated with the use of an investigational product, whether or not considered related to the product.

Because this is a trial focusing on a critically ill population where serious adverse events and death are expected to occur frequently, AE reporting will follow recommendations set out for trials in this population by Cook and colleagues[50]. During this trial, investigators will need to document all AEs that are definitely or probably related to the intervention (defined in 9.4.1), and events that are grade 3 or higher for all other AEs.

These will be documented on the paper CRF and in the electronic CRF (e-CRF) with the maintenance of all source documentation in the patient research file. Concomitant illnesses, which existed before entry into the study, will not be considered AEs unless they unexpectedly worsen during the treatment period. AEs must be documented regardless of the source of identification (for example physical examination, laboratory assessment, reported by subject).

9.2. Serious Adverse Event

A SAE is generally defined as any untoward medical occurrence that at any dose:

1. Results in death;
2. Is life-threatening; this means that the subject is at risk of death at the time of the event; it does not mean that the event hypothetically might have caused death if it was more severe;
3. Requires hospitalization (overnight or longer) or prolongation of existing hospitalization or invasive procedure;
4. Results in persistent or significant disability or incapacity;
5. Results in congenital anomaly or birth defect;
6. Is not be immediately life-threatening or result in death or hospitalization but may jeopardize the subject or require intervention to prevent one of the above outcomes.

Events associated with hospitalization for the following will **not** be considered as an SAE:

1. Evaluation or treatment of a pre-existing and non-exacerbating condition as long as the condition is associated with the hospitalization (e.g. hospitalization for investigation of a benign disorder);

2. Existed prior to the subject's entry into the study and has been recorded in the subject's disease/medical history as documented in the e-CRF;

3. Has not worsened in severity or frequency during the subject's exposure to investigational product;
4. Has not required a change in treatment management during the subject's exposure to the investigational product;
5. Elective or pre-planned treatment of a pre-existing and non-exacerbating condition.

In the specific context of a clinical trial focussing on a critically ill population, the definition of SAE also excludes:

a. AEs that are part of the natural history of the primary disease process or expected complications of critical illness.
b. AEs that are already captured as study outcomes.

Medical and scientific judgment should be exercised in deciding whether an AEs is serious and whether expedited reporting is appropriate. Caution must also be exercised by the investigator when attributing death or prolonged hospitalization to a specific AE. All-cause mortality and hospitalization stay will be captured as study outcomes.

9.3. **Severity of Adverse Events**

The severity of each AE must be assessed by the Investigator and graded based on CTCAE v4.0. A clinical AE NOT identified elsewhere in the grading table will be graded using one of the following categories:

1. **Mild**: Mild symptoms causing no or minimal interference with usual social & functional activities with intervention not indicated
2. **Moderate**: Moderate symptoms causing greater than minimal interference with usual social & functional activities with intervention indicated
3. **Severe**: Severe symptoms causing inability to perform usual social and functional activities with intervention or hospitalization indicated
4. **Life-threatening:** Potentially life-threatening symptoms causing inability to perform basic self-care functions with intervention indicated to prevent permanent impairment, persistent disability, or death.
5. **Death:** The AE resulted in the subject's death.

9.4. **Causality of Adverse Event**

9.4.1. **Relation to investigational product**

The Investigator will assess the causality/relationship between the investigational product and the AE and record that assessment in the source documents and in the e-CRF. The most likely cause of an AE/SAE (for example disease under treatment, concomitant disease, concomitant medication, other) will be indicated in the e-CRF.

In the absence of a definite causal relationship, any adverse event that is considered to have a <u>reasonable</u> causal relationship with the investigational product should be classified as "probably related."

The causal relationship of the AE to investigational product will be described in terms of:

1. **Related. The AE:**
    1. Follows a clear temporal sequence from administration of the investigational product.
    2. Has no other possible explanations, such as the subject's clinical state, environmental or toxic factors or other therapies administrated to the subject.
    3. Disappears or decreases on cessation or reduction in dose of the investigational product.
    4. Follows a clear pattern of response to the investigational product.
    5. Reappears or worsens upon re-challenge.

2. **Probable. The AE:**
    1. Follows a reasonable temporal sequence from administration of the investigational product.
    2. Could not be reasonably explained by the subject's clinical state, environmental or toxic factors or other therapies administrated to the subject.
    3. Disappears or decreases on cessation or dose reduction of the investigational product.
    4. Follows a known pattern of response to the investigational product.
    5. Reappears or worsens upon re-challenge.

3. **Possible. The AE:**
    1. Follows a reasonable temporal sequence from administration of the investigational product.
    2. Could be reasonably explained by the subject's clinical state, environmental or toxic factors or other therapies administrated to the subject.
    3. Follows a known pattern of response to the investigational product.

4. **Unlikely. The AE:**
    1. Does not follow a reasonable temporal sequence from administration of the investigational product.
    2. Could be reasonably explained by the subject's clinical state, environmental or toxic factors or other therapies administrated to the subject.
    3. Does not follow a known pattern of response to the investigational product.
    4. Does not reappear or worsen upon re-challenge.

5. **Not related.**
    1. The AE does not meet the above criteria.
    2. There is sufficient information that the etiology of the AE is not related to the investigational product.

9.5. Recording Adverse Events occurring after the end of the study

Adverse events occurring after the end of the study should be reported to the Sponsor by the Investigator if the Investigator considers that there is a causal relationship with the investigational product.

9.6. Adverse Event Reporting

At each clinical evaluation, the Investigator will determine whether any AEs have occurred. AEs will be recorded in the AE page of the e-CRF as well as in the source documents.

Coding of AEs will be performed using MedDRA (https://www.meddra.org/). All AE reports in the e-CRF should contain the following information of the event: date of onset, severity, relationship to investigational product, outcome at Day 30, and whether the event is classified as serious or not.

9.6.1. Serious Adverse Event Reporting

SAEs that are unexpected and either definitively or probably related to the investigational products (Suspected Unexpected Serious Adverse Reactions – SUSARs) must be reported to the sponsor. SAEs that are expected and either definitively or probably related to the investigational product and non-CCP related SAEs that meet reporting criteria (unexpected in the context of COVID-19, critical illness, or a baseline medical condition; or not already reported as a study outcome) must also be reported to the sponsor.

In the event of a SAE, the Investigator must:

1. Enter all relevant information in the AE page of the eCRF.
2. Inform the Sponsor of the SAE via email or telephone within 96 hours of becoming aware of the SAE (or within 24 hours if SAE is unexpected and related to investigational product, see section 9.8).
3. Complete the SAE reporting form in the EDC.

The minimum information required for an initial report is:

1- Name of person sending the report (that is name and address of Investigator);
2- Confirmation that the patient was in the active treatment arm;
3- Subject identification (screening number, NOT the subject's name);
4- Protocol number;
5- Description of the SAE including a comprehensive verbatim term;

6- Event onset date;
7- Causality assessment, if possible.

However, as far as possible, all points in the SAE pages in the e-CRF and on the SAE Report Form should be covered in the initial report.

SAE update reports should be submitted to the Sponsor when additional relevant information becomes available.

Suspected unexpected serious adverse reactions (SUSARS) will be submitted by the Sponsor or its representative to the Regulatory Authorities according to local guidelines.

9.6.2. Sponsor Reporting of SUSARs: Notifying Health Canada

The regulatory sponsor (or its delegate) is responsible for reporting SUSARs to regulatory authorities in accordance with local expedited reporting requirements and timelines. In addition, the Sponsor will complete the Adverse Drug Reaction Expedited Reporting Summary Form and submit this form to the appropriate Health Canada directorate.

Any unexpected fatal or life-threatening suspected adverse reaction will be reported to the regulatory authority as soon as possible, but in no case later than 7 calendar days after the sponsor's initial receipt of the information. If the event is not fatal or life-threatening, the safety report will be submitted within 15 calendar days after the sponsor determines that the information qualifies for reporting. Relevant follow up information to safety report will be submitted as soon as the information is available. Upon request from regulatory authority, the sponsor will submit any additional data or information that the agency deems necessary, as soon as possible, but in no case later than 15 calendar days after receiving the request.

9.6.3. Reporting of Serious Adverse Events to the FDA

The U.S. site regulatory sponsor/sponsor representative is responsible for notifying the FDA of any suspected adverse reaction to study treatment that is both serious and unexpected to the FDA. The event will be filed on the FDA Form 3500A and submitted as soon as possible but no later than 7 calendar days after the initial receipt of the information to CBER.

All transfusion-related fatalities must be reported as soon as possible to Food and Drug Administration (FDA), Center for Biologics Evaluation and Research (CBER), Office of Compliance and Biologics Quality (OCBQ) after confirming a complication of blood collection or transfusion to be fatal. The collecting facility is to report donor fatalities, and the compatibility testing facility is to report recipient fatalities. The regulation also requires the reporting facility to submit a report of the investigation within 7 days after the fatality.

9.6.4. **Reporting of Serious Adverse Events to** Agência Nacional de Vigilância Sanitária (ANVISA-Brazil)

The regulatory sponsor/sponsor representative (or its delegate) is responsible for reporting Adverse Events to national regulatory authorities (ANVISA) in accordance with local expedited reporting requirements and timelines. Relevant follow up information to safety report will be submitted as soon as the information is available. Upon request from regulatory authority, the sponsor will submit any additional data or information that the agency deems necessary, as soon as possible, but in no case later than 15 calendar days after receiving the request.

The sponsor will also notify the local IRB and CONEP, within a maximum period of 24 hours, of any serious adverse event, suspected of having been caused by the proposed treatment (including death).

9.6.5. **Reporting of Serious Adverse Events to the Meir Medical Centre Ethics Committee and Israeli Ministry of Health**

The Israeli site regulatory sponsor/sponsor representative is responsible for notifying the Meir Medical Center Ethics Committee and to the Israeli Ministry of Health when considered necessary by the former body, of any suspected adverse reaction to study treatment that is both serious and unexpected. The event will be reported as soon as possible but no later than 7 calendar days after the initial receipt of the information.

9.6.6. **Sponsor Reporting of SUSARs: Notifying Sites**

The regulatory sponsor is responsible for distributing expedited reports of SUSARs to each investigator for submission to local Ethics Committees within 15 days of sponsor awareness.

U.S. site regulatory sponsor/sponsor representative is responsible for submitting all SUSARs that meet criteria to the FDA.

Brazilian site regulatory sponsor/sponsor representative is responsible for submitting all SUSARs that meet criteria to ANVISA.

9.7. **Type and Duration of Follow-up for Adverse Events**

AE collection will begin at randomization and end at Day 30.Unresolved events at Day 30 will be marked as “ongoing at Day 30.”

Adverse events will be monitored until Day 30. For participants still hospitalized at Day 30, hospital mortality will be followed up on Day 90.

9.8. **Reporting and Entry Timelines**

Study investigators will report SAEs to the sponsor within the following timelines:

- All serious and unexpected events deemed to be related to investigational product (suspected unexpected serious adverse reaction (SUSAR)) will be recorded and reported to the sponsor within 24 hours of site awareness.
- All other SAEs will be reported to the sponsor within 96 hours of site awareness.

Adverse event information will be entered into the CRF in a timely manner and **no later than 7 days** from the time the investigator becomes aware of the event.

Serious adverse event information will be entered into the CRF in a timely manner/**within 96 hours** from the time the investigator becomes aware of the event (or 24h for SUSARs).

9.9. Reporting of Events Other than Serious Adverse Event

Even if none of the criteria for a SAE are fulfilled, any of the following events must be reported by the Investigator to the Sponsor within 24 hours from the time the Investigator is notified.

- Transfusion associated acute lung injury (TRALI)
- Transfusion associated circulatory overload (TACO)

9.10. Adverse Events to Convalescent Plasma Transfusion

AEs to CCP will be categorized using the ISBT definitions for diagnosis, severity and imputability. AEs to any other blood products that the patient receives during the hospitalization will reported as AEs as well as declared following normal procedures in place locally.

9.10.1. Pregnancy

The Sponsor has a responsibility to monitor the outcome of pregnancies where there has been maternal exposure to the investigational product. Pregnancy alone is not regarded as a contraindication to participation in the trial or as an AE unless there is a suspicion that the investigational product may have interfered with the effectiveness of a contraceptive medication. Elective abortions without complications should not be handled as AEs, unless they were therapeutic abortions (see below). Hospitalization for normal delivery of a healthy newborn will not be considered a SAE.

All pregnancies occurring in the CCP group must be reported by the Investigator to Sponsor on a separate CRF within 24 hours after becoming aware of the pregnancy. The Investigator must follow up and document the course and the outcome of all pregnancies even if the subject was discontinued from the study or if the study has finished. All outcomes of pregnancy must be reported by the Investigator to the Sponsor on a specific pregnancy CRF within 96 hours after she/he has gained knowledge of the normal delivery or elective abortion.

Any SAE related to investigational product that occurs during pregnancy (including SAEs occurring after last administration of investigational product) must be recorded in the SAE pages in the CRF (for example serious maternal complications, spontaneous or therapeutic abortion, ectopic

pregnancy, stillbirth, neonatal death, congenital anomaly, or birth defect) and reported within 96 hours in accordance with the procedure for reporting SAEs.

9.10.2. Treatment of Overdose of investigational product

Overdose is unlikely since only the required amount of CCP will be issued to a patient. In the unlikely event of inadvertent overdose due to human error, with or without associated AEs, the event will be reported to the Sponsor as per procedures outlined in the operations manual. Overdose will be reported to the sponsor within 24 hours of learning of the overdose. Any AEs associated with the overdose should be reported in the relevant AE/SAE sections in the CRF.

10. INDEPENDENT COMMITTEES

10.1. Independent Data Safety Monitoring Committee

Safety oversight will be conducted by an independent data safety monitoring committee (IDSMC) that is an independent group of experts that monitors subject safety. The IDSMC members will be separate and independent of study personnel participating in this trial and should not have scientific, financial or other conflict of interest related to this trial.

The IDSMC will consist of members with appropriate expertise to contribute to the interpretation of the data from this trial. The IDSMC should be as broadly informed as possible regarding emerging evidence from related studies as well as from the conduct of the Master Protocol from which this was adapted. The IDSMC will operate under the guidelines of a charter that will be written at the organizational meeting of the IDSMBC. The IDSMC will review SAEs on a regular basis and ad hoc during the trial according to the IDSMC charter.

The study will not stop enrolment awaiting these IDSMC reviews, though the IDSMC may recommend temporary or permanent cessation of enrolment based on their safety reviews.

Additional data may be requested by the IDSMC, and interim statistical reports may be generated as deemed necessary and appropriate. The IDSMC may receive data in aggregate and presented by treatment arm. The IDSMC may also be provided with expected and observed rates of the expected AEs in an unblinded fashion and may request the treatment assignment be unblinded for an individual subject if required for safety assessment. The IDSMC will review grouped and unblinded data in closed sessions only. As an outcome of each review/meeting, the IDSMC will make a recommendation as to the advisability of proceeding with study interventions (as applicable), and to continue, modify, or terminate this trial. Criteria for stopping the trial due to harm are left to the judgement and discretion of the IDSMC as outlined in the charter of the IDSMC.

10.2. Community Advisory Committee

A Community Advisory Committee (CAC) will be established to bring a diversity of perspectives based on relevant lived experiences and socio-demographic characteristics (e.g. age, sex/gender, education, ethnicity) to the CONCOR-1 Trial. The CAC will consist of 7-9 selected individuals, in addition to a CONCOR-1 Steering Committee Member, who will sit on the CAC, and act as a liaison to the overall trial, helping the CAC members to facilitate their discussions. Members from the patient community will be invited to apply to join the CAC. Additionally, the CAC will have representatives that are donors of plasma, recipients of plasma/COVID-19 patients and family members or caregivers of recipients/donors. Applicants to the CAC will be selected based on their Various Geographical locations, their status as Marginalized Individuals, Indigenous Individuals, Disabled Peoples/ Peoples with existing illnesses, Participants in the trial, and Severity of sickness with COVID-19. Broad recruitment of participating sites that include both academic and community hospitals in Canada will maximize access to the trial for patients. Individuals will be recruited to be a part of the CAC through various community engagement networks, such as the SPOR Network and the McMaster University Office of Community Engagement.

Advisors from marginalized populations affected by the pandemic will help advocate for access to the trial and to study results for people who are often excluded from research such as residents of long term care facilities, African American and Indigenous people.[51,52] Goals of this committee are 1) to maximize the relevance of the research for the community; 2) to advise on equitable distribution of CCP; 3) to provide input on equitable recruitment of trial participants; 4) to provide feedback on analyses including awareness of the effects of social determinants, 5) to enhance effective dissemination of study results; and, 6) to create a space of inclusivity for participants to engage as a community and share their experiences with COVID-19.[52,53] Creation of the CAC will build capacity for future citizen engagement initiatives within the Canadian Transfusion Research Network.

11. SITE MONITORING, AUDITING AND INSPECTING

11.1. Informed Consent

Consent will be obtained for donors through Canadian Blood Services and Héma-Québec (separate REB application at both Canadian Blood Services and Héma-Québec). In the U.S., consent will be obtained by the New York Blood Center through a separate IRB application, as well as by Hemorio for the Brazilian sites and by Magen David Adom National Blood Services for the Israeli site.

The Investigator is responsible for ensuring informed consent is obtained from each hospitalized participant.

In obtaining and documenting informed consent, the investigator should comply with the applicable regulatory requirement(s), should adhere to GCP and to the ethical principles that have their origin in the Declaration of Helsinki. Prior to the beginning of the trial, the Investigator will have their applicable REB/Institutional Review Board (IRB)/independent ethics committee's (IEC) written approval/favourable opinion of the written informed consent form and any other information to be provided to subjects.

Documentation of informed consent must be dated and retained by the Investigator as part of the study records. The Investigator will not undertake any investigation specifically required only for the clinical study until valid consent has been obtained. Sites must follow local informed consent policies and guidelines.

Subjects will be informed of the nature of the study, its aim, its possible risks and restrictions, its duration, and any compensation that they may receive. The trial and participant responsibilities will be explained during the informed consent discussion prior to study enrollment, and each subject must be informed that their participation in the study is voluntary and that they may withdraw from the study at any time. The subject or their legally authorized representative (LAR) must read or fully understand the study/ICF and have an opportunity to ask questions before they provide consent to participate. Where applicable, the participant may be asked to sign a paper copy of the consent, prior to discharge, where it is safe to do so, for both the patient and health care team. A copy of the signed informed consent or documentation of remote consent must be made a part of the subject's medical file and a copy provided to the patient or their legally authorized representative.

If a protocol amendment is required, the ICF may need to be revised to reflect the changes to the protocol or safety profile. If the consent form is revised, it must be reviewed and approved by the appropriate REB/IRB/IEC, and may need to be re-signed by the subjects or LAR previously enrolled in the study as well as those currently enrolled in the study before Day 30. Sites should follow local policies and guidelines.

The Investigator should inform the subject's primary physician about participation in the clinical study where applicable.

11.2. Data Quality Assurance

The Sponsor or Sponsor's designee will conduct a site visit or a site phone contact to verify the qualification of each Investigator, review the site facilities, and inform the Investigator of her/his responsibilities and the required procedures for ensuring adequate site selection and correct documentation. On-site visits may be restricted due to site local visitor and research personnel restrictions. Where necessary, virtual site visits will continue to ensure compliance with the study protocol and procedures.

11.2.1. Database Management and Quality Control

All data generated by the site personnel will be captured electronically at each study centre using e-CRFs. Data from external sources (such as laboratory data) will be inputted manually.

Once clinical data are entered and validated in the e-CRF, they are transmitted and recorded by the central server in the central database. Computerized edit-checks will be developed in addition to manual review to detect any discrepancies and to ensure consistency of the data.

The appropriate staff at the study site will answer queries sent to the site. The reason for changes, the name of the person who performed the changes, together with the time and date will be automatically recorded by the Electronic Data Capture (EDC). An electronic audit trail system will be used to track all data changes in the database subsequent to the first data entry.

If additional corrections are needed, the responsible monitor, data manager or the Sponsor will raise a query in the EDC application. Appropriate feedback to the study staff or other corrective measures will be undertaken if any missing data, inconsistent data, outlier data and potential protocol deviations are identified during routine remote data review.

11.3. Case Report Forms and Source Documentation

All data obtained during this study must be entered in the e-CRFs in a timely fashion (within 30 days). All source documents from which e-CRF entries are derived should be filed in the patient's study chart.

Site personnel will receive training on the e-CRF and the specific procedures to be used for data entry and query resolution.

11.3.1. Data Collection

The Investigators (and appropriately authorized staff) will be given access to an online web-based EDC system. This system is specifically designed for the collection of the clinical data in electronic format. Access and rights to the EDC system will be carefully controlled and configured according to each individual's role throughout the study. In general, only the Investigator and authorized staff will be able to enter data and make corrections in the e-CRFs.

The e-CRF should be completed for each subject for whom the study-specific ICF was obtained and should reflect the latest observations on the subject participating in the study.

Therefore, the e-CRFs are to be completed as soon as possible after the data is collected (with a maximum delay of 30 days). The Investigator must verify that all data entries in the e-CRF are accurate and correct. If some assessments cannot be done, or if certain information is unavailable, not applicable or unknown, the Investigator should indicate this in the e-CRF.

After completion, the Investigator will be required to validate and electronically sign-off the clinical data recorded in each e-CRF.

Information concerning investigational product dispensation to the subject will be tracked in the e-CRF.

11.4. Access to Source Data

11.4.1. Remote Monitoring

Considering the current context of pandemic, during the study, a site monitor will conduct remote monitoring site visits to review protocol compliance, compare e-CRF entries and individual subject's medical records (source verification), assess investigational product accountability, and ensure that the study is being conducted according to ethical and pertinent regulatory requirements. The e-CRF entries will be verified with source documentation. The review of medical records will be performed in a manner to ensure that subject confidentiality is maintained.

Checking of the e-CRF entries for completeness and clarity, and cross-checking with source documents, will be required to monitor the progress of the study. Access to source data will be required for the monitoring activities. Source documents will be scanned and sent through a secure email. In both cases, the copies received will be destroyed by the monitor after their review. All source documents will be anonymized (names will be removed and replaced with the subject's study ID number at the site level) prior to being sent to the study monitor.

11.4.2. Inspection and Audit Procedures

The Sponsor or its representative may conduct audits at the investigative sites including, but not limited to, investigational product supply, presence of required documents, the informed consent process, and comparison of e-CRFs with source documents. All medical records (progress notes) must be available for audit. The Investigator agrees to participate in audits conducted at a convenient time in a reasonable manner.

Moreover, local Regulatory Authorities may wish to carry out such source data checks and/or on-site inspections. Direct access to source data will be required for these inspections; they will be carried out giving due consideration to data protection and medical confidentiality.

11.5. **Data Processing**

All data will be entered by authorized site personnel into the e-CRF. The data-review and data-handling document will include specifications for consistency and plausibility checks on data and will also include data-handling rules for obvious data errors. Queries/inconsistencies will be sent to the study Investigators and monitors for resolution. The database will be updated on the basis of validated/signed corrections.

Previous and concomitant medications will be coded using the WHO Drug Reference List, which employs the Anatomical Therapeutical Chemical classification system. Disease and Medical histories/current medical conditions and AEs will be coded using MedDRA. The CTCAE v4.0 will be used to support investigator severity grading. The versions of the coding dictionaries will be provided in the Clinical Study Report.

11.6. **Archiving Study Records**

All data derived from the study will remain the property of the Sponsor. Records must be retained in accordance with the current International Conference on Harmonization (ICH) Guidelines on GCP. All essential study documents including records of subjects, source documents, e-CRFs and investigational product inventory must be kept in a study specific file. The Investigator site files must be retained by the Investigator at least 25 years after the completion of the trial.

Subjects' medical records must be kept for the maximum period permitted by the hospital, institution or private practice.

The Investigator will not discard any relevant records to this study without prior written permission from the Sponsor. The Investigator shall notify the Sponsor in writing of their intent to destroy all such material. The Sponsor shall have 30 days to respond to the Investigator's notice and the Sponsor shall have a further opportunity to retain such materials at the Sponsor's expense.

The Investigator shall take responsibility for maintaining adequate and accurate source documents (hard copy or electronic) of all observations and data generated during this study. Such documentation is subject to inspection by the Sponsor, its representatives and Regulatory Authorities. If an Investigator moves, withdraws from the study or retires, the responsibility for maintaining the records may be transferred to another person who will accept responsibility. Notice of transfer must be made to and agreed by the Sponsor.

11.7. **Good Clinical Practice**

The procedures set out in this study protocol are designed to ensure that the Sponsor and Investigators abide by the principles of the ICH-GCP Guidelines, and of the Declaration of Helsinki (**Appendix 1**). The study will also be carried out in keeping with local legal requirements.

11.8. Protocol Approval and Amendment

Before the start of the study, the study protocol and/or other relevant documents will be reviewed and approved by the REB/IRB/IEC/Regulatory Authorities, in accordance with local legal requirements. The Sponsor must ensure that all ethical and legal requirements have been met before the first subject is enrolled in the study.

This protocol is to be followed exactly. Any change to the protocol must be handled as a protocol amendment. Any potential amendment must be approved by the Sponsor. A written amendment must be submitted to the appropriate Regulatory Authorities and to the REB/IRB/IECs assuming this responsibility. The Investigator must await REB/IRB/IEC approval of protocol amendments before implementing the changes, except where necessary to eliminate apparent immediate hazard to subjects.

All substantial amendments to the protocol must be approved in writing by both the appropriate Regulatory Authorities and the REB/IRB/IEC, except for administrative amendments, which require notification or protocol addendums but not written approval or modifications that are subject to the Health Canada interim order for clinical trials on COVID-19 issued June 2nd 2020. Once approved, the protocol amendment will be distributed to all recipients of the original protocol, with instructions to append the amendment to the protocol. If, in the judgment of the local REB/IRB/IEC, the Investigator and/or Sponsor, the protocol amendment alters the study design, procedures and/or increases the potential risk to the subject, the currently approved written ICF will require modifications.

The modified ICF must also be reviewed and approved by the Sponsor, appropriate Regulatory Authorities, and the REB/IRB/IEC. In such cases, repeat informed consent must be obtained from the enrolled subjects or parents/guardians before participation continues.

11.9. Duration of the Study

For an individual subject, study ends for a subject 89 days after randomization (except for pregnant women on the CCP arm, which should be followed until pregnancy outcome is known). The overall study will end when the last randomized subject has reached the Day 90 time point. The inclusion of new participants will be determined based on new data and clinical needs according to the evolution of the pandemic. The planned study duration is 1 year.

11.10. Premature Termination of the Study

If the Investigator, the Sponsor, or the Medical Monitor becomes aware of conditions or events that suggest a possible hazard to subjects if the study continues, the study may be terminated after appropriate consultation between the relevant parties. The study may also be terminated early at the Sponsor's discretion in the absence of such a finding.

Conditions that may warrant termination include, but are not limited to:

1. Concern regarding an unacceptable risk to subjects
2. Results of interim analysis demonstrate superiority

3. Results of the interim analysis for the revised sample size makes the resultant study infeasible due to the large number of patients required
4. Insufficient compliance to protocol requirements
5. Data that are not sufficiently complete and/or are not evaluable
6. Regulatory authorities recommend termination of the study
7. Novel scientific data on the efficacy or safety of COVID-19 convalescent plasma is published during the conduct of the trial that raise concerns regarding the safety of CCP

In terminating the study, the Sponsor and the Investigators will ensure that adequate consideration is given to the protection of the subjects' interests.

11.11. Confidentiality

All information obtained during the conduct of the study with respect to the subject's state of health will be regarded as confidential. For disclosure of any such information, an agreement will be obtained in writing. The Investigator must ensure that each subject's anonymity is maintained. On e-CRFs and other documents transmitted to the Sponsor or its representatives, subjects must not be identified by name. Instead, subjects will only be known by the unique subject screening number allocated to them in order to ensure confidentiality on all study documentation. Subjects will retain this unique number throughout the study. The Investigator will keep a separate log of these codes.

In order to comply with government regulatory guidelines and to ensure subject safety, it may be necessary for the Sponsor and its representative, the REB/IRB/IEC or Regulatory Authorities to review subjects' medical records as they relate to this study. Only the subject's unique number in the e-CRFs will identify her/him, but their full names may be made known to a Regulatory Authority or other authorized government or health care officials, if necessary, and to personnel designated by the Sponsor.

Documents that are not for submission to the Sponsor (for example consent forms) will be maintained by the Investigator in strict confidence, except to the extent necessary to allow monitoring and auditing by the Sponsor and inspection by Regulatory Authorities. No documents identifying subjects by name will leave the investigative site and subject identity will remain confidential in all publications related to the study.

11.12. Publication Policy

By signing the study protocol, the Investigator agrees with the use of results of the study for the purposes of national and international registration, publication and information for medical and pharmaceutical professionals. If necessary, Regulatory Authorities will be notified of the Investigator's name, address, qualifications and extent of involvement. The Investigator shall not publish any data (poster, abstract, paper, etc.) without having consulted with the Sponsor in advance and having received a written approval for such a publication. The sponsor-investigator will be responsible for publication of the data.

The sponsor-investigator must commit to publish and disseminate the study results in a timely manner without excessive restriction, regardless of whether the initial study hypothesis is confirmed or not.

11.13. **Critical Documents**

Before the Investigator starts the trial, the following documents must be provided to the site from the sponsor:

1. Regulatory approval (Health Canada NOL)(FDA in U.S. site) (CONEP in Brazil) (Meir Medical Centre Helsinki Committee in Israel);
2. Protocol, any substantial amendments;
3. Subject information/informed consent form;
4. Any other written information to be provided to the subject;
5. Subject recruitment procedures;
6. Templates for Delegation of Authority, CTSI form, Qualified Investigator Form and Health Canada REB Attestation where applicable.
7. Study procedures documents: SOPs/Operations Manual, eCRF system setup.
8. SAE reporting procedure for the study

These documents must be collected from site in order to begin recruitment and subject enrollment:

1. Curricula vitae of Investigator and Sub-investigator(s) (current, dated and signed);
2. Favourable opinion from REB/IRB/IEC clearly identifying the documents reviewed;
3. Subject recruitment procedures that have not been identified by the protocol;
4. List of REB/IRB/IEC members and constitution;
5. Signed and dated agreement on the final protocol;
6. Signed and dated agreement on any substantial amendment(s), if applicable;
7. Copy of REB/IRB/IEC approved subject information/informed consent form;
8. Written information/advertisement;
9. Receipt of the Circular of Information/*Notice d'accompagnement* signed by Investigator (where applicable);
10. Laboratory certification and normal ranges;
11. Financial agreement(s).
12. Copy of completed Delegation of Authority

11.14. **Clinical Study Report**

Final clinical study reports will be prepared according to the ICH guideline on Structure and Contents of Clinical Study Reports. It will be prepared regardless of whether the study is completed or prematurely terminated. The Sponsor will provide each Investigator with a copy of the final report for retention.

12. STATISTICAL CONSIDERATIONS

12.1. Sample Size Considerations

Assuming a baseline risk of intubation or death by Day 30 of 30% in hospitalized patients with standard of care, a sample size of 1200 (800 in the CCP arm, and 400 in the standard of care arm) would provide 80% power to detect a relative risk reduction of 25% with CCP therapy using a 2-tailed test at level $\alpha = 0.05$. This assumes no patient is lost to follow-up. Lost follow-up is expected to be minimal.

There is uncertainty about the intubation and mortality rate that will be observed in this study due to possibly evolving profile of COVID-19 patients admitted hospital over the course of the pandemic. To address this, a blinded sample-size re-estimation will take place when the primary outcome is available for 50% of the target sample. Sample size will only be adjusted if the estimated event rate is different than the originally specified event rate to a sufficient extent that the study is not expected to achieve the desired nominal power for the test of the primary outcome.

12.2. Planned Interim Analyses

A single interim analysis is planned when information on intubation or death at Day 30 is available for 50% of the target sample. An O'Brien-Fleming stopping rule will be employed.[44]

The IDSMC may recommend early termination or modification of the protocol only when there is clear and substantial evidence of a treatment difference. More details about the interim analyses are described below as well as a separate guidance document for the IDSMC.

12.2.1. Safety Review

Safety analyses will evaluate serious AEs by treatment. The unblinded independent statistician will prepare these reports for review by the IDSMC. The IDSMC will review data on the occurrence of all reportable AEs at each of their meetings to ensure there are not significant numbers of unexpected AEs (AEs that do not fit with the known course of COVID-19, based on emerging data from the published and unpublished literature). If there is a significant number of unexpected AEs, the IDSMC will be asked to review unblinded safety data in an ad hoc meeting. Regular safety reports will be created to provide descriptive statistics on the occurrence of AEs to the IDSMC. The IDSMC may request safety data in a blinded or unblinded fashion as per their discretion.

12.2.2. Efficacy Review

A single interim analysis is planned when information on the primary outcome (composite of intubation or death at Day 30) are available for 50% of the target sample. The Lan-DeMets spending function[54] analog of the O'Brien-Fleming boundaries[44] will be used to monitor the primary endpoint as a guide for the DSMB for an overall two-sided type-I error rate of 0.05. Conditional power will be presented as an additional guide to the DSMB. Conditional power allows

computation of the probability of obtaining a statistically significant result by the end of the trial given the data accumulated thus far, incorporating a hypothesized treatment effect (e.g., the treatment effect assumed for sample size determination) thereafter. If conditional power is less than 20% under the original trial assumptions, consideration could be given to stopping the trial. An unblinded independent statistician will prepare the reports for IDSMC to review when making their recommendations. An IDSMC charter will further describe procedures and membership. An additional document on statistical methods related to the interim analyses will be provided to the IDSMC prior to their first meeting.

An additional document on statistical issues related to monitoring will be provided to the IDSMC prior to interim analyses.

12.3. **Stopping Rules**

If there are a concerning number of unexpected AEs, the IDSMC will be asked to review safety data in an ad hoc meeting. This study may be prematurely terminated if there is reasonable cause, including but not limited to: determination of unexpected, significant, or unacceptable risk to subjects; results of interim analysis; insufficient compliance to protocol requirements; data that are not sufficiently complete and/or not evaluable. If the study is prematurely terminated, the site Qualified Investigator will promptly inform study subjects and the REB as applicable. The site PI will assure appropriate follow-up for the subjects, as necessary. The sponsor will notify regulatory authorities as applicable. If there are a concerning number of unexpected AEs, the IDSMC will be asked to review all safety data in an ad hoc meeting.

This study may be prematurely terminated by the IDSMC and/or co-principal investigators and/or Sponsor if there is sufficient reasonable cause (see section 11.10)

12.4. **Final Analysis Plan**

The primary analysis will be based on the intention-to-treat population which will include data from all individuals who have been randomized. Outcomes will be attributed to the arm to which individuals were randomized irrespective of whether they received the planned intervention (e.g. plasma from a convalescent COVID-19 donor).

A per protocol analysis will also be performed. The per protocol sample will be comprised of eligible individuals who are randomized and are treated according to the protocol. In this trial, patients will be excluded from the per protocol analysis if any of the following protocol violations occur:

-patient received less than two units of CCP

-transfusion of second unit of CCP completed more than 24 hours after randomization (special consideration will be given for instances where 1 x 500 mL unit was given)

-patient was randomized to SOC but received CCP

-patient was randomized despite being ineligible for the trial

12.4.1. **Primary Efficacy Endpoint**

Intubation or death at Day 30 is the primary endpoint.

Given the fact that the times from admission to discharge or death are expected to be relatively short, we are using a binary response indicating intubation or death at Day 30. The primary analysis will therefore be based on a 2-sided Wald test of the null hypothesis that probability of intubation or death is the same among individuals receiving CP or standard of care. The relative risk reduction for death (CCP versus standard of care) will be computed along with a 95% confidence interval.

Efforts to minimize loss-to-follow-up will be considerable and we are expected to have complete data on individuals for the primary outcome.

Sensitivity analyses will evaluate the impact of making different assumptions about missing data for secondary outcomes. These sensitivity analyses will be fully defined in the statistical analysis plan (SAP).

12.4.2. **Secondary Endpoints**

A cause-specific Cox regression model will be fitted for the intubation-free survival time defined as the first of the times of intubation or death with censoring at discharge or 30 days whichever comes first. A hazard ratio and 95% confidence interval will be computed from this fitted model.

Cumulative incidence functions will be estimated and plotted for each treatment arm for the time to intubation or death.

The restricted mean number of ventilator-free days over the first 30 days will be compared between the two groups based on a nonparametric tests.

The time to in-hospital death will be analyzed using a cause-specific Cox regression model treating the minimum of the time of discharge and 90 days as the censoring time. A hazard ratio and 95% confidence interval will be computed from this fitted model. Cumulative incidence functions will be estimated and plotted to estimate the proportion of patients who died during the initial hospitalization up to 90 days from randomization.

Finally, the survival status of individuals will be recorded at day 30 and the proportion of individuals who have died will be computed for each arm with a two-sample Wald test carried out based on the proportions.

Length of hospitalization over 90 days and length of stay in the ICU over 30 days will be modeled using a Cox regression without any distinction between reasons for discharge.

The proportion of patients needing ECMO over 30 days will be compared between the two arms based on a two-sided test of the difference between proportions.

The proportion of patients needing renal replacement therapy over the first 30 days will be compared between the two arms based on a Wald test.

The proportion of patients developing myocarditis over the first 30 days will be compared between the two arms based on a Wald test.

The change in EQ-5D-5L utility value from baseline will be compared between the two arms.

Incremental cost per quality-adjusted life year between the CCP and standard care over the 30-day time horizon.

No multiplicity adjustments will be implemented for the secondary analyses. Procedures for addressing missing data will be described in the SAP.

12.4.3. Safety Analysis

Safety endpoints include death through Day 30, SAEs, and severe AEs. These events will be analyzed univariately and as a composite endpoint. Time-to-event methods will be used for death and the composite endpoint. Each AE will be counted once for a given participant and graded by severity and relationship to COVID-19 or study intervention. AEs will be presented by system organ class, duration (in days), start and stop-date. AEs leading to premature discontinuation from the study intervention and serious treatment-emergent AEs should be presented either in a table or a listing. Counts of the number of individuals experiencing SAEs and severe AEs will be tabulated by event type and treatment group. They will then be tabulated by organ system, duration (in days), start and stop-date. Then the total number of events of each type will be summarized by group, allowing for each individual to contribute a count response for the number of AEs.

Each AE will also be characterized in terms of its relationship with COVID-19 or the current study intervention. The occurrence of transfusion associated AEs will likewise be compared between the two groups based on proportions.

Co-enrollment in other clinical trials is allowed in this protocol so we will record the nature of the interventions in these other trials and, if possible, the particular intervention to which our study participants were assigned.

Cox regression models will also be fitted for the time to the first of each SAE and severe AE to deal with the fact that death and discharge are censoring events.

AEs leading to premature discontinuation from the study intervention and serious treatment-emergent AEs will be tabulated or presented as a listing.

12.4.4. **Sub-Group Analysis**

Subgroup analyses for the primary outcomes will evaluate the treatment effect across the following subgroups:

- Patients enrolled vs. not enrolled/treated in other therapeutic clinical trials
- >= 60 years versus less than 60 years of age
- Sex
- Ethnicity
    - White/Black/Hispanic/Asian/Other
- Presence of medical co-morbidities
    - diabetes (yes/no)
    - cardiac (yes/no)
    - respiratory (yes/no)
- Smoking History
    - Ever smoker/current smoker/never
    - 15 pack year history of smoking versus not
- Obesity (BMI >= 30 vs <30)
- Onset of symptoms > 12 days before randomization (including non-respiratory symptoms) versus not
- Timing of administration of CCP from diagnosis of COVID-19 (<=72 hours vs >72 hours)
- Severity of illness of donors (hospitalized vs not)
- ABO blood type of recipients (A vs other, B vs other, O vs other, AB vs other)
- CCP from one donor vs CCP from two donors
- Detectable vs non-detectable viral plasma viral load at baseline
- Anti-SARS-CoV-2 seropositivity at baseline
- Oxygen status at baseline (≤ 2L vs > 2L)

A forest plot will display confidence intervals across subgroups. Interaction tests will be conducted to determine whether the effect of treatment varies by subgroup.

12.4.5. **Exploratory Analysis**

Association between anti-SARS-CoV2 antibody in CCP and the primary outcome of intubation or death will be assessed using logistic regression. Anti-SARS-CoV-2 antibodies will be characterized according to the amount of antibodies against the native spike protein and the RBD, as well as to their neutralizing and Fc functions. Exploratory analyses will also examine possible associations between donor characteristics and treatment efficacy. Change in viral load and in anti-SARS-CoV2 serology in the recipient following transfusion will also be assessed and correlated with treatment outcome.

13. LIST OF APPENDICES

APPENDIX 1: Declaration of Helsinki

APPENDIX 2: Steering Committee Members

14. **REFERENCES**

1. Chan JFW, Kok KH, Zhu Z, et al. Genomic characterization of the 2019 novel human-pathogenic coronavirus isolated from a patient with atypical pneumonia after visiting Wuhan. *Emerg Microbes Infect*. 2020;9(1):221-236. doi:10.1080/22221751.2020.1719902
2. Wu JT, Leung K, Leung GM. Articles Nowcasting and forecasting the potential domestic and international spread of the 2019-nCoV outbreak originating in Wuhan , China : a modelling study. *Lancet*. 2020;395(10225):689-697. doi:10.1016/S0140-6736(20)30260-9
3. Zhang JS, Chen JT, Liu YX, et al. A serological survey on neutralizing antibody titer of SARS convalescent sera. *J Med Virol*. 2005;77(2):147-150. doi:10.1002/jmv.20431
4. Onder G, Rezza G, Brusaferro S. Case-Fatality Rate and Characteristics of Patients Dying in Relation to COVID-19 in Italy. *JAMA*. 2020. doi:10.1001/jama.2020.4683
5. Xu Z, Shi L, Wang Y, et al. Pathological findings of COVID-19 associated with acute respiratory distress syndrome. *Lancet Respir Med*. 2020;8(4):420-422. doi:10.1016/S2213-2600(20)30076-X
6. Liu L, Wei Q, Lin Q, et al. Anti-spike IgG causes severe acute lung injury by skewing macrophage responses during acute SARS-CoV infection. *JCI Insight*. 2019;4(4):e123158. doi:10.1172/jci.insight.123158
7. Casadevall A, Scharff MD. Return to the Past : The Case for Antibody-Based Therapies in Infectious Diseases. *Clin Infect Dis*. 1995;21(1):150-161.
8. Casadevall A, Dadachova E, Pirofski L. Passive antibody therapy for infectious diseases. *Nat Rev Microbiol*. 2004;2(9):695-703.
9. Sahr F, Ansumana R, Massaquoi TA, et al. Evaluation of convalescent whole blood for treating Ebola Virus Disease in Freetown, Sierra Leone. *J Infect*. 2017;74(3):302-309. doi:10.1016/j.jinf.2016.11.009
10. Van Griensven J, Edwards T, De Lamballerie X, et al. Evaluation of convalescent plasma for Ebola virus disease in Guinea. *N Engl J Med*. 2016;374(1):33-42. doi:10.1056/NEJMoa1511812
11. Chan KS, Lai ST, Chu CM, et al. Treatment of severe acute respiratory syndrome with lopinavir/ritonavir: A multicentre retrospective matched cohort study. *Hong Kong Med J*. 2003;9(6):399-406.
12. Casadevall A, Pirofski L. The convalescent sera option for containing COVID-19. *J Clin Invest*. 2020;130(4):1545-1548. doi:10.1172/jci138003
13. Borggren M, Jensen SS, Heyndrickx L, et al. Neutralizing Antibody Response and Antibody-Dependent Cellular Cytotoxicity in HIV-1-Infected Individuals from Guinea-Bissau and Denmark. *AIDS Res Hum Retroviruses*. 2016;32(5):434-442. doi:10.1089/AID.2015.0118
14. Mielke D, Bandawe G, Pollara J, et al. Antibody-Dependent Cellular Cytotoxicity (ADCC)-Mediating Antibodies Constrain Neutralizing Antibody Escape Pathway. *Front Immunol*. 2019;10:2875. doi:10.3389/fimmu.2019.02875
15. Cheng Y, Wong R, Soo YOY, et al. Use of convalescent plasma therapy in SARS patients in Hong Kong. *Eur J Clin Microbiol Infect Dis*. 2005;24(1):44-46. doi:10.1007/s10096-004-1271-9
16. Yeh K, Chiueh T, Siu LK, et al. Experience of using convalescent plasma for severe acute respiratory syndrome among healthcare workers in a Taiwan hospital.

2005;(September):919-922. doi:10.1093/jac/dki346
17. Ko JH, Seok H, Cho SY, et al. Challenges of convalescent plasma infusion therapy in Middle East respiratory coronavirus infection: A single centre experience. *Antivir Ther*. 2018;23(7):617-622. doi:10.3851/IMP3243
18. Arabi YM, Hajeer AH, Luke T, et al. Feasibility of using convalescent plasma immunotherapy for MERS-CoV infection, Saudi Arabia. *Emerg Infect Dis*. 2016;22(9):1554-1561. doi:10.3201/eid2209.151164
19. Duan K, Liu B, Li C, et al. The feasibility of convalescent plasma therapy in severe COVID-19 patients: a pilot study. *medRxiv*. 2020;(8):2020.03.16.20036145. doi:10.1101/2020.03.16.20036145
20. Shen C, Wang Z, Zhao F, et al. Treatment of 5 Critically Ill Patients With COVID-19 With Convalescent Plasma. *Jama*. 2020;(29):1-8. doi:10.1001/jama.2020.4783
21. Zhang B, Liu S, Tan T, et al. Treatment with convalescent plasma for critically ill patients with SARS-CoV-2 infection. *Chest*. 2020. doi:10.1016/j.chest.2020.03.039
22. US FDA. Investigational COVID-19 Convalescent Plasma - Emergency INDs. https://www.fda.gov/vaccines-blood-biologics/investigational-new-drug-ind-or-device-exemption-ide-process-cber/investigational-covid-19-convalescent-plasma-emergency-inds. Published 2020. Accessed March 26, 2020.
23. Mair-Jenkins J, Saavedra-Campos M, Baillie JK, et al. The effectiveness of convalescent plasma and hyperimmune immunoglobulin for the treatment of severe acute respiratory infections of viral etiology: A systematic review and exploratory meta-analysis. *J Infect Dis*. 2015;211(1):80-90. doi:10.1093/infdis/jiu396
24. Xinhua. China puts 245 COVID-19 patients on convalescent plasma therapy. http://www.xinhuanet.com/english/2020-02/28/c_138828177.htm. Published 2020. Accessed March 26, 2020.
25. Crowe JE, Firestone C-Y, Murphy BR. Passively Acquired Antibodies Suppress Humoral But Not Cell-Mediated Immunity in Mice Immunized with Live Attenuated Respiratory Syncytial Virus Vaccines. *J Immunol*. 2001;167(7):3910-3918. doi:10.4049/jimmunol.167.7.3910
26. Bertoye A. L ’ Immunisation Passive. *Bull Wld Hlth Org*. 1955:423-435.
27. Casadevall A, Scharff MD. Serum therapy revisited: Animal models of infection and development of passive antibody therapy. *Antimicrob Agents Chemother*. 1994;38(8):1695-1702. doi:10.1128/AAC.38.8.1695
28. Stoll H. Value of convalescent blood and serum in treatment of influenza pneumonia. *JAMA*. 1919;73:478-483.
29. Ross C, Hund E. Treatment of pneumonic disturbance complicating influenza. *JAMA*. 1919;72:640-645.
30. Hung IFN, To KKW, Lee CK, et al. Hyperimmune IV immunoglobulin treatment: A multicenter double-blind randomized controlled trial for patients with severe 2009 influenza A(H1N1) infection. *Chest*. 2013;144(2):464-473. doi:10.1378/chest.12-2907
31. Bouadma L, Lescure FX, Lucet JC, Yazdanpanah Y, Timsit JF. Severe SARS-CoV-2 infections: practical considerations and management strategy for intensivists. *Intensive Care Med*. 2020;46(4):579-582. doi:10.1007/s00134-020-05967-x
32. Treatment P a FHN, Luke TC, Kilbane EM, Jackson JL, Hoffman SL. Annals of Internal Medicine Review Meta-Analysis : Convalescent Blood Products for Spanish Influenza. *Ann*

Intern Med. 2006;145(8).
33. Soo Y, Cheng Y, Wong R, et al. Retrospective comparison of convalescent plasma with continuing high-dose methylprenidsolone treatment in SARS patients. *J Chem Inf Model*. 2013;53(9):1689-1699. doi:10.1017/CBO9781107415324.004
34. Wong V, Dai D, Wu A. Treatment of severe acute respiratory syndrome with convalescent plasma. *Hong Kong Med J*. 2003;9(3):199-201.
35. Zhou X, Zhao M, Wang F. Epidemiological features, clinical diagnosis and therapy of first cluster of patients with severe acute respiratory syndrome in Beijing area. *Zhonghua yi zue za zhi*. 2003;82(12):1018-1022.
36. Kong LK, Zhou BP. Successful treatment of avian influenza with convalescent plasma [1]. *Hong Kong Med J*. 2006;12(6):489.
37. Yu H, Gao Z, Feng Z, et al. Clinical characteristics of 26 human cases of highly pathogenic avian influenza A (H5N1) virus infection in China. *PLoS One*. 2008;3(8). doi:10.1371/journal.pone.0002985
38. Zhou B, Zhong N, Guan Y. Treatment with convalescent plasma for influenza A (H5N1) infection [9]. *N Engl J Med*. 2007;357(14):1450-1451. doi:10.1056/NEJMc070359
39. Hung IFN, To KKW, Lee CK, et al. Convalescent plasma treatment reduced mortality in patients with severe pandemic influenza A (H1N1) 2009 virus infection. *Clin Infect Dis*. 2011;52(4):447-456. doi:10.1093/cid/ciq106
40. Chan KKC, Lee KL, Lam PKN, Law KI, Joynt GM, Yan WW. Hong Kong's experience on the use of extracorporeal membrane oxygenation for the treatment of influenza A (H1N1). *Hong Kong Med J*. 2010;16(6):447-454.
41. Kraft CS, Hewlett AL, Koepsell S, et al. The Use of TKM-100802 and Convalescent Plasma in 2 Patients with Ebola Virus Disease in the United States. *Clin Infect Dis*. 2015;61(4):496-502. doi:10.1093/cid/civ334
42. Young MK, Nimmo GR, Cripps AW, Jones MA. Post-exposure passive immunisation for preventing measles. *Cochrane Database Syst Rev*. 2014;2014(4). doi:10.1002/14651858.CD010056.pub2
43. Rambar A. Mumps: Use of Convalescent Serum in the Treatment and Prophylaxis of Orchitis. *Am J Dis Child*. 1946;71(1):1-13.
44. O'Brien P, Fleming T. A Multiple Testing Procedure for Clinical Trials. *Biometrics*. 1979;35:549-556.
45. Beigel JH, Tebas P, Elie-Turenne MC, et al. Immune plasma for the treatment of severe influenza: an open-label, multicentre, phase 2 randomised study. *Lancet Respir Med*. 2017;5(6):500-511. doi:10.1016/S2213-2600(17)30174-1
46. Beigel JH, Aga E, Elie-Turenne MC, et al. Anti-influenza immune plasma for the treatment of patients with severe influenza A: a randomised, double-blind, phase 3 trial. *Lancet Respir Med*. 2019;7(11):941-950. doi:10.1016/S2213-2600(19)30199-7
47. Davey RT, Fernández-Cruz E, Markowitz N, et al. Anti-influenza hyperimmune intravenous immunoglobulin for adults with influenza A or B infection (FLU-IVIG): a double-blind, randomised, placebo-controlled trial. *Lancet Respir Med*. 2019;7(11):951-963. doi:10.1016/S2213-2600(19)30253-X
48. To KK, Tak O, Tsang Y, et al. Articles Temporal profiles of viral load in posterior oropharyngeal saliva samples and serum antibody responses during infection by SARS-CoV-

2 : an observational cohort study. *Lancet Infect Dis*. 2020;3099(20):1-10. doi:10.1016/S1473-3099(20)30196-1
49. Herdman M, Gudex C, Lloyd A, et al. Development and preliminary testing of the new five-level version of EQ-5D (EQ-5D-5L). *Qual Life Res*. 2011;20(10):1727-1736. doi:10.1007/s11136-011-9903-x
50. Cook D, Lauzier F, Rocha MG, Sayles MJ, Finfer S. Serious adverse events in academic critical care research. *CMAJ*. 2008;178(9):1181-1184. doi:10.1503/cmaj.071366
51. Ahmed SM, Palermo AGS. Community engagement in research: Frameworks for education and peer review. *Am J Public Health*. 2010;100(8):1380-1387. doi:10.2105/AJPH.2009.178137
52. World Health Organisation. *RCCE Action Plan Guidance Covid-19 Preparedness & Response*.; 2020. https://www.who.int/publications-detail/risk-communication-and-community-engagement-(rcce)-action-plan-guidance.
53. Holzer JK, Ellis L, Merritt MW. Why we need community engagement in medical research. *J Investig Med*. 2014;62(6):851-855. doi:10.1097/JIM.0000000000000097
54. Lan K, DeMets D. Discrete sequential boundaries for clinical trials. *Biometrika*. 1983;70:659-663.

APPENDIX 1 : Declaration of Helsinki

WMA DECLARATION OF HELSINKI
ETHICAL PRINCIPLES FOR MEDICAL RESEARCH INVOLVING HUMAN SUBJECTS

Adopted by the 18th WMA General Assembly, Helsinki, Finland, June 1964
and amended by the:
29th WMA General Assembly, Tokyo, Japan, October 1975
35th WMA General Assembly, Venice, Italy, October 1983
41st WMA General Assembly, Hong Kong, September 1989
48th WMA General Assembly, Somerset West, Republic of South Africa, October 1996
52nd WMA General Assembly, Edinburgh, Scotland, October 2000
53rd WMA General Assembly, Washington DC, USA, October 2002 (Note of Clarification added)
55th WMA General Assembly, Tokyo, Japan, October 2004 (Note of Clarification added)
59th WMA General Assembly, Seoul, Republic of Korea, October 2008
64th WMA General Assembly, Fortaleza, Brazil, October 2013

Preamble

1. The World Medical Association (WMA) has developed the Declaration of Helsinki as a statement of ethical principles for medical research involving human subjects, including research on identifiable human material and data.

The Declaration is intended to be read as a whole and each of its constituent paragraphs should be applied with consideration of all other relevant paragraphs.

2. Consistent with the mandate of the WMA, the Declaration is addressed primarily to physicians. The WMA encourages others who are involved in medical research involving human subjects to adopt these principles.

General Principles

3. The Declaration of Geneva of the WMA binds the physician with the words, “The health of my patient will be my first consideration,” and the International Code of Medical Ethics declares that, “A physician shall act in the patient’s best interest when providing medical care.”
4. It is the duty of the physician to promote and safeguard the health, well-being and rights of patients, including those who are involved in medical research. The physician’s knowledge and conscience are dedicated to the fulfilment of this duty.
5. Medical progress is based on research that ultimately must include studies involving human subjects.
6. The primary purpose of medical research involving human subjects is to understand the causes, development and effects of diseases and improve preventive, diagnostic and therapeutic interventions (methods, procedures and treatments). Even the best proven interventions must be evaluated continually through research for their safety, effectiveness, efficiency, accessibility and quality.
7. Medical research is subject to ethical standards that promote and ensure respect for all human subjects and protect their health and rights.
8. While the primary purpose of medical research is to generate new knowledge, this goal can never take precedence over the rights and interests of individual research subjects.

9. It is the duty of physicians who are involved in medical research to protect the life, health, dignity, integrity, right to self-determination, privacy, and confidentiality of personal information of research subjects. The responsibility for the protection of research subjects must always rest with the physician or other health care professionals and never with the research subjects, even though they have given consent.
10. Physicians must consider the ethical, legal and regulatory norms and standards for research involving human subjects in their own countries as well as applicable international norms and standards. No national or international ethical, legal or regulatory requirement should reduce or eliminate any of the protections for research subjects set forth in this Declaration.
11. Medical research should be conducted in a manner that minimizes possible harm to the environment.
12. Medical research involving human subjects must be conducted only by individuals with the appropriate ethics and scientific education, training and qualifications. Research on patients or healthy volunteers requires the supervision of a competent and appropriately qualified physician or other health care professional.
13. Groups that are underrepresented in medical research should be provided appropriate access to participation in research.
14. Physicians who combine medical research with medical care should involve their patients in research only to the extent that this is justified by its potential preventive, diagnostic or therapeutic value and if the physician has good reason to believe that participation in the research study will not adversely affect the health of the patients who serve as research subjects.
15. Appropriate compensation and treatment for subjects who are harmed as a result of participating in research must be ensured.

Risks, Burdens and Benefits

16. In medical practice and in medical research, most interventions involve risks and burdens.

Medical research involving human subjects may only be conducted if the importance of the objective outweighs the risks and burdens to the research subjects.

17. All medical research involving human subjects must be preceded by careful assessment of predictable risks and burdens to the individuals and groups involved in the research in comparison with foreseeable benefits to them and to other individuals or groups affected by the condition under investigation.

Measures to minimize the risks must be implemented. The risks must be continuously monitored, assessed and documented by the researcher.

18. Physicians may not be involved in a research study involving human subjects unless they are confident that the risks have been adequately assessed and can be satisfactorily managed.

When the risks are found to outweigh the potential benefits or when there is conclusive proof of definitive outcomes, physicians must assess whether to continue, modify or immediately stop the study.

Vulnerable Groups and Individuals

19. Some groups and individuals are particularly vulnerable and may have an increased likelihood of being wronged or of incurring additional harm.

All vulnerable groups and individuals should receive specifically considered protection.

20. Medical research with a vulnerable group is only justified if the research is responsive to the health needs or priorities of this group and the research cannot be carried out in a non-vulnerable group. In addition, this group should stand to benefit from the knowledge, practices or interventions that result from the research.

Scientific Requirements and Research Protocols

21. Medical research involving human subjects must conform to generally accepted scientific principles, be based on a thorough knowledge of the scientific literature, other relevant sources of information, and adequate laboratory and, as appropriate, animal experimentation. The welfare of animals used for research must be respected.

22. The design and performance of each research study involving human subjects must be clearly described and justified in a research protocol.

The protocol should contain a statement of the ethical considerations involved and should indicate how the principles in this Declaration have been addressed. The protocol should include information regarding funding, sponsors, institutional affiliations, potential conflicts of interest, incentives for subjects and information regarding provisions for treating and/or compensating subjects who are harmed as a consequence of participation in the research study.

In clinical trials, the protocol must also describe appropriate arrangements for post-trial provisions.

Research Ethics Committees

23. The research protocol must be submitted for consideration, comment, guidance and approval to the concerned research ethics committee before the study begins. This committee must be transparent in its functioning, must be independent of the researcher, the sponsor and any other undue influence and must be duly qualified. It must take into consideration the laws and regulations of the country or countries in which the research is to be performed as well as applicable international norms and standards, but these must not be allowed to reduce or eliminate any of the protections for research subjects set forth in this Declaration.

The committee must have the right to monitor ongoing studies. The researcher must provide monitoring information to the committee, especially information about any serious AEs. No amendment to the protocol may be made without consideration and approval by the committee. After the end of the study, the researchers must submit a final report to the committee containing a summary of the study's findings and conclusions.

Privacy and Confidentiality

24. Every precaution must be taken to protect the privacy of research subjects and the confidentiality of their personal information.

Informed Consent

25. Participation by individuals capable of giving informed consent as subjects in medical research must be voluntary. Although it may be appropriate to consult family members or community leaders, no individual capable of giving informed consent may be enrolled in a research study unless he or she freely agrees.

26. In medical research involving human subjects capable of giving informed consent, each potential subject must be adequately informed of the aims, methods, sources of funding, any possible conflicts of interest, institutional affiliations of the researcher, the anticipated benefits and potential risks of the study and the discomfort it may entail, post-study provisions and any other relevant aspects of the study. The potential subject must be informed of the right to refuse to participate in the study or to withdraw consent to participate at any time without reprisal. Special attention should be given to the specific information needs of individual potential subjects as well as to the methods used to deliver the information.

After ensuring that the potential subject has understood the information, the physician or another appropriately qualified individual must then seek the potential subject's freely-given informed consent, preferably in writing. If the consent cannot be expressed in writing, the non-written consent must be formally documented and witnessed.

All medical research subjects should be given the option of being informed about the general outcome and results of the study.

27. When seeking informed consent for participation in a research study the physician must be particularly cautious if the potential subject is in a dependent relationship with the physician or may consent under duress. In such situations the informed consent must be sought by an appropriately qualified individual who is completely independent of this relationship.
28. For a potential research subject who is incapable of giving informed consent, the physician must seek informed consent from the legally authorized representative. These individuals must not be included in a research study that has no likelihood of benefit for them unless it is intended to promote the health of the group represented by the potential subject, the research cannot instead be performed with persons capable of providing informed consent, and the research entails only minimal risk and minimal burden.
29. When a potential research subject who is deemed incapable of giving informed consent is able to give assent to decisions about participation in research, the physician must seek that assent in addition to the consent of the legally authorized representative. The potential subject's dissent should be respected.
30. Research involving subjects who are physically or mentally incapable of giving consent, for example, unconscious patients, may be done only if the physical or mental condition that prevents giving informed consent is a necessary characteristic of the research group. In such circumstances the physician must seek informed consent from the legally authorized representative. If no such representative is available and if the research cannot be delayed, the study may proceed without informed consent provided that the specific reasons for involving subjects with a condition that renders them unable to give informed consent have been stated in the research protocol and the study has been approved by a research ethics committee. Consent to remain in the research must be obtained as soon as possible from the subject or a legally authorized representative.
31. The physician must fully inform the patient which aspects of their care are related to the research. The refusal of a patient to participate in a study or the patient's decision to withdraw from the study must never adversely affect the patient-physician relationship.
32. For medical research using identifiable human material or data, such as research on material or data contained in biobanks or similar repositories, physicians must seek informed consent for its collection, storage and/or reuse. There may be exceptional situations where consent would be impossible or

impracticable to obtain for such research. In such situations the research may be done only after consideration and approval of a research ethics committee.

Use of Placebo

33. The benefits, risks, burdens and effectiveness of a new intervention must be tested against those of the best proven intervention(s), except in the following circumstances:

Where no proven intervention exists, the use of placebo, or no intervention, is acceptable; or

Where for compelling and scientifically sound methodological reasons the use of any intervention less effective than the best proven one, the use of placebo, or no intervention is necessary to determine the efficacy or safety of an intervention and the patients who receive any intervention less effective than the best proven one, placebo, or no intervention will not be subject to additional risks of serious or irreversible harm as a result of not receiving the best proven intervention.

Extreme care must be taken to avoid abuse of this option.

Post-Trial Provisions

34. In advance of a clinical trial, sponsors, researchers and host country governments should make provisions for post-trial access for all participants who still need an intervention identified as beneficial in the trial. This information must also be disclosed to participants during the informed consent process.

Research Registration and Publication and Dissemination of Results

35. Every research study involving human subjects must be registered in a publicly accessible database before recruitment of the first subject.

36. Researchers, authors, sponsors, editors and publishers all have ethical obligations with regard to the publication and dissemination of the results of research. Researchers have a duty to make publicly available the results of their research on human subjects and are accountable for the completeness and accuracy of their reports. All parties should adhere to accepted guidelines for ethical reporting. Negative and inconclusive as well as positive results must be published or otherwise made publicly available. Sources of funding, institutional affiliations and conflicts of interest must be declared in the publication. Reports of research not in accordance with the principles of this Declaration should not be accepted for publication.

Unproven Interventions in Clinical Practice

In the treatment of an individual patient, where proven interventions do not exist, or other known interventions have been ineffective, the physician, after seeking expert advice, with informed consent from the patient or a legally authorized representative, may use an unproven intervention if in the physician's judgement it offers hope of saving life, re-establishing health or alleviating suffering. This intervention should subsequently be made the object of research, designed to evaluate its safety and efficacy. In all cases, new information must be recorded and, where appropriate, made publicly available.

APPENDIX 2: Steering Committee

Co-Principal Investigators

Donald Arnold<br>arnold@mcmaster.ca	Director of the McMaster Centre for Transfusion Research<br>Associate Professor, Division of Hematology and Thromboembolism, Department of Medicine, McMaster University
Philippe Begin<br>philippe.begin@umontreal.ca	Associate Professor, Department of Medicine, Université de Montréal<br>Director, Oral Immunotherapy Clinic (CITO), CHU Sainte-Justine<br>Immuno-Allergist, CHU Sainte-Justine<br>Researcher, CHU Sainte-Justine

Steering Committee

Alan Tinmouth<br>atinmouth@toh.ca	Transfusion Medicine	Director, OHRI Centre for Transfusion Research, Clinical Epidemiology Program, Ottawa Hospital Research Institute; Associate Professor, Hematology, University of Ottawa
Jeannie Callum<br>jeannie.callum@sunnybrook.ca	Transfusion Medicine	Lead for the QUEST University of Toronto Transfusion Research Team; Professor, Laboratory Medicine and Pathobiology
Michelle Zeller<br>zeller@mcmaster.ca	Transfusion Medicine	Assistant Professor, Division of Hematology and Thromboembolism, Department of Medicine, McMaster University
Nancy Heddle<br>heddlen@mcmaster.ca	Methodology	Professor Emeritus, Department of Medicine, McMaster University
Richard Cook<br>rjcook@uwaterloo.ca	Statistician	Professor, Faculty of Statistics and Actuarial Science, University of Waterloo
Damon Scales<br>damon.scales@sunnybrook.ca	Intensivist Methodologist	Chief, Department of Critical Care Medicine, Sunnybrook Health Sciences Centre; Professor, Department of Medicine, interdepartmental division of critical care, University of Toronto
Alison McGeer<br>Allison.McGeer@sinaihealth.ca	Infectious Diseases	Professor, Departments of Laboratory Medicine and Pathobiology and Public Health Sciences, University of Toronto; Director of Infection Control, Mount Sinai Hospital
Dana Devine<br>dana.devine@blood .ca	Transfusion Scientist	Chief Medical & Scientific Officer, Canadian Blood Services; Professor, Pathology and Laboratory Medicine, Medicine
Lisa Schwartz<br>schwar@mcmaster.ca	Statistician Ethicist	Associate Professor, Department of Clinical Epidemiology & Biostatistics, Faculty of Health Sciences, McMaster University
Dean Ferguson<br>dafergusson@ohri.ca	Methodologist	Senior Scientist, Clinical Epidemiology Program, Ottawa Hospital Research Institute; Director, Clinical Epidemiology Program, Ottawa Hospital Research Institute; Professor, Departments of Medicine, Surgery, & the School of Epidemiology and Public Health, University of Ottawa
Richard Carl<br>richardcarl@sympatico.ca	Patient	Patient representative; Director of several corporations (Oil and gas and financial services; risk management)
Ryan Zarychanski<br>rzarychanski@cancercare.mb.ca	Critical Care Medicine and	Assistant Professor and Clinician-Scientist at the University of Manitoba in the Department of

	Hematology	Medicine, Sections of Critical Care and of Hematology/Medical Oncology
Renee Bazin<br>Renee.bazin@hema-quebec.qc.ca	Scientist, Hema Quebec	Associate Professor in the Department of Biochemistry and Microbiology and in the Faculty of Pharmacy at Laval University. Director of Medical Affairs and Innovcation at Hema-Quebec
Nadine Shehata<br>Nadine.Shehata@sinaihealth.ca	Hematology	Head, Transfusion Medicine, Mount Sinai Hospital, Toronto. Associate Professor,Departments of Medicine,<br>Laboratory Medicine and Pathobiology and Institute of Health Policy Management &<br>Evaluation, University of Toronto. Associate Scientist, Keenan Research Centre, Li Ka Shing Knowledge Institute, St Michaels Hospital.
Alexis Turgeon<br>Alexis.turgeon@crchudequebec.ulaval.ca	Critical Care and Trauma	Associate Professor and Director of research, Division of Critical Care Medicine and Department of Anesthesiology and Critical Care, Laval University.
Nancy Robitaille<br>Nancy.Robitaille.MD@hema-quebec.qc.ca	Pediatric Hematologist	Associate Professor, Department of Pediatrics, Université de Montréal<br>Pediatric hematologist, CHU Sainte-Justine<br>Vice-president, Transfusion Medicine, Héma-Québec
Michael Chassé<br>michael.chasse@umontreal.ca	Critical Care	Intensivist, Centre Hospitalier de l'Unversite de Montreal (CHUM). Assistant Professor, Department of Medicine, University of Montreal
Nick Daneman<br>Nick.daneman@sunnybrook.ca	Infectious Disease	Division of Infectious Diseases, Department of Medicine, University of Toronto; Infectious Diseases, Department of Medicine
Melissa Cushing<br>mec2013@med.cornell.edu	Transfusion Medicine	Transfusion Medicine and Cellular Therapy, New York-Presbyterian; Pathology and Laboratory Medicine, Weill Cornell Medicine, Cornell University
Bruce Sachais<br>BSachais@nybc.org	Transfusion and Apheresis Medicine	Chief Medical Officer, Vice President - NYBC Enterprises<br>Professor, Pathology and Laboratory Medicine - Weill Cornell Medical College
Luiz Amorim<br>luizamorimfilho@gmail.com	Transfusion Medicine Hematology	Director, Hemorio, Rio de Janeiro, Brazil
Kent Cadogan Loftsgard<br>Kent-l@usa.net	Patient (Congenital Spastic Cerebral Palsy) Community Advisory Committee Representative	Clinical Educator/Health Researcher - University of British Columbia/Vancouver Coastal Health/Canadian Institutes of Health Research, and others.<br><br>Extensive experience includes health specialty broadcast & print communications production.

Statistical Analysis Plan for

A Randomized Open-Label Trial of CONvalescent Plasma for Hospitalized Adults With Acute COVID-19 Respiratory Illness (CONCOR-1)

**Co-Lead Principal Investigators:**	Dr Donald Arnold<br>Dr Philippe Bégin<br>Dr Jeannie Callum
**Investigational product:**	COVID-19 Convalescent Plasma
**Protocol #:**	GRAAL-2020-01
**SAP for Protocol Version and Date:**	Version 8.0 15 Jan 2021

The study will be conducted according to the protocol and in compliance with Good Clinical Practice (GCP), with the Declaration of Helsinki and with other applicable regulatory requirements.

Contents

1. Study Design and Objectives 1
1.1. Summary of Design 1
1.2. Primary Study Objectives 1
1.3. Sample Size Justification 1
2. Responses 1
2.1. Primary Outcome 1
2.2. Secondary Outcomes 1
2.2.1. Time to intubation or death 1
2.2.2. Ventilator-free days 1
2.2.3. In-hospital death by Day 90 2
2.2.4. Time to in-hospital death 2
2.2.5. Death by Day 30 2
2.2.6. Length of stay in intensive care unit 2
2.2.7. Length of stay in hospital 2
2.2.8. Need for Extra-Corporeal Membrane Oxygenation (ECMO) 2
2.2.9. Need for renal replacement therapy 2
2.2.10. Myocarditis 2
2.2.11. Patient-reported outcomes (PROs) 3
2.2.12. Incremental cost per quality-adjusted life year (QALY) 3
2.3. Adverse Events 3
2.3.1. Adverse event reporting 3
2.3.2. Adverse events to Convalescent Plasma Transfusion 3
3. Analysis Sets 3
3.1. Intention to Treat Sample 3
3.2. Per Protocol Sample 4
3.3. Safety Sample 4
4. Statistical Analysis 4
4.1. Handling of Incomplete Data 4
4.2. Analysis of Primary Outcome 5
4.3. Analyses of Secondary Outcomes 5
4.3.1. Time to intubation or death 5

4.3.2. Ventilator-free days 5
4.3.3. In-hospital death by Day 90 5
4.3.4. Time to in-hospital death 6
4.3.5. Death by Day 30 6
4.3.6. Length of stay in intensive care unit 6
4.3.7. Length of stay in hospital 6
4.3.8. Need for Extra-Corporeal Membrane Oxygenation (ECMO) 7
4.3.9. Need for renal replacement therapy 7
4.3.10. Myocarditis 7
4.3.11. Patient-reported outcomes (PROs) 7
4.3.12. Incremental cost per quality-adjusted life year (QALY) 7
4.3.13. CPP transfusion-associated adverse events at Day 30 7
4.3.12. Grade 3, 4, and 5 adverse events at Day 30 8
4.3.13. Grade 3, 4 and 5 serious adverse events at Day 30 8
4.4. Sub-Group Analyses 8
4.5. Safety Reports for the IDSMC 9
4.6. Exploratory Analysis of the Role of anti-SARS-CoV-2 antibodies 9
4.6.1. Neutralizing antibodies 9
4.6.2. Viral load 10
4.7. Schedule for Primary Interim Analysis 10
4.8. The Lan-DeMets Error Spending Function 11
4.9. Interim Calculation of Conditional Power for Futility 11
4.10. Sample Size Re-Estimation 12
4.11. Conventions Regarding Blinding 12
5. References 13

1. Study Design and Objectives

1.1.Summary of Design

This is a multi-centre open-label randomized controlled trial in which recruited patients will be randomized in a 2:1 ratio to receive either 500 mL of COVID-19 convalescent plasma (CCP) comprised of a 500 mL unit from one donor or two 250 mL units from one or two donors collected by apheresis, or standard of care. The plan is to randomize 1200 individuals to obtain an expected 800 patients in the CCP arm and 400 patients in the arm receiving standard of care.

1.2.Primary Study Objectives

The primary objective is to determine the effect of COVID-19 convalescent plasma (CCP) on the risk of intubation or death at Day 30 in adult patients hospitalized for COVID-19 respiratory illness.

1.3.Sample Size Justification

Assuming a 30% risk of the composite outcome of intubation or death by Day 30 among hospitalized patients under standard of care, a sample size of 1200 individuals (800 in the CCP arm, and 400 in the standard of care arm) will provide 80% power ($\beta = 0.20$) to detect a relative risk reduction of 25% with CCP therapy using a 2-tailed test at level $\alpha = 0.05$. We assume that no patient will be lost to follow-up.

2. Responses

2.1.Primary Outcome

The primary outcome is intubation or death at Day 30 (the date of randomization being Day 1).

2.2.Secondary Outcomes

2.2.1.Time to intubation or death

The number of days from randomization to the first event of intubation or in-hospital death will be recorded. The censoring time for individuals not receiving intubation or dying will be the minimum of i. the number of days from randomization to discharge, and ii. Day 30. The 30-day limit is specified to align with the time horizon of the primary outcome.

2.2.2.Ventilator-free days

The number of ventilator-free days between randomization and Day 30 will be computed as the number of days during this 30-day period of time during which they were alive and not requiring ventilation. This total number of days could be made up days prior to ventilation and the number of days after discontinuation of ventilation if ventilation is required. Patients will be contacted at Day 30 by telephone to determine whether there were any ICU admissions since discharge from the hospital they were in at the time of recruitment. Patients discharged from hospital will be assumed to be off-ventilator post-discharge unless otherwise indicated.

2.2.3.In-hospital death by Day 90

This response will be a binary indicator of whether a patient died in-hospital within 90 days of randomization.

2.2.4.Time to in-hospital death

The number of days from randomization to in-hospital death will be recorded. The censoring time for individuals not dying in hospital will be the minimum of i. the number of days from randomization to discharge, and ii. to Day 90. The 90-day limit is specified to be large enough that it is highly unlikely to be the factor leading to censoring, but is small enough that it will mitigate the need to follow individuals who are hospitalized for an unusually long time until they are discharged.

2.2.5.Death by Day 30

This response is a simple binary indicator indicating whether the patient has died between randomization and Day 30.

2.2.6.Length of stay in intensive care unit

The number of days spent in the intensive care unit (ICU) over the 30-day period following randomization will be recorded. This total number of ICU days could be made up days from multiple admissions within the 30-day period from randomization if they are discharged and re-admitted a second time within this 30-day period. Patients will be contacted at Day 30 by telephone to determine whether there were any hospital admissions since discharge from the hospital they were in at the time of recruitment. Patients discharged from hospital will be assumed not to be in an ICU elsewhere, unless otherwise indicated.

2.2.7.Length of stay in hospital

The number of days from randomization to the first event of death or discharge will be recorded as the length of stay in hospital. This time will be right-censored at 90 days to avoid the need to follow individuals who are hospitalized for a particularly long time until discharge; it is expected that most hospitalizations will not last beyond 90 days.

2.2.8.Need for Extra-Corporeal Membrane Oxygenation (ECMO)

This response will be a binary indicator indicating whether the patient required extra-corporeal membrane oxygenation during the 30-day period following randomization.

2.2.9.Need for renal replacement therapy

This response will be the binary variable indicating that the patient required renal replacement therapy during the 30-day period following randomization.

2.2.10. Myocarditis

This binary response will indicate whether the patient developed myocarditis (as defined by the medical team) between randomization and Day 30.

2.2.11.Patient-reported outcomes (PROs)

PRO will be measured using a widely used generic instrument called EQ-5D-5L. It measures health-related quality of life in five dimensions, namely, mobility, self-care, usual activities, pain/discomfort, and anxiety/depression. Patients can report five level impairment, reflecting no, slight, moderate, severe, and extreme problems in each dimension. The EQ-5D-5L will be administered at baseline and Day 30.

2.2.12.Incremental cost per quality-adjusted life year (QALY)

A cost effectiveness analysis alongside the trial will be conducted. The summary measure for the economic evaluation is incremental cost per QALY comparing CCP with standard care over the 30-day trial period.

2.3.Adverse Events

2.3.1.Adverse event reporting

The severity of each AE will be assessed by the Investigator and graded based on the Common Terminology Criteria for Adverse Events (CTCAE) version 4.0. Adverse events graded as 1 or 2 require no further documentation, unless they are related or probably related to CCP. Adverse events graded as 3, 4 and 5 must be reported on the eCRF within 7 day and are categorized as expected or unexpected. Serious adverse events (SAEs) deemed to be unexpected and related to or probably related to the investigational products must be reported to the sponsor within 24 hours. All other SAEs must be reported to the sponsor within 96 hours (Protocol section 9).

2.3.2.Adverse events to Convalescent Plasma Transfusion

Adverse events to convalescent plasma will be captured and graded using two approaches:

1. CCP-related AEs will be graded using the CTCAE criteria as specified in 2.3.1
2. CCP-related AEs will also be categorized using the ISBT criteria for acute transfusion reactions including name of the reaction, and severity (Grade 1-4)

3. Analysis Sets

Given the simple nature of the outcomes it is anticipated that outcomes will be possible to collect for most individuals.

3.1. Intention to Treat Sample

The intention to treat (ITT) sample will be comprised of all patients who were randomized irrespective of whether they received the randomized intervention. That is, individuals randomized to the CCP arm who did not receive CCP would have their outcomes attributed to the CCP arm. Given the simple nature of the primary outcome we anticipate a very low rate of missing primary outcome data.

The primary analysis will be based on individuals with available outcome data and this is expected to be all patients given the simple nature of the primary outcome. For some secondary outcomes if an outcome is unknown for some individuals, analyses will be first

carried out for those with complete data (i.e. in a complete case analysis) based on a modified intention to treat sample (i.e. the intention to treat sample excluding those individuals with unknown outcome). Multiple imputation will be carried out for a sensitivity analysis in which outcome data will be generated for those where it is missing in order to approximate a true intention to treat analysis. For details of the multiple imputation procedure see Section 4.1.

3.2. Per Protocol Sample

The per protocol sample will be comprised of eligible individuals who are randomized and are treated according to the protocol. In this trial, patients will be excluded from the per protocol analysis if any of the following protocol violations occur:
- Received less than two units of CCP*
- Transfusion of second unit of CCP completed > 24 hours after randomization*
- Patient randomized to SOC but receives CCP
- Patient was randomized despite being ineligible for the trial

*special consideration will be given for instances where 1 x 500 mL unit was given

3.3. Safety Sample

The safety sample will be comprised of all randomized individuals and so will coincide with the intention to treat sample.

4. Statistical Analysis

In what follows, the analysis plans are described for the sample representing the intention to treat population. Similar analyses will be carried out for the modified intention to treat and per protocol samples.

4.1. Handling of Incomplete Data

Efforts to minimize loss-to-follow up will be considerable and we are expected to have complete data on individuals for the primary and secondary outcomes. Given the simple nature of the primary outcome it is not expected that this response will be missing. If an individual withdraws from the study early, their time to event outcomes will be censored at the time of withdrawal. For binary outcomes where data are missing, a first analysis will be based on a complete case analysis using individuals who provide information on the outcomes; this is not strictly an intention to treat analysis but rather is based on a modified intention to treat sample. There is a chance that the complete case analysis will yield biased estimates of treatment effect (Kenward and Carpenter, 2007); therefore, while there is controversy about the appropriateness of imputing outcome data in clinical trials, multiple imputation (Little and Rubin, 2002) will be used as a sensitivity analysis to enable a genuine intention to treat sample. This will involve imputing missing data via imputation models fitted to the data from individuals providing complete information. Covariates to be included in the imputation model include age, sex, key co-morbidities (e.g. diabetes, cardiovascular disease, kidney disease), treatment and the time of withdrawal from the study**.** Standard multiple imputation will be adopted using m =

50 imputed datasets with the results combined across samples and variance estimates computed based on Rubin's rules (Rubin, 1996).

4.2.Analysis of Primary Outcome

If we let p_e and p_s denote the probability of the composite outcome (intubation or death at Day 30) for the experimental (CCP) and standard of care arms respectively, then the null and alternative hypotheses are H_0: $p_e = p_s$ and H_1: $p_e \neq p_s$, respectively. The probability of intubation or death between randomization and Day 30 will be estimated for each treatment group by the proportion of individuals randomized to each group who required ventilation or died by Day 30. The primary analysis will be based on a 2-sided Wald test of the null hypothesis that probability of intubation or death is the same among individuals receiving CCP or standard of care. The percent relative risk reduction, defined by $100(1 - p_e/p_s)$, will be estimated along with a 95% confidence interval.

4.3.Analyses of Secondary Outcomes

4.3.1.Time to intubation or death

The time to intubation for mechanical ventilation will be analyzed using a cause-specific Cox regression model with censoring occurring at the time of discharge (Kalbfleisch and Prentice, 2011). That is, individuals who are intubation-free and alive at the time of discharge will have their follow-up censored at the time of hospital discharge. The regression coefficient and associated standard error from the cause-specific Cox regression model for intubation or in-hospital death will be used to compute an estimate of the cause-specific hazard ratio along with a 95% confidence interval and a p-value.

To accompany this analysis, estimates of the cumulative incidence functions for the composite event of intubation or death will plotted for each arm

4.3.2.Ventilator-free days

The total number of ventilator-free days will be computed for each individual between randomization and Day 30. For each treatment group, the mean ventilator-free days will be computed by the sample average, and an associated robust bootstrap standard error will be obtained by resampling with replacement from the respective arm B=200 times. The difference in the sample means will be computed, along with a 95% confidence interval obtained based on the robust standard errors. A Wald test of the null hypothesis that there is no difference in the mean ventilator-free days between groups will be carried out using a robust variance obtained from the individual bootstrap standard errors.

4.3.3.In-hospital death by Day 90

The probability of in-hospital death within 90 days of randomization will be estimated for each treatment group by the proportion of individuals randomized to each group who died in hospital within the first 90 days of study. The primary analysis will be based on a 2-sided Wald test of the null hypothesis that probability of death by 90 days is the same among individuals

receiving CCP or standard care. The percent relative risk reduction will be estimated along with a 95% confidence interval and a p-value of the null hypothesis will be computed.

4.3.4. Time to in-hospital death

The time to in-hospital death will be analyzed using a cause-specific Cox regression model to deal with the competing risks of discharge from the hospital in which the individual was randomized; for subjects hospitalized for more than 90 days their time of in-hospital death will be censored at 90 days. That is, individuals who did not die in the hospital they were admitted to at the time of randomization will have their follow-up censored at the time of discharge from this hospital. The regression coefficient and associated standard error from the cause-specific Cox regression model for death in the original hospital will be used to compute an estimate of the cause-specific hazard ratio along with a 95% confidence interval and a p-value. To accompany this analysis estimates of the cumulative incidence functions will be plotted for in-hospital death and discharge for each treatment group.

4.3.5. Death by Day 30

The probability of death between randomization and Day 30 will be estimated for each treatment group by the proportion of individuals randomized who died between randomization and Day 30. The test will be based on a 2-sided Wald test of the null hypothesis that the probability of death by Day 30 is the same among individuals receiving CCP or standard of care. The percent relative risk reduction will be estimated along with a 95% confidence interval and a p-value of the null hypothesis will be computed.

4.3.6. Length of stay in intensive care unit

The total number of days in the intensive care unit (ICU) will be computed for each individual over the 30-day period from the time of randomization. For each treatment group, the mean number of ICU days will be computed by the sample average, and an associated robust bootstrap standard error will be obtained by resampling with replacement from the respective arm B=200 times. The difference in the sample means will be computed, along with a 95% confidence interval obtained based on the robust standard errors. A Wald test of the null hypothesis that there is no difference in the mean number of ICU days between groups will be carried out using a robust variance obtained from the individual bootstrap standard errors.

4.3.7. Length of stay in hospital

The length of stay in hospital over 90 days will be computed as the time from randomization to death or discharge disregarding the reason for the end of the hospitalization. A Cox regression model will then be fitted with this time as the response with an administrative censoring time set a 90 days from the day of randomization. The regression coefficient and associated standard error from the Cox regression model will be used to compute an estimate of the hazard ratio, along with a 95% confidence interval and a p-value. Kaplan-Meier estimates will be computed and plotted for the distribution of the time to the end of hospitalization for each arm of the study.

4.3.8.Need for Extra-Corporeal Membrane Oxygenation (ECMO)

The probability of requiring ECMO between randomization and Day 30 will be estimated for each treatment group by the proportion of individuals randomized to each group requiring ECMO between randomization and Day 30. The primary analysis will be based on a 2-sided Wald test of the null hypothesis that the probability of requiring ECMO is the same among individuals receiving CCP or standard of care. The percent relative risk reduction will be estimated along with a 95% confidence interval.

4.3.9.Need for renal replacement therapy

The probability of requiring renal replacement therapy between randomization and Day 30 will be estimated for each treatment group by the proportion of individuals randomized to each group requiring renal replacement therapy between randomization and Day 30. The primary analysis will be based on a 2-sided Wald test of the null hypothesis that the probability of requiring renal replacement therapy is the same among individuals receiving CCP or standard of care. The percent relative risk reduction will be estimated along with a 95% confidence interval.

4.3.10.Myocarditis

The probability of developing myocarditis over the first 30 days will be estimated in a similar fashion to the way that the need for ECMO and renal replacement therapy are estimated and tests of effects will likewise be reported in a similar fashion.

4.3.11.Patient-reported outcomes (PROs)

The original responses from each patient will be converted into a single utility index score anchored at 0 for dead and 1 for full health using the scoring algorithm based on Canadian society preferences. Utility changes from baseline to Day 30 were estimated by analysis of covariance adjusting for baseline utilities and compared between the two arms.

4.3.12.Incremental cost per quality-adjusted life year (QALY)

In addition to the cost of CCP, we will estimate the cost associated with the stay in ICU, hospitalization, use of ventilator and ECMO, diagnostics and procedures, and medications. The difference in cost between CCP and standard care will be divided by the difference in QALY calculated using the EQ-5D-5L to derive the incremental cost per QALY for CCP. 95% confidence interval for the incremental ratio will be assessed using a non-parametric bootstrapping with replacement approach with B=1000 times.

4.3.13. CPP transfusion-associated adverse events at Day 30

Adverse events judged to be definitely or probably related to CPP will be analyzed two ways: 1) using the Common Terminology Criteria for Adverse events (CTCAE; Grading 1-5)); and using the ISBT definitions for acute transfusions reactions (Grades 1-4). (Protocol section 4.3.13),

1. CTCAE Grading: The proportion of individuals experiencing CPP transfusion-associated adverse events (CTCAE Grades 1-5) will be reported overall by type, and severity. The total number of CPP transfusion-associated adverse

events will also be tabulated overall by type, and severity which will accommodate the possibilities of multiple adverse events per individual.

2. ISBT Grading: The proportion of individuals experiencing CPP transfusion-associated adverse events (ISBT Grades 1-4) will be reported overall by type, and severity. The total number of CPP transfusion-associated adverse events will also be tabulated overall by type, and severity which will accommodate the possibilities of multiple adverse events per individual.

4.3.12. Grade 3, 4, and 5 adverse events at Day 30
See protocol section 9 for additional details regarding AE definitions.

The proportion of individuals experiencing Grade 3, 4 or 5 (as per CTCAE criteria) adverse events will be analyzed overall and by treatment arm by type, severity grade, and expected/unexpectedness. The total number of Grade 3, 4 or 5 adverse events will also be tabulated overall and by treatment arm by type, severity grade and expected/unexpected which will accommodate the possibilities of multiple events per individual.

4.3.13. Grade 3, 4 and 5 serious adverse events at Day 30
Serious adverse events (unexpected and meeting criteria for expedited reporting to the sponsor, Hamilton Health Sciences) will be analyzed as summarized below. TRALI and TACO adverse events, while not technically considered SAEs, require expedited reporting to the sponsor; hence they will be also be included.

The number and proportion of individuals experiencing a serious adverse event will be reported by event type, overall and by treatment arm. A second analysis will be by event which will accommodate the possibilities of multiple events per individual.

In the interim and final analyses, the relative risk reduction for death (CCP versus standard of care) will be computed along with a 95% confidence interval. The relative risk reduction (CCP versus standard of care) will also be computed for SAEs (regardless of outcome) along with a 95% confidence interval.

The cumulative incidence of serious adverse event will also be plotted by treatment group.

4.4.Sub-Group Analyses
Subgroup analyses will be carried out to assess whether the effect of treatment varies across subgroups defined according to the following criteria:

- Patients enrolled vs. not enrolled/treated in other therapeutic clinical trials

- >= 60 years versus less than 60 years of age
- Sex
- Ethnicity
    - ➢ White/Black/Hispanic/Asian/Other

- Presence of medical co-morbidities
    - ➢ diabetes (yes/no)
    - ➢ cardiac (yes/no)
    - ➢ respiratory (yes/no)
- Smoking History
    - ➢ Ever smoker/current smoker/never
    - ➢ 15 pack year history of smoking versus not
- Obesity (BMI >= 30 vs <30)
- Onset of symptoms > 12 days before randomization (including non-respiratory symptoms) versus not
- Timing of administration of CCP from diagnosis of COVID-19 (<=72 hours vs >72 hours)
- Severity of illness of donors (hospitalized vs not)
- ABO blood type of recipients (A vs other, B vs other, O vs other, AB vs other)
- Detectable vs non-detectable viral plasma viral load at baseline
- Anti-SARS-CoV-2 seropositivity at baseline
- Oxygen status at baseline (≤ 2L vs > 2L)

These subgroup analyses will be carried out by estimating the treatment effect within sub-groups and providing 95% confidence intervals and p-values.

In addition the effect of plasma from one donor will be compared to the effect of plasma from two donors.

Any additional exploratory sub-group analyses carried out will be based on regression models involving treatment, the subgroup variable and the respective interaction. The findings from such post-hoc analyses will be reported as hypothesis generating if the test of the null hypothesis that the interaction term is zero gives a statistically significant finding of heterogeneity of the effect.

4.5.Safety Reports for the IDSMC

Monthly reports on logistical issues, donor recruitment, and patient accrual overall and by study site will be provided to the IDSMC. Information on antibody test results will also be provided as available. These reports will also give tabulations of i. adverse events, ii. serious adverse events, and iii. CPP transfusion-related adverse events. If the IDSMC requests more or less frequent reports on this data, they will be provided. The reports will be created by an independent statistician who is unblinded to the treatment assignment.

4.6.Exploratory Analysis of the Role of anti-SARS-CoV-2 antibodies

4.6.1.Neutralizing antibodies

Donor samples will be tested for the presence of total anti-SARS-CoV-2 antibodies and neutralizing antibodies for all CCP units transfused. Moreover, there will be samples taken from

study participants to assess antibody titre at the time of randomization and 48 hours later. The results of the donor antibody testing will be used to study the effect of neutralizing antibodies on clinical outcomes. The results of antibody testing of participants will enable investigation of the effect of CCP transfusion on participant antibody levels.

The total and neutralizing antibody titres for the donor samples will be recorded. In order to assess the effect-modifying role of these titres on the outcome, we will consider regression models for the primary outcome with covariates based on treatment arm, titres and interactions terms. For the primary binary outcome, a logistic regression model will be fitted involving the treatment indicator (equaling 1 for those in the CCP arm), an "interaction term" defined as the product of the treatment indicator and the log transformed and centered total antibody titre for the plasma transfused, and a second "interaction term" defined as the product of the treatment indicator and the log transformed and centered neutralizing antibody titre for the plasma transfused. The main effect of treatment will then correspond to the effect of CCP for a unit with the geometric mean total antibody titre and the geometric mean neutralizing antibody titre. The coefficient of the first "interaction term" will reflect how this CCP effect is moderated according to the total antibody titre for the plasma sample transfused, while the second "interaction term" will reflect how the CCP effect is moderated by the neutralizing antibody titre of the plasma sample transfused; the phrase "interaction term" is in quotation marks because the main effects for these titre based variables are not included in the linear predictor.

Similar analyses to those for the total and neutralizing antibodies will be carried out incorporating information of the patients own total and neutralizing antibody profiles prior to the plasma transfusion to explore how the benefit of CCP may vary depending on the antibody profile of the recipient. These analyses will be carried out at the interim analysis for the IDSMC and at the time of study completion.

For a fuller description of the handling of the laboratory data and analyses please see *Statistical Analysis Plan for A Randomized Open-Label Trial of CONvalescent Plasma for Hospitalized Adults with Acute COVID-19 Respiratory Illness: Supplement Regarding Antibody Analyses*

4.6.2.Viral load

A sample from study participants will be collected at the time of randomization (up to 48 hours before randomization) and 48 hours later (up to 5 days later) to measure RNA levels of SARS-CoV-2. This information will enable an analysis of the effect of CCP on viral load.

4.7.Schedule for Primary Interim Analysis

A single interim analysis is planned when information on intubation or death at Day 30 is available for 50% of the target sample (600 patients: 400 in the CCP arm and 200 in the arm receiving standard care). An O'Brien-Fleming stopping rule will be employed with a threshold for formal statistical significance at the interim analysis of 0.0054 and the final significance level of 0.0492. The Independent Data Safety Monitoring Committee (IDSMC) may recommend early

termination or modification of the protocol only when there is clear and substantial evidence of a treatment difference. More details about the interim analyses are described below as well as a separate guidance document for the IDSMC.

4.8.The Lan-DeMets Error Spending Function

In case any additional interim analyses are requested by the IDSMC, the flexible Lan-DeMets (Lan and DeMets, 1983) type I error spending function analog of the O'Brien-Fleming boundaries will be used to monitor the primary endpoint as a guide for the IDSMC for an overall two-sided type I error rate of 0.05 based on a two-sided test. This is defined as follows. If we let n denote the sample size according to the calculation for the planned study and n_o denote the number of individuals at an interim analysis, then let $t = n_o/n$. This “O'Brien-Fleming" error spending function has the form

$$\alpha(t) = 2 - 2\Phi(Z_{\alpha/2}/\sqrt{t})$$

where $Z_{\alpha/2} = 1.96$ reflects the critical value corresponding to the analysis that would be done for an analogous trial with a fixed sample size and a two-sided test (Lans and DeMets, 1983). For the interim analysis planned when 50% of the sample will be available *t = 1/2* and we obtain

$$\alpha(1/2) = 2(1 - \Phi(Z_{\alpha/2}/\sqrt{0.5})) = 0.005573$$

as the threshold for significance and stopping at the interim analysis. This constitutes a conservative stopping rule which we will adopt to preserve much of the type I error for the analysis at the conclusion of the study, to minimize the bias in estimates of the treatment effect from sequential trials, and to gear the trial towards one that will be large enough to answer the several important secondary questions we aim to address including the effect of neutralizing antibodies.

4.9.Interim Calculation of Conditional Power for Futility

Conditional power will be presented as an additional guide to the IDSMC for the primary outcome based on a test for the difference in two binomial proportions (Jennison and Turnbull, 2000). Conditional power allows computation of the probability of obtaining a statistically significant result by the end of the trial given the data accumulated thus far, incorporating a hypothesized treatment effect (e.g., the treatment effect assumed for sample size determination) thereafter. If the conditional power is less than 20% under the original trial assumptions, consideration could be given to stop the trial. An unblinded independent statistician will prepare the reports for IDSMC to review when making their recommendations.

The interim analysis report will be created by an independent statistician who will have access to the randomization code.

4.10. Sample Size Re-Estimation

There is uncertainty about the intubation and mortality rate that will be observed in this study due to possibly evolving profile of COVID-19 patients admitted to hospital over the course of the pandemic. To address this, a blinded sample size re-estimation will take place when the primary outcome is available for 50% of the target sample, which coincides with the time of the interim analysis report. Sample size will be adjusted if the estimated event rate is different from that originally specified to the extent that the study is not expected to achieve the desired nominal power for the test of the primary outcome. Friede and Kieser (2004) give the following formula for total sample size re-estimation: We let $n = n_e + n_s$ denote the total sample size where n_e and n_s denote the number of individuals in the experimental (CCP) and standard of care arms respectively, and let q denote the randomization ratio where $n_s = \theta n_e = 0.5 n_e$. We let d be the absolute risk difference we wish to detect (which was specified in the original sample size calculation). Then if we observe a pooled composite event rate *p*, then

$$n = n_e + n_s = \left(2 + \theta + \frac{1}{\theta}\right)\frac{\left(Z_{\alpha/2} + Z_{\beta}\right)^2}{\delta^2}p(1-p),$$

gives the revised total sample size.

4.11. Conventions Regarding Blinding

It is generally recognized that blinding of the investigators to the arm-specific data is desirable during the course of a clinical trial. The monthly safety reports being prepared for the IDSMC will display data on trial conduct aggregated across centers and specific to each center, and will give unblinded safety data in order to equip them to assess safety.

At the point of the interim analysis being conducted when 50% of the target sample size can provide primary outcome data, again the data will be presented with the randomized treatment arm clearly specified in order to equip the IDSMC with the information needed to interpret the results of the interim analyses.

A blinded version of the monthly Progress and Safety Report will be created for the Executive Committee in which the data will only be presented in such a way that the treatment arm cannot be identified; this will be achieved by using generic labels A and B for the two arms, not reporting the sample sizes for these groups, and reporting simply the percentages of individuals experiencing particular events, averages, and standard deviations rather than standard errors for continuous variables. For some features data will only be presented in aggregate form.

The sample size re-estimation will be conducted by a blinded investigator (R. Cook) based on data aggregated across the two arms as described in Sections 4.9. The rationale is that this calculation is the responsibility of the investigators and the investigators ideally remain blinded to the interim outcomes.

As the independent statistician preparing the interim analysis report is unblinded and the IDSMC members are unblinded, they will be equipped to assess the suitability of the revised target sample size.

5. References

Friede T and Kieser M (2004). Sample size recalculation for binary data in internal pilot study designs. *Pharmaceutical Statistics*, 3: 269-279.

Herdman M, Gudex C, Lloyd A, et al (2011). Development and preliminary testing of the new five-level version of EQ-5D (EQ-5D-5L). *Quality of Life Research,* 20(10):1727-1736.

Jennison C and Turnbull BW (2000). *Group Sequential Methods with Applications to Clinical Trials*. Chapman and Hall, Boca Raton.

Kalbfleisch JD and Prentice RL (2011). *The Statistical Analysis of Failure Time Data (Vol. 360)*. John Wiley and Sons.

Kenward MG and Carpenter J (2007). Multiple imputation: current perspectives. *Statistical Methods in Medical Research*, 16(3), 199-218.

Lan K and DeMets D (1983). Discrete sequential boundaries for clinical trials. *Biometrika*, 70: 659-663.

Little RJA and Rubin DB (2002). *Statistical Analysis with Missing Data, 2nd edition*. Wiley

O'Brien P and Fleming T (1979). *Multiple Testing Procedure for Clinical Trials*. Biometrics, 35: 549-556.

Rubin DB. (1996). Multiple imputation after 18+ years. *Journal of the American Statistical Association*, 91: 473-490.

Statistical Analysis Plan for

A Randomized Open-Label Trial of CONvalescent Plasma for Hospitalized Adults With Acute COVID-19 Respiratory Illness: Supplement regarding Antibody Analyses

(CONCOR-1)

**Co-Lead Principal Investigators:**	Dr. Donald Arnold<br>Dr. Philippe Bégin<br>Dr. Jeannie Callum
**Investigational product:**	COVID-19 Convalescent Plasma
**Protocol #:**	GRAAL-2020-01
**Auxiliary SAP for Protocol :**	Version v8.0 15 Jan 2021

The study will be conducted according to the protocol and in compliance with Good Clinical Practice (GCP), with the Declaration of Helsinki and with other applicable regulatory requirements.

This document contains confidential information.
Do not copy or distribute without written permission from the Sponsor.

CONFIDENTIAL

Contents

1 Laboratory Markers To Be Examined as Possible Effect Modifiers 1
1.1 Anti-RBD total Ig as Measured by ELISA 1
1.2 Anti-SPIKE IgG as Measured by Flow Cytometry 1
1.3 Plaque Reduction Neutralization Titer 50% (PRNT50) 1
1.4 Antibody-Dependent Cell Cytotoxicity (ADCC) 1
1.5 Combining Antibody Data from Two Plasma Units 2
1.5.1 Dealing with Replicate Measurements on a Given Plasma Unit 2
1.5.2 Combination of Test Results from Different Plasma Units 2
1.6 Log Transformation Before Analysis 2
1.7 Handling Missing Data 2
1.7.1 Missing Unit Volumes 2
1.7.2 Missing Markers for Particle Providers 2
1.7.3 Results missing for markers when measurements are expected 3

2 Statistical Analyses Involving Laboratory Data 3
2.1 Regression Analyses Investigating Role of Markers in the CCP Arm 3
2.2 Regression Analyses Exploring the Effect-Modifying Role of Each Marker 4
2.3 Multivariate Regression Analyses of Effect-Modifying Roles of Markers 4
2.4 Incorporating the Patient Marker Values 5
2.4.1 Exploring the Effect of Patient Marker Values 5
2.4.2 Incorporating the Patient Marker Values - Effect Modifying Roles 5

3 References 5

1 Laboratory Markers To Be Examined as Possible Effect Modifiers

Here we give a clear description of the markers under consideration that are measured in the convalescent plasma transfused in the CCP arm of CONCOR-1.

1.1 Anti-RBD total Ig as Measured by ELISA

This marker is reported as the optic density (O.D.), which is a measure directly proportional to the amount of antibody. This is recorded into the REDCap database directly.

1.2 Anti-SPIKE IgG as Measured by Flow Cytometry

This marker is reported as the mean fluorescence intensity (MFI), which is directly proportional to the amount of antibody. This is recorded in the REDCap database directly.

1.3 Plaque Reduction Neutralization Titer 50% (PRNT50)

This marker is reported as the highest titer at which the plasma still inhibits at least 50% of viral entry in cells in a plaque reduction assay. Titers are determined by doubling the dilution at each stage, leading to discrete values ranging from 1:20 to $\geq$1:640 (or $\geq$ 1:1280 for some plasma samples tested at the beginning of the pandemic). Before uploading into REDCap:

- Left-censored values will be dealt with via mid-point imputation so if 50% of the viral entry in cells is not inhibited at the first dilution, then the value is taken as 1 : 10 where 10 is halfway between 0 and 20.
- Right-censored values where changed as follows:
  - If it was determined that 50% of the cells where inhibited at at the highest dilution of 640 (i.e. we know it is $\geq$ 1:640), the value was taken to be 1:640
  - If it was determined that 50% of the cells where inhibited at at the highest dilution of 1280 (i.e. we know it is $\geq$ 1:1280), the value was taken to be 1:1280
- Ultimately the dilution rate was inverted to express titers in terms of the number of dilutions so a titer of 1:40 was expressed as 40 dilutions

1.4 Antibody-Dependent Cell Cytotoxicity (ADCC)

This marker is reported as the proportion of the target cells that were killed by NK cells in presence of the antibody. Before uploading in REDCap the ADCC proportion X was re-expressed as the ADCC ratio to give $Y = X/(1 - X)$ (e.g. a value of 66% was expressed as 2 corresponding to a 66.66:33.33 = 2:1 ratio).

1.5 Combining Antibody Data from Two Plasma Units

1.5.1 Dealing with Replicate Measurements on a Given Plasma Unit

When multiple samples from the same donor plasma have been tested, the mean result was uploaded in REDCap. For the ADCC marker the averaging will be carried out on the scale of the proportion and then the ratio will be computed based on this average proportion.

1.5.2 Combination of Test Results from Different Plasma Units

All test values are entered into REDCap are measures of concentrations expressed on an absolute scale. Test results from the two different plasma units transfused to an individual will be combined as follows. A weighted sum of the two concentrations will be computed with weights based on the volume of the respective units. That is, we will compute an estiamte of the "total antibody dose" for the pooled sample as

```
Total Dose = (Vol. 1 ÃŮ Conc. 1 + Vol. 2 ÃŮ Conc. 2)
```

Example: Consider two units and the anti-RBD ELISA results with the first 235 ml having an O.D. of 0.25, and the second unit of 240 ml having an OD of 1.06. The overall OD is computed as

$$\text{Total Dose} = 235 \times 0.25 + 240 \times 1.06 = 313.15$$

This patient would be considered to have received the equivalent total dose of antibodies corresponding to a 500 ml plasma transfusion with an anti-RBD antibodies at $313.15/500 = 0.63$ optical density.

1.6 Log Transformation Before Analysis

Final marker values representing concentrations will be log-transformed ($\log_{10}$) before being entered in statistical analysis assessing their pharmacological effect. So with a concentration of 0.63 we will use $\log_{10}(0.63) = -0.20$.

1.7 Handling Missing Data

1.7.1 Missing Unit Volumes

When the volume of a plasma unit is missing, the median volume of convalescent plasma units from the same blood provider will be imputed.

1.7.2 Missing Markers for Particle Providers

When marker values are not available for a plasma provider, indivdiuals receiving plasma from that provider will be excluded from the related analysis.

1.7.3 Results missing for markers when measurements are expected

When a marker value was expected but it is not available, multiple imputation will be used to impute the missing value based on a linear model in which imputation is carried out based on a regression model for the missing marker given the values of the available markers. When multiple markers are missing then imputation will be carried out using multiple imputation based on chained equations (White et al. 2011) with 10 imputed samples. The imputation models will be based on the joint distributions of all four markers under consideration.

2 Statistical Analyses Involving Laboratory Data

As a preliminary analysis we will tabulate the availability of marker data by site, blood provider, and according to the type of the marker. A careful description of the units used to measure the markers will be carried out with any change of units carried out as required. Upon standardization of the scales the summary statistics will be computed for each marker by site, by provider, and overall. All of the analyses that follow will control for the provider as a factor variable to ensure that comparisons are made within providers. This variable is suppressed in the model statements that follow for convenience.

2.1 Regression Analyses Investigating Role of Markers in the CCP Arm

Preliminary analyses will be directed at modeling the relations between the laboratory profile of CCP and the probability of experiencing the primary outcome. This will be done initially in a univariate fashion and then via multiple regression with several markers. Let $Y_i = 1$ if individual i is intubated or dies within 30 days and let M_{ij} be the jth marker of interest, $j = 1, \ldots, J$, $i = 1, \ldots, n$. Let $\pi(M_{ij}) = P(Y_i = 1|M_{ij})$ and

$$\log\left(\frac{\pi(M_{ij})}{1-\pi(M_{ij})}\right) = \alpha_0 + \alpha_1 M_{ij}\,,$$

be considered in the univariate analysis of marker j, $j = 1, \ldots, J$, and

$$\log\left(\frac{\pi(M_{i})}{1-\pi(M_{i})}\right) = \alpha_0 + \alpha_1 M_{i1} \cdots + \cdots \alpha_J M_{iJ}$$

be considered in the multivariate analysis where $M_i = (M_{i1}, \ldots, M_{iJ})'$ is the vector of markers. Tests for interactions will also be carried out to assess whether marker effects varying according to the value of other markers. Finally generalized additive models will be considered to give graphical plots of the relations between the marker values and the odds of response (Hastie and Tibshirani, 1990).

2.2 Regression Analyses Exploring the Effect-Modifying Role of Each Marker

The results of the antibody titre will give an indication of the amount of antibodies in the plasma transfused thereby enabling an investigation of the effect the amount of neutralizing antibodies has on the effect of CCP versus standard care. For each marker the value will be centered by subtracting the mean value among the plasma units transfused. For the primary binary response, a logistic regression model (McCullaugh and Nelder, 1989) will be fitted involving the treatment indicator (equalling 1 for those in the CCP arm) and an interaction between the treatment indicator and the centered marker. The coefficient of the treatment indicator will reflect the effect of CCP transfusion at the mean level of the marker, while the coefficient of the interaction term will reflect how this effect is modified according to the marker value in the transfused plasma. The details are given in what follows.

Let $Y_i = 1$ if individual i is intubated or dies within 30 days and let Z_i be their covariate vector, $i = 1, \ldots, n$. Let $\pi(Z_i) = P(Y_i = 1|Z_i)$ where $Z_i' = (1, X_i, X_i M_i)$ where X_i indicates randomization to the CCP arm and M_i is the marker level in the CCP.

$$\log\left(\frac{\pi(Z_i)}{1-\pi(Z_i)}\right) = \beta_0 + \beta_1 X_i + \beta_2 X_i M_i \,.$$

In order to relax the assumption of a linear effect-modifying role of the marker, generalized additive models (Hastie and Tibshirani, 1990) will be fitted for the effect of $X_i M_i$ with the results to give insight into critical threshold levels of the markers.

2.3 Multivariate Regression Analyses of Effect-Modifying Roles of Markers

Following the exploration of the effect-modifying roles of the J individual marker values, multivariate models will be fitted to investigate whether there are critical compbinations of levels across the markers which identify CCP which is particularly effective in reducing the odds of the primary response. For this we let M_{ij} denote the jth marker, $j = 1, \ldots, J$ and $Z_i = (X_i, M_{i1}, \ldots, M_{iJ})'$. Moreover we consider

$$\log\left(\frac{\pi(Z_i)}{1-\pi(Z_i)}\right) = \beta_0 + \beta_1 X_i + \beta_2 X_i M_{i1} \cdots + \cdots \beta_{J+1} X_i M_{iJ} \,.$$

and test the significance of the interaction terms $\beta_2, \ldots, \beta_{J+1}$. Again generalized additive models will be explored to provide graphical displays conveying the nature of the effect-modifying role of the marker.

2.4 Incorporating the Patient Marker Values

2.4.1 Exploring the Effect of Patient Marker Values

Here we consider patients in the CCP arm along but let M_{ij}^P denote the marker value for the jth marker measured in the patient, assumed to be centered. Let $Z_{ij} = (M_{ij}, M_{ij}^P)'$ for a particular marker under study. Here we consider interactions between the marker value in the transfused plasma and the marker value in the patient in models given by

$$\log\left(\frac{\pi(Z_{ij})}{1-\pi(Z_{ij})}\right) = \theta_0 + \theta_1 M_{ij} + \theta_2 M_{ij}^P + \theta_3 M_{ij} M_{ij}^P \, ,$$

$j = 1, \ldots, J$.

2.4.2 Incorporating the Patient Marker Values - Effect Modifying Roles

Let M_{ij}^P denote the marker value for the jth marker measured in the patient, as opposed to the unit transfused, $j = 1, \ldots, J$. Additional analyses will introduce the patient markers denoted M_{ij}^P into the analyses, which are assumed to be centered values. Let $Z_{ij} = (X_i, M_{ij}, M_{ij}^P)'$ for a particular marker under study. Here we consider interactions between the marker value in the transfused plasma and the marker value in the patient in models given by

$$\log\left(\frac{\pi(Z_{ij})}{1-\pi(Z_{ij})}\right) = \gamma_0 + \gamma_1 X_i + \gamma_2 X_i M_{ij} + \gamma_3 X_i M_{ij}^P + \gamma_4 X_i M_{ij} M_{ij}^P \, .$$

Again multivariate models involving several markers can be considered if the above marker-specific analyses yield evidence that more than one marker may be critical in determining the effect of the convalescent plasma.

3 References

Hastie TJ (1992). *Generalized Additive Models.* Chapter 7, *Statistical Models in S*, Eds. JM Chambers and TJ Hastie, Wadsworth & Brooks/Cole.

Hastie T and Tibshirani R (1990). *Generalized Additive Models.* London: Chapman and Hall.

McCullaugh P and Nelder J (1989). *Generalized Linear Models.* London: Chapman and Hall.

Venables WN and Ripley BD (2002). *Modern Applied Statistics with S.* New York: Springer.

White IR, Royston P, and Wood AM (2011). Multiple imputation using chained equations: issues and guidance for practice. *Statistics in Medicine*, 30(4), 377-399.
