# Supplementary material for: Convalescent plasma for hospitalized patients with COVID-19: an open-label, randomized controlled trial
Source: Nat Med. 2021 Sep 9;27(11):2012–24. doi: 10.1038/s41591-021-01488-2 (PMC8604729; doi:10.1038/s41591-021-01488-2)
Supplement: Supplementary file 2 — Reporting Summary [file 41591_2021_1488_MOESM2_ESM.pdf]

nature portfolio

Corresponding author(s): Philippe Bégin
Jeannie Callum
Donald Arnold

Last updated by author(s): Jul 29, 2021

Reporting Summary

Nature Portfolio wishes to improve the reproducibility of the work that we publish. This form provides structure for consistency and transparency in reporting. For further information on Nature Portfolio policies, see our Editorial Policies and the Editorial Policy Checklist.

Statistics

For all statistical analyses, confirm that the following items are present in the figure legend, table legend, main text, or Methods section.

n/a	Confirmed	
☐	☒	The exact sample size (*n*) for each experimental group/condition, given as a discrete number and unit of measurement
☐	☒	A statement on whether measurements were taken from distinct samples or whether the same sample was measured repeatedly
☐	☒	The statistical test(s) used AND whether they are one- or two-sided<br>*Only common tests should be described solely by name; describe more complex techniques in the Methods section.*
☐	☒	A description of all covariates tested
☐	☒	A description of any assumptions or corrections, such as tests of normality and adjustment for multiple comparisons
☐	☒	A full description of the statistical parameters including central tendency (e.g. means) or other basic estimates (e.g. regression coefficient) AND variation (e.g. standard deviation) or associated estimates of uncertainty (e.g. confidence intervals)
☐	☒	For null hypothesis testing, the test statistic (e.g. *F*, *t*, *r*) with confidence intervals, effect sizes, degrees of freedom and *P* value noted<br>*Give P values as exact values whenever suitable.*
☒	☐	For Bayesian analysis, information on the choice of priors and Markov chain Monte Carlo settings
☒	☐	For hierarchical and complex designs, identification of the appropriate level for tests and full reporting of outcomes
☐	☒	Estimates of effect sizes (e.g. Cohen's *d*, Pearson's *r*), indicating how they were calculated

Our web collection on statistics for biologists contains articles on many of the points above.

Software and code

Policy information about availability of computer code

Data collection	RedCAP v11.0.1
Data analysis	SAS 9.4 (Cary, NC, USA) and R 4.0.2 (R Core Team) with the mgcv package v1.8-36

For manuscripts utilizing custom algorithms or software that are central to the research but not yet described in published literature, software must be made available to editors and reviewers. We strongly encourage code deposition in a community repository (e.g. GitHub). See the Nature Portfolio guidelines for submitting code & software for further information.

Data

Policy information about availability of data

All manuscripts must include a data availability statement. This statement should provide the following information, where applicable:

- Accession codes, unique identifiers, or web links for publicly available datasets
- A description of any restrictions on data availability
- For clinical datasets or third party data, please ensure that the statement adheres to our policy

De-identified individual patient data with the data dictionary that underlie the reported results will be made available upon request if the intended use is concordant with the existing research ethics board approvals (requests will be reviewed by the CONCOR-1 Steering Committee within three months). Proposals for access should be sent to arnold@mcmaster.ca. The protocol and statistical analysis plan are available in the online supplement.

Field-specific reporting

Please select the one below that is the best fit for your research. If you are not sure, read the appropriate sections before making your selection.

☒ Life sciences ☐ Behavioural & social sciences ☐ Ecological, evolutionary & environmental sciences

For a reference copy of the document with all sections, see nature.com/documents/nr-reporting-summary-flat.pdf

Life sciences study design

All studies must disclose on these points even when the disclosure is negative.

Sample size	With a 2:1 randomization ratio, 1200 patients (800 in the convalescent plasma group, and 400 in the standard of care group) were needed to provide 80% power to detect a relative risk reduction of 25% with convalescent plasma for the primary outcome with a 30% event rate under standard of care, based on a two-sided test at the 5% significance level. Analyses for secondary and exploratory outcomes were performed on the complete set of available data.
Data exclusions	No data was excluded from the intent-to-treat analysis. Reasons for exclusion from the per protocol analysis included inelegibility for the trial (n=9); patients that did not receive CCP (n=17); that received less than 400mL of CCP (n=11); and CCP transfusion that ended more than 24h after transfusion, as per the study protocol.
Replication	Findings were not replicated as this was a clinical trial.
Randomization	Patients were randomised in a 2:1 ratio to receive convalescent plasma or standard of care using a secure, concealed, computer-generated, web-accessed randomisation sequence (REDCap). Randomisation was stratified by site and age (<60 and ≥ 60 years) with allocation made with permuted blocks of size 3 or 6.
Blinding	This was an open label trial. The statistician performing the final analysis remained blinded throughout the trial. An open-label design was justified since the primary outcomes (intubation or death) are objective in nature. In addition, masking procedures such as plasma bag covers and additional labeling of plasma units would impose significant challenges to blood bank personnel during the pandemic, which would have made the trial infeasible in many centres. The use of standard plasma as the control was not felt to be justified because of the potential harm with no anticipated benefit.

Reporting for specific materials, systems and methods

We require information from authors about some types of materials, experimental systems and methods used in many studies. Here, indicate whether each material, system or method listed is relevant to your study. If you are not sure if a list item applies to your research, read the appropriate section before selecting a response.

Materials & experimental systems

n/a	Involved in the study	
☐	☒	Antibodies
☐	☒	Eukaryotic cell lines
☒	☐	Palaeontology and archaeology
☒	☐	Animals and other organisms
☐	☒	Human research participants
☐	☒	Clinical data
☒	☐	Dual use research of concern

Methods

n/a	Involved in the study	
☒	☐	ChIP-seq
☒	☐	Flow cytometry
☒	☐	MRI-based neuroimaging

Antibodies

Antibodies used	For PRNT: VHH72hFc from the NRC (lot # AP200512),<br>For ADCC, CR3022 and CV3-13 WT, both produced in the Finzi lab, first described in PMID:16796401 and PMID:34237283, respectively.<br>For ELISA, CR3022 as described above and 109-035-064 from Jackson ImmunoResearch Laboratories Inc, (lot#143450)
Validation	For VHH72hFc, see PMID: 32870820<br>For Jackson 109-035-064, see lot#143450 at https://www.jacksonimmuno.com/catalog/products/109-035-064<br>For CR3033 and CV3-13, the antibodies were separated by SDS-PAGE with and without the presence of 2-Mercaptoethanol. Furthermore, for each experiment, CR3022 and CV3-13 WT were validated by flow cytometry to make sure they recognized the Spike of SARS-CoV-2.

Eukaryotic cell lines

Policy information about cell lines

Cell line source(s)	Vero E6 cells from ATCC laboratory;<br>293T and CEM.NKr-CCR5 parental cells from ATCC laboratory;<br>CEM.NKr-SARS-CoV-2.Spike and 293T.SPike cells were produced in the Finzi Lab (PMID:33969322)
Authentication	Certificate of analysis for Vero E6 cells available at https://www.atcc.org/products/crl-1586; lot number 63290666<br>Certificate of analysis for 293T cells available at https://www.atcc.org/products/crl-3216; lot number 70023985<br>Certificate of analysis for CEM.NKr-CCR5 cells available at https://www.hivreagentprogram.org/Catalog/cellBanks/ARP-4376.aspx; lot number 150240
Mycoplasma contamination	Vero E6 and 293T cells were tested negative for mycoplasma; CEM.NKr-SARS-CoV-2.Spike cells were not tested for mycoplasma.
Commonly misidentified lines<br>(See ICLAC register)	No commonly misidentified cell lines were used in the study

Human research participants

Policy information about studies involving human research participants

Population characteristics	Baseline demographics were balanced between groups for all study populations. Median age was 69 years, with 59% male and 41% female and the median time from the onset of any COVID-19 symptom was 8 days (interquartile range, 5 to 10). The majority of participants (84.0%) were receiving systemic corticosteroids at the time of enrolment.
Recruitment	Participants were partients admitted to the ward or intensive care unit of participating hospitals. Institution-specific systematic screening of newly admitted COVID-+ patients was used to identify potential patients. After permission to approach the patient was obtained from a member of the clinical circle of care, participants or their substitue decision makers were approached for consent by research personnel either in person or by phone. Screening logs were kept and reviewed on a regular basis to identify and prevent selection bias.
Ethics oversight	The study was approved by Clinical Trials Ontario (research Ethics Board of Record: Sunnybrook Health Sciences Centre); the Quebec Ministry of Health and Social Services multicer ethics review (REB of Record: Comité d'éthique de la recherche du CHU Sainte-Justine); the Brazilian Comissao Natcional de Ética em Pesquisa, the Héma-Québec Research Ethics Board, the Canadian Blood Services research ethics board; The Weil Cornell Medicine General Institutional Review Board; Research Ethics BC (REB of record: The University of British Columbia Clinical Research Ethics Board), The Conjoint Health Research Ethics Board (CHREB), University of Alberta Health Research Ethics Board (Biomedical Committee), Saskatchewan Health Authority Research Ethics Board, University of Saskatchewan-Biomedical Research Ethics Board (Bio-REB), University of Manitoba(UM) Biomedical Research Board (BREB), Queensway Carleton Hospital Research Ethics Board, Scarborough Health Network Research Ethics Board, Windsor Regional Hospital Research Ethics Board and the Bureau de l'Éthique of Vitalité Health Network.

Note that full information on the approval of the study protocol must also be provided in the manuscript.

Clinical data

Policy information about clinical studies

All manuscripts should comply with the ICMJE guidelines for publication of clinical research and a completed CONSORT checklist must be included with all submissions.

Clinical trial registration	NCT04348656
Study protocol	The full trial protocol is included in the online supplement.
Data collection	Patients were enrolled between May 14th 2020 and January 29th 2021. Data was collected from baseline (day 1) to day 30 and in-hospital mortality data was collected up to day 90 for patients still in hospital at day 30. Data was collected by the research staff at each study site from patients or their substitute decision makers, from clinical staff and from the patient's health record and entered into a web-based electronic data collection system (REDCap). The patient's health record and study specific Case Report Forms served as source documents.
Outcomes	The primary outcome was the composite of intubation or death by day 30. Secondary outcomes were: time to intubation or death; ventilator-free days by day 30; in-hospital death by day 90; time to in-hospital death; death by day 30; lenght of stay in critical care and hospital; need for extracorporeal membrane oxygenation; need for renal replacement therapy; convalescent plasma-associated adverse events; occurence of adverse events graded 3 or higher by day 30. Primary and secondary efficacy outcomes were assessed through a review of the clinical charts and study CRFs. Classification of adverse events was performed using MedDRA (https://www.meddra.org/) and graded by the Common Terminology Criteria for Adverse Events, version 4.03. All transfusion-related adverse events were classified and graded by the international Society for Blood Transfusion definitions (www.isbtweb.org). A complete description of the outocomes and their measurements is described in the Statistical Analysis Plan.
